# Supplementary material for: NPSV: A simulation-driven approach to genotyping structural variants in whole-genome sequencing data
Source: Gigascience. 2021 Jul 1;10(7):giab046. doi: 10.1093/gigascience/giab046 (PMC8246072; doi:10.1093/gigascience/giab046)
Supplement: giab046_GIGA-D-20-00373_Revision_1 [file giab046_giga-d-20-00373_revision_1.pdf]

GigaScience

NPSV: A simulation-driven approach to genotyping structural variants in whole genome sequencing data

--Manuscript Draft--

**Manuscript Number:**	GIGA-D-20-00373R1
**Full Title:**	NPSV: A simulation-driven approach to genotyping structural variants in whole genome sequencing data
**Article Type:**	Technical Note
**Funding Information:**	National Institute of General Medical Sciences (P20GM103449) — Dr. Michael Linderman<br>National Heart, Lung, and Blood Institute (UM1HL098123) — Dr. Michael Linderman<br>National Science Foundation (1827373) — Dr. Michael Linderman
**Abstract:**	Background: Structural variants (SV) play a causal role in numerous diseases but are difficult to detect and accurately genotype (determine zygosity) in whole genome next-generation sequencing (NGS) data. SV genotypers that assume the aligned sequencing data uniformly reflects the underlying SV or use existing SV call sets as training data can only partially account for variant and sample-specific biases.<br>Results: We introduce NPSV, a machine learning-based approach for genotyping previously discovered SVs that employs NGS simulation to model the combined effects of the genomic region, sequencer and alignment pipeline on the observed SV evidence. We evaluate NPSV alongside existing SV genotypers on multiple benchmark call sets. We show that NPSV consistently achieves or exceeds state-of-the-art genotyping accuracy across SV call sets, samples and variant types. NPSV can specifically identify putative de novo SVs in a trio context and is robust to offset SV breakpoints.<br>Conclusions: Growing SV databases and the increasing availability of SV calls from long-read sequencing make stand-alone genotyping of previously identified SVs an increasingly important component of genome analyses. By treating potential biases as a simulate-able “black box” NPSV provides a framework for accurately genotyping a broad range of SVs in both targeted and genome-scale applications.
**Corresponding Author:**	Michael Linderman<br><br>UNITED STATES
**Corresponding Author Secondary Information:**	
**Corresponding Author's Institution:**	
**Corresponding Author's Secondary Institution:**	
**First Author:**	Michael Linderman
**First Author Secondary Information:**	
**Order of Authors:**	Michael Linderman
	Crystal Paudyal
	Musab Shakeel
	William Kelley
	Ali Bashir
	Bruce D. Gelb
**Order of Authors Secondary Information:**	

Powered by Editorial Manager® and ProduXion Manager® from Aries Systems Corporation

Response to Reviewers:	Please see uploaded document.
Additional Information:	
Question	Response
Are you submitting this manuscript to a special series or article collection?	No
**Experimental design and statistics**<br><br>Full details of the experimental design and statistical methods used should be given in the Methods section, as detailed in our Minimum Standards Reporting Checklist. Information essential to interpreting the data presented should be made available in the figure legends.<br><br>Have you included all the information requested in your manuscript?	Yes
**Resources**<br><br>A description of all resources used, including antibodies, cell lines, animals and software tools, with enough information to allow them to be uniquely identified, should be included in the Methods section. Authors are strongly encouraged to cite Research Resource Identifiers (RRIDs) for antibodies, model organisms and tools, where possible.<br><br>Have you included the information requested as detailed in our Minimum Standards Reporting Checklist?	Yes
**Availability of data and materials**<br><br>All datasets and code on which the conclusions of the paper rely must be either included in your submission or deposited in publicly available repositories (where available and ethically appropriate), referencing such data using a unique identifier in the references and in the "Availability of Data and Materials" section of your manuscript.	Yes

Have you have met the above requirement as detailed in our Minimum Standards Reporting Checklist?	

Powered by Editorial Manager® and ProduXion Manager® from Aries Systems Corporation

Manuscript which change tracking
Click here to access/download;Manuscript;NPSVManuscriptRevision1.pdf

NPSV: A simulation-driven approach to genotyping structural variants in whole genome sequencing data

Running Head: Genotyping SVs with simulation

Michael D. Linderman,[1*] Crystal Paudyal,[1] Musab Shakeel,[1] William Kelley,[1] Ali Bashir[2†], Bruce D. Gelb[3†]

[1]Department of Computer Science, Middlebury College, Middlebury, VT, USA
[2]Google, Mountain View, CA, USA
[3]Mindich Child Health and Development Institute and the Departments of Pediatrics and Genetics and Genomic Sciences, Icahn School of Medicine at Mount Sinai, New York, NY, USA

[†]These authors contributed equally

*Corresponding Author:

Michael D. Linderman
mlinderman@middlebury.edu
Department of Computer Science
Middlebury College
14 Old Chapel Road
Middlebury, VT 05753
(802) 443-5737

Abstract

Background: Structural variants (SV) play a causal role in numerous diseases but are difficult to detect and accurately genotype (determine zygosity) in whole genome next-generation sequencing (NGS) data. SV genotypers that assume the aligned sequencing data uniformly reflects the underlying SV or use existing SV call sets as training data can only partially account for variant and sample-specific biases.

Results: We introduce NPSV, a machine learning-based approach for genotyping previously discovered SV~~s genotyping~~ that employs NGS simulation to model the combined effects of the genomic region, sequencer and alignment pipeline on the observed SV evidence. We evaluate NPSV alongside existing SV genotypers on multiple benchmark call sets. We show that NPSV consistently achieves or exceeds state-of-the-art genotyping accuracy across SV call sets, samples and variant types. NPSV can specifically identify putative *de novo* SVs in a trio context and is robust to offset SV breakpoints.

Conclusions: Growing SV databases and the increasing availability of SV calls from long-read sequencing make stand-alone ~~SV~~ genotyping of previously identified SVs an increasingly important component of genome analyses. By treating potential biases as a simulate-able “black box” NPSV provides a framework for accurately genotyping a broad range of SVs in both targeted and genome-scale applications.

Keywords

Structural variants, Next generation sequencing, Whole genome sequencing

Findings

Background

Structural variants (SVs) play a causal role in numerous diseases[1]. However, our ability to detect and analyze disease-causing SVs in short-read whole genome sequencing (WGS) data can be limited by inaccurate genotyping (determining zygosity)[2,3]. While numerous tools integrate SV discovery and genotyping[4–6], our focus here is "stand-alone" genotyping of putative SVs identified by discovery tools and/or obtained from the literature/SV catalogs [7]. Stand-alone genotyping is a critical step in ensemble pipelines that integrate multiple SV discovery tools, in clinical workflows, where we seek to accurately genotype known pathogenic SVs (e.g., from dbVar[8]) alongside detecting novel SVs, and in population studies, which generate "squared-off" genotypes for all variants in all samples[7].

SVs, defined here as variants greater than 50 bp[9], are similar in size to or larger than the read length of short-read next generation sequencers (NGS) and, thus, typically cannot be detected directly. Instead SVs must be inferred from secondary features in the sequencing data such as split reads, discordant read-pairs and read depth[9]. As a result, the precision and recall for detecting and genotyping SVs in NGS data can be much lower than for single nucleotide variants and short indels[4,7,10–13]. Long-read sequencing (read lengths of 10+ kbp) improves the recall and precision of SV detection (the long reads span more events and can be more reliably mapped)[14–16]. However, long-read sequencing is more expensive than NGS[17], so many more samples have been and will continue to be sequenced with NGS technologies. Thus, despite the growth in long-read sequencing, there is a need to develop improved NGS SV

genotyping tools, including to genotype those SVs first (and exclusively) detected with long-read sequencing.

Existing SV genotyping tools[18–26] (see Chander et al.[7] for a recent comparison) exclusively target specific variant types/sizes, employ parametric (i.e., fixed-size[27]) models of SV evidence, and/or are trained on existing genome-wide call sets. These approaches assume that different SV call sets are similar and/or that the aligned sequencing data consistently and uniformly reflects the underlying variant (e.g., the read depth is proportional to copy-number, alternate alleles are identified at a consistent rate and/or consistent breakpoint features will be observed across all variants). However, these assumptions do not hold for all variants. The different types of SVs, range of SV sizes, different genomic contexts and different sequencers/pipelines, all of which influence the available evidence for predicting the SV genotype, motivate an ensemble of approaches, each optimized for a specific subset of SVs[10,28]. NGS simulation is a possible strategy to enable automatic ensemble creation. For example, for select SVs from the 1000 Genomes Project, Chu *et al.* showed that training the GINDEL SV genotyper on simulated data could achieve genotyping accuracy within a few percentage points of models trained on held-out data[29].

Here, we propose the Non-Parametric SV (NPSV) genotyper. NPSV extends current ensemble methods by automatically creating classifiers for predicting SV genotypes optimized for the specific SVs and sample under analysis and even a single, specific, SV. In this non-parametric approach, the number of models can grow to capture genomic-region, sequencer and pipeline-specific SV evidence. NPSV performs detailed simulation of the putative SVs to be genotyped. The simulated data, which are representative of the actual observed sequencing data, are used to train sample- and variant-specific classifiers for predicting SV genotypes. In contrast to training

data sourced from existing SV call sets, by using simulation we can generate representative training data for any putative SV, not just those previously observed, with accurate sequence-resolved breakpoints and “ground truth” genotype labels.

We present a rigorous evaluation of NPSV genotyping accuracy across multiple truth sets in the HG002 and NA12878 reference samples. We compare NPSV to similar standalone SV genotyping tools (that accept a VCF of putative SVs and aligned reads as input and predict the SV genotype), chosen to be representative of different alignment, graph and machine learning-based SV genotyping methods: Delly2[18], SVTyper[19], svviz2[20], Paragraph[25], GraphTyper2[21], ~~and~~ SV2[22] and GenomeSTRiP[26]. We show that NPSV consistently achieves similar or better genotyping accuracy across the different datasets, samples and variant types, can sensitively and specifically identify putative *de novo* SVs in a trio context and is robust to offsets in SV breakpoints.

Simulation-driven SV genotyping

The NPSV dataflow is shown in Figure 1~~Figure 1~~a. The inputs are the aligned reads (BAM/CRAM file), termed the “actual” data, and a VCF file of putative sequence-resolved deletion and insertion SVs. For each putative SV and possible genotype, NPSV generates synthetic short-read datasets using an NGS simulator configured to match the actual data (bottom path in Figure 1~~Figure 1~~a). We process the simulated datasets with the same alignment pipeline as the actual data and then extract re-alignment, read-pair and coverage SV features from each simulated replicate. The features extracted from the simulated data are used to train sample- and variant-specific classifier(s) to predict the genotype from the SV evidence similarly extracted from the actual sequencing reads. The simulation, feature extraction and classification

approaches are described in more detail in the Methods (NPSV Genotyping Algorithm) with the features specifically described in Table S1.

Figure 1b shows the simulated and actual SV evidence for an example homozygous alternate 822 bp deletion in the Genome in a Bottle (GIAB) HG002 call set[27], as would be generated to train a variant-specific classifier. The actual data is most consistent with the simulated homozygous alternate genotype. This SV is the deletion of one repeat of a tandem repeat. Due to the underlying repetitive sequence, no reads were uniquely ~~successfully~~ re-aligned to the SV's alternate allele and no alternate spanning fragments were identified. The simulated data shows that the absence of both of those features is consistent with the alternate allele for this SV (and pipeline) and is not an indication of a homozygous reference genotype as might otherwise be expected (indicated by the actual and simulated features in the first two panels "massing" on the y-axis). The NPSV variant-specific classifier correctly genotyped this variant as homozygous alternate, while genotypers that exclusively use realignment, split-read and/or spanning read evidence ~~alone~~ did not.

NPSV implements two genotyping approaches: a 1) "variant" model, like described above, which creates variant-specific classifiers trained on 100 replicates per variant per zygosity (i.e., 300*n* synthetic samples for *n* variants), and a 2) "single" model that creates a single sample-specific genome-wide classifier for each variant type (e.g., deletions, insertions) trained on one replicate per variant per zygosity (i.e., *3n* synthetic samples for *n* variants). The former approach is more computationally demanding but can be applied at any scale, including for just a single SV in a single sample. To reduce the computational burden, a "hybrid" model only builds variant- specific classifiers for smaller SVs (< 1 kbp by default) and uses the single model for

larger SVs. We generally observed the hybrid model to be most accurate for deletions and the single model to be most accurate for insertions and so set that as the default configuration.

Results

Genotyping accuracy

We evaluated NPSV and the comparison SV genotypers with multiple SV call sets across two samples: the GIAB version 0.6 call set for HG002[30], and the Polaris 2.0, Polaris 2.1 and SV-plaudit call sets for NA12878[31,32]. Genotype counts for each call set are shown in Supplemental Table S2. Using the call set SVs as the input, we report the genotype concordance, i.e., the fraction of predicted genotypes that exactly match the call set genotypes, and the non-reference concordance, which treats heterozygous and homozygous alternate genotypes as equivalent. The call sets and evaluation are described in more detail in the Methods.

Figure 2 shows the genotyping accuracy for NPSV and comparison tools for all truth sets. As a result of randomization in the simulations, SV sampling and classifier training, NPSV genotyping is not deterministic. In Figure 2 we show the mean accuracy for 10 complete NPSV genotyping runs and report the mean and standard deviation in Supplemental Tables S3-S6. Figure 2~~Figure 2~~a (Supplemental Table S3) shows the genotyping accuracy ~~for NPSV and comparison SV genotypers~~ for GIAB SVs in the high-confidence tier 1 regions (6449 DEL and 6462 INS SVs) and in the tier 1 regions combined with lower-confidence tier 2 SVs (8370 DEL and 8413 INS SVs). NPSV achieves similar or better exact genotype concordance and non-reference concordance than the comparison tools for both deletions and insertions. For SVs in tier 1 regions, NPSV improves genotype and non-reference concordance for deletions and insertions by 0.8~~1.1~~-2.14 percentage points. Figure 2~~Figure 2~~b (Supplemental Table S5) shows

the genotyping accuracy for the NA12878 truth sets (1143 DEL SVs in SV-plaudit, 8073 DEL and 6246 INS SVs in Polaris 2.0, and 20610 DEL and 12028 INS SVs in Polaris 2.1). NPSV generalizes across these datasets, achieving similar or better accuracy than the best comparison SV genotypers across all three datasets and both insertions and deletions.

Precision, recall and F1 scores for genotyping homozygous reference vs. non-reference SVs are shown in Table S4 and Table S6. We observed NPSV to achieve similar or better F1 scores than the comparison genotypers, albeit often with increased recall and reduced precision. For the Polaris 2.1 call set, which is enriched for homozygous reference calls and thus reflective of the common population genotyping use case, the mean DEL and INS recall are 0.939 and 0.985, respectively: the DEL and INS precision (false discovery rate) are 0.820 (0.180) and 0.945 (0.055). Given the small the variance across all metrics for NPSV, for concision, the remaining analyses utilize a single genotyping run.

Table S7 shows the genotype concordance for NPSV single and variant models for GIAB SVs in tier 1 regions grouped by SV length (SVLEN), the difference in length of reference and alternate alleles. Concordance generally increases with increasing SV length as read-pair and other features become more informative and a smaller fraction of variants overlap repetitive regions (see below). For deletions larger than 1 kbp, the single model showed increased accuracy. Those results motivated the default 1 kbp threshold for the hybrid approach, which uses the single model for larger SVs where that approach is more accurate and for which simulating an SV is more computationally demanding, and reserves the more computationally expensive but also potentially more accurate variant model for smaller variants. For GIAB insertions, the single model is more accurate than the variant model for all variant sizes. As noted above, based on these results, we set the NPSV default configuration to use the hybrid approach for deletions and

single model for insertions. Figure S3~~2~~ shows the genotype concordance for all call sets and tools grouped by SV length along with the underlying SV length distributions.

Due to the repetitive sequence, SVs in tandem repeats (TRs) are more difficult to accurately genotype. NPSV genotype concordance for GIAB DEL and INS SVs in tier 1 regions overlapping a TR > 100 bp (as annotated by GIAB) was 77.5% and 69.0~~3~~%, respectively, compared to 96.5~~8~~% and 92.1~~4~~% for DEL and INS SVs not overlapping a TR > 100 bp. Figure S4~~3~~ shows the genotype concordance for all NPSV modes for GIAB SVs in tier 1 regions grouped by SV length and whether the SV overlaps a TR > 100 bp. The [50,100) and [100, 300) size bins are enriched for SVs overlapping a TR > 100 bp, contributing to the reduced genotyping accuracy for these smaller SVs reported above.

To evaluate the use of the NPSV stand-alone genotyper with SVs identified with SV discovery tools (as opposed to benchmark call sets), we re-genotyped SVs called with Lumpy[33]/SVTyper[19] ~~(via smoove)~~ and Manta[34] in HG002. Using the discovery SVs as the input, Table 1 shows the genotyping accuracy and Table S8 shows precision, recall, and F1 scores ~~for the discovery SVs~~ compared to the GIAB SVs ~~genotypes~~ in tier 1 regions. To focus on genotyping accuracy, ~~in the discovery context~~ SVs that were not detected ("no-calls") were excluded from the concordance calculation. ~~NPSV achieves increased genotype and non-reference concordance for the discovery call sets compared to the genotypes predicted by the upstream SV callers.~~For the Lumpy call set the difference in genotyping accuracy is primarily driven by the count of putative false positives, which in turn is sensitive to the criteria for matching the call set to truth set SVs. Some of the putative false positive SVs may be true positive SVs (i.e., non-reference) that have sufficiently different breakpoints compared to the truth set SV so as not to match during concordance analysis. When the required size similarity is

relaxed from 70% to 30%, 150 more Lumpy SVs matched GIAB truth set SVs and the exact genotype concordance (non-reference concordance) for NPSV, 84.9% (91.3%), and Lumpy/SVTyper, 82.8% (91.9%), became more similar. The impact of offset or imprecise SV descriptions is described further below.

Trio analysis

We evaluated SV genotyping in a trio context using the HG002 trio. Table 2~~Table 2~~ shows the mendelian error rate (MER) and counts of different types of mendelian errors (MEs) for GIAB SVs in tier 1 regions. The NPSV MER for both deletions and insertions are greater than the MER for some of the existing genotypers, e.g., svviz2. However, most of the NPSV MEs are variants with low confidence genotypes and thus can be specifically filtered out based on the NPSV-reported ~~GQ (~~genotype quality (GQ); for example 94% of ME deletions in tier 1 regions~~. Most NPSV MEs~~ have a minimum GQ < 10 (among all trio members). ~~(over 93% for deletions), while the two most confident~~ The highest-quality NPSV deletion ME in the tier 1 regions ~~deletions are the two~~ was explicitly ~~likely or possible true positive *de novo* variants~~ reported by Zook *et al.*[30] as a “likely *de novo* deletion”. Supplemental Table S9~~8~~ lists the trio genotypes, minimum GQ and GQ ranking for that deletion and a second deletion ME ~~the two true-positive *de novo* deletions~~reported by *Zook et al.* in a locus known to undergo somatic rearrangement.~~ and two false positive insertions explicitly described by Zook et al. in the GIAB call set.~~ Consistent with Zook *et al.* NPSV genotyped the two deletions as *de novo*. ~~All were correctly called by NPSV.~~ NPSV in variant ~~and hybrid~~ mode~~s~~ reported the two deletions as the most confident ME ~~*de novo*~~ deletions in the tier 1 regions, and the hybrid mode reported the variants among the top 4 most confident~~with minimum GQ estimates of 99 and 15 respectively~~. ~~Svviz2 performed similarly; the reported *de* novo deletions were among the top 3 most confident MEs (i.e., the GQ threshold to~~

~~achieve 100% sensitivity for detecting the reported *de* novo deletions would result in one false positive ME).~~

Offset SV representations

As shown in Figure S4~~3~~, the set of GIAB SVs with discordant NPSV genotypes (i.e., the NPSV genotype does not match the GIAB genotype) is enriched for variants that overlap TRs. Across all NPSV modes, 86~~7~~+% of GIAB discordant deletions and 68~~9~~+% of discordant insertions in tier 1 regions are annotated in the GIAB call set as overlapping a TR > 100 bp, while less than 44% of concordant deletions and 30% of concordant insertions SVs are similarly annotated. Differences between the description of the putative SV (breakpoints and sequence change) and the true SV is one of the factors that contribute to genotyping errors for SVs in these repetitive regions (and more generally)[25]. We manually reviewed the pileup for 10 randomly selected deletions discordantly genotyped by NPSV in variant mode; 8/10 SVs were offset from the location indicated by long-read PacBio sequencing data.

To evaluate the impact of offset breakpoints more generally, we matched the GIAB SVs (PASS~~ing~~ variants only) in tier 1 regions to corresponding SVs called by PBSV[35] in PacBio long-read sequencing data (4114/4203 deletions and 5157/5443 insertions successfully matched). Making the assumption that the PacBio SV calls have correct breakpoints, we infer the offset from the distance between the GIAB breakpoints and the breakpoints identified in the long-read data (modeled on the approach in Chen et al.[25]). Figure 3 shows genotype concordance for SVs grouped by the breakpoint offset (the same analysis for select comparison tools is included in supplemental Figure S3**)**. For deletions, we observe an expected negative association between breakpoint offsets and genotyping accuracy; genotype concordance is 85+% for offsets up to 10 bp (and 95+% for no or single base offsets), decreasing to 49.6~~8+~~% for SVs with breakpoint

offsets greater than 50 bp. At larger offsets, the variant model increasingly outperforms the single model suggesting that the variant-specific classifiers may be~~are~~ better able to model the specific genomic context around offset deletions. For insertions we observe a similar negative association between breakpoint offsets and genotyping accuracy, although with a plateau for offsets of 1-20 bp. Much of the genotype concordance is recovered when using the long-read-derived SV calls as the input call set instead of the GIAB SVs (solid line in Figure 3).

The challenge of offset or imprecise breakpoints is further observed in the SV-plaudit call set. The majority of SV-plaudit variants greater than 1 kb (410/551) have imprecise breakpoints (including SVs with breakpoint confidence intervals of 100s or 1000s of bases). NPSV genotype concordance was 93.6% (132/141) for SVs with exact breakpoints, dropping to 63.7% (261/410) otherwise.

To investigate the potential for correcting SV descriptions using only the NGS data, we experimentally extended NPSV to propose and select among possible alternate alignments for an SV. We ~~would expect~~ hypothesize that the actual data is most similar to the simulated data for the correct SV description and genotype, and thus we could identify a better SV representation based on the distance between the actual and simulated SV evidence. For deletions of one or more copies of a TR, we proposed up to 10 different alignments of the deletion within the repetitive region, choosing the SV description where the real data is closest to the non-reference synthetic data. ~~Figure S5 describes~~ T~~t~~he SV proposal algorithm is described in more detail in the Supplemental Methods~~in more detail~~. When applied to the GIAB callset ~~While~~ SV proposal increases the sensitivity for calling heterozygous and homozygous alternate genotypes at the cost of smaller ~~, it~~ decreases in precision; the net effect is an ~~small~~ increase in genotyping accuracy (genotype concordance of 87.8% vs. 87.2% for SVs in tier 1 regions, 83.7% vs. 82.9% for SVs in

tier 1 and 2 regions) and F1 scores (0.94036 vs. 0.933 for SVs in tier 1 regions, 0.9264 vs. 0.914 for SVs in tier 1 and 2 regions) (Supplemental Table S109).

Computational requirements

The simulation process is computationally intensive, but also readily parallelizable. NPSV simulation and feature extraction are multi-threaded across variants. On a 36-core compute node (dual 18-core Intel Xeon 6140 2.3 GHz CPUs) genotyping 16,871 GIAB SVs required 21.1 hours (wall clock time as determined by the time utility); employing the single, variant and hybrid approaches exclusively required 53.3 minutes, 39.6 hours and 31.2 hours, respectively. Maximum memory resident set size was 22.1 GB as determined by the SLURM cluster manager (17.7 GB, 39.0 GB and 24.3 GB for the single, variant and hybrid modes). The NPSV preprocessing step, which generates sequencing statistics, e.g., insert size distribution, used in simulation and features extraction, is designed to use a combination of goleft[36], a fast alignment analysis tool, and metrics already calculated as part of a typical genome analysis pipeline, e.g. with the Picard tools[37]. In that approach, less than one minute is required for pre-processing. The currently un-optimized fallback preprocessor required 2.4 hours for the 25.5× HG002 BAM file. Table S10 lists execution time and resident set size for all of the comparison tools running on the same system.

Discussion

NPSV is a novel stand-alone SV genotyper that simulates putative SVs to train sample and variant-specific machine learning classifiers. NPSV consistently achieved similar or better genotyping accuracy than the comparison SV genotypers across both variant types and all truth sets (Figure 2Figure 2), including compared to the tools used to construct those truth sets (e.g.,

svviz2 for GIAB and Paragraph for Polaris). NPSV successfully and specifically identified the putative *de novo* SV deletions reported by GIAB. Improvements of 1-3 percentage points in genotyping accuracy translates to 10s-100s fewer incorrect genotypes per genome. Those incorrect genotypes leave cases unresolved, consume limited budgets for manual review and validation testing, and dilute downstream analyses.

SV call sets and reference databases can contain many SVs with incorrect or imprecise descriptions. For example, the clustering of SVs with similar but unique sequence changes during the construction of the GIAB call set reduced the number of SVs 2.3-fold[30], indicating many of the putative SVs did not have a single consensus description. Incorrect or imprecise SV descriptions can negatively impact genotyping accuracy[25]. NPSV maintains genotype concordances of 85+% (DEL) and 81~~2~~+% (INS) for offsets up to 10 bp; similar to or more robust than comparison tools (Figure 3, Figure S4).

Making the SV features even more robust to incorrect/imprecise SV descriptions could improve genotyping accuracy. However, in a strict interpretation of the precise sequence-resolved SVs in the GIAB call set, genotyping a putative SV with an incorrect description as non-reference would be inaccurate as that specific alternate allele is absent. Ideally, we would want to identify the correct SV descriptions as part of the genotyping process. We observed substantial increases in genotyping accuracy when using SVs called in long-read sequencing data as the input call set, suggesting there is an opportunity to further improve genotyping accuracy by refining the SV descriptions. We extended NPSV to select among alternative alignments for deletion SVs based on the similarity between the actual and simulated NGS data. The alternate representations increased the sensitivity for detecting non-reference genotypes with a net ~~, but with a corresponding decrease in specificity for homozygous reference genotypes (Table S9).~~ increase

in genotyping accuracy and F1 score for detecting non-reference genotypes compared to the original SV descriptions (Table S9). A substantial gap remains, though, between NPSV's NGS-only approach for refining SV descriptions and the accuracies observed when genotyping the long read-derived calls (Figure 3). We are actively working on improving all aspects (SV proposal, NGS simulation fidelity, features, and the similarity metric) of the SV refinement algorithm.

~~NPSV genotypes sequence-resolved deletion and insertion SVs.~~ Since NPSV simulates the expected alleles, it is limited to sequence-resolved SVs with discrete genotypes, and does not genotype "position independent" SVs, e.g., high-copy number duplications. At present, NPSV only supports biallelic deletions and insertions~~biallelic sites~~ and treats each SV independently. It does not currently genotype inversions or other SV types. However, the underlying method can be extended to support other SV types, e.g., inversions, and more complex variants/genotypes, e.g., compound heterozygous genotypes or multiple SVs on the same haplotypes. We hypothesize that the simulation-based approach, which is not dependent on the previous generation of representative training data, may be particularly useful for complex SVs. Minimal high-quality "ground truth" data is available for these sites; GIAB, for example, largely excluded complex SVs from the benchmark call set and most of the genotype errors identified in manual review were identified as complex[30].

The simulation process can be~~is~~ computationally intensive, particularly when simulating many replicates to build per-variant classifiers. ~~The time required for training sample- and variant-specific classifiers can be a limitation for NPSV. Genotyping 16,866 HG002 SVs using the default NPSV configuration required 21.0 hours on a 36-core server (exclusively using the single, variant and hybrid approaches required 55.4 minutes, 42.8 hours and 31.6 hours,~~

~~respectively, on the same system).~~ However, since the variant-specific classifiers can be built at the granularity of a single variant, th~~at more computationally demanding approach could~~ey can be employed in a targeted fashion, e.g., on SVs with low confidence genotypes or in repetitive regions, to model the biases introduced by the genomic region, sequencer and/or the analysis pipeline. Alternately when genotyping the same call set across multiple samples, the simulated training data could be reused. Preliminary experiments using the simulated training data generated for the parental samples to genotype the GIAB SVs in HG002 showed similar genotyping accuracy. When using shared training data, the simulation costs scale with the call set size, not the product of the call set and cohort sizes. Optimizing NPSV for large cohorts is an area of ongoing work. Large, highly consistent cohorts, such as gnomAD, can use other samples as the reference panel[38] but may have few and/or potentially ambiguous examples of extremely rare variants/genotypes. NPSV can effectively create a synthetic "reference panel" for all zygosities, for any variant, in any number of samples (including a single genome).

Conclusions

Here we present NPSV, a stand-alone SV genotyper for WGS data. Instead of attempting to develop a model for the complex and interconnected effects of the genomic region, sequencer and alignment pipeline on the observed SV evidence, NPSV employs detailed simulation of the sequencing process to train sample- and variant-specific classifiers for predicting SV genotypes. Since NPSV can generate relevant training data for any variant(s), at any granularity, it supports a range of targeted (a single variant) and large-scale (whole genome) SV genotyping applications. We showed that NPSV consistently achieves similar or improved genotyping accuracy for benchmark call sets. Looking forward, NPSV's simulation-based approach provides

a framework for genotyping the important “long tail” of SVs that are rare, complex and/or exclusively discovered with long-read technologies, and thus lack high-quality representative training examples.

Methods

NPSV Genotyping Algorithm

NPSV is a Python-based tool for stand-alone genotyping of sequence-resolved SV insertions and deletions. The inputs are the aligned reads (BAM/CRAM file), termed the “actual” data, and a VCF file of putative SVs. NPSV produces a copy of the input VCF with predicted SV genotypes.

Prior to genotyping, ~~NSPV preprocesses the aligned reads to~~ a preprocessing step estimates the mean, ~~and~~ per-chromosome and per-GC fraction coverage, and the insert size distribution ~~and GC bias~~. Those statistics inform the simulation and feature extraction. Many of those metrics are often already generated as part of the genome analysis pipeline and so do not need to be re-computed. In this evaluation, NPSV internally runs indexcov[36] and uses metrics previously computed with the Picard tools[37]. NPSV can also generate those statistics directly if needed using bedtools[39], SAMtools[40] and indexcov[36]. For each putative SV and possible genotype, NPSV generates one or more synthetic short-read datasets (termed replicates) using the ART NGS simulator[41] configured to model the actual sequencing data i.e., sequencer error model, read length, insert size distribution and coverage. In this evaluation we align the simulated WGS data with BWA-MEM[42] and mark duplicates with samblaster[43] to mimic the BCBio pipeline[44] used to align the actual data (along with SAMtools[40] and sambamba[45] for format conversion and sorting). The SV features extracted from the simulated

replicates (and randomly simulated regions in the genome, see below) are used to train sample- and variant-specific classifier(s). The SV features extracted from the actual data for putative SVs are only used to predict the genotypes (and not for training).

Features extracted from the simulation of the homozygous references genotype, i.e., the absence of the putative SV, can exhibit low variance, negatively impacting genotyping accuracy. To generate a more realistic "null" model, by default, NPSV generates the training data for homozygous reference genotypes by extracting features from the actual alignments for size-matched variants randomly sampled from the genome[46]. For haploid sex chromosomes, size matched variants are sampled from the sex chromosomes, otherwise variants are sampled from the autosome (and the X chromosome for SVs called on a diploid X chromosome).

NPSV extracts or derives the allele, spanning read and coverage SV features listed in Table S1 and described in more detail in the Supplemental Methods. NPSV counts the reference and alternate reads ~~determines the allele counts~~ by locally realigning read pairs to the reference and alternate sequences (derived from the putative SV description) using BWA[42] (via SeqLib[47]) and a read pair-aware alignment scoring metric adapted from svviz2[20]. Only reads originally aligned within some flanking distance (default of 99th percentile of the insert size) of the putative SV breakpoints are realigned. We extract insert size probability-weighted counts of s~~S~~panning reads (adapted from SVTyper[19]), counts of clipped reads (adapted from ~~and coverage features, adapted from the SVTyper[19],~~ SMRT-SV2[23]), and the mean event depth relative to flank regions, chromosome and regions of similar GC coverage features (DHFFC, DHFC, DHBFC adapted from ~~and~~ duphold[48]) ~~tools, are extracted~~ from the actual (original) alignments.

NPSV currently implements a Support Vector Machine (SVM) classifier for the single model and a random-forest (RF) classifier for the variant model using the scikit-learn framework[49].

The specific features used with each classifier are listed in Table S1. We observed this combination to achieve consistently high accuracy across variant types and call sets, although the differences in accuracy between classifier algorithms was typically small (1-1.5 percentage points). Data is centered and normalized to unit variance (using StandardScaler) prior to training the SVM (using a radial basis function kernel) with the same scaling used for genotyping. When training the single- SVM model, NPSV can perform a grid search of the C (1, 10, 100, 500, 1000, 5000, 10000) and gamma ("scale", 0.001, 0.0055, 0.01, 0.055, 0.1, 0.55) hyperparameters with 5-fold cross validation [23], however we did not observe consistently improved accuracy over default parameters for the GIAB call set and so disable the parameter sweep by default to reduce the training time. When training the single-model classifier, the training data is optionally filtered by genomic region. For the GIAB call set we excluded data outside the GIAB tier 1 regions. When training the per-variant classifiers, observations with features more than 5 standard deviations from the mean are excluded. To reduce execution time for the per-variant model, by default, we do not implement parameter sweeps during training. Evaluation of the RF-based variant model on the GIAB call set with different numbers of trees (10, 50, 100, 200) and maximum tree depth (variable, 3) indicated the default parameters (100 trees with variable depth) achieves high accuracy with reasonable execution time. The final genotypes and genotype quality (GQ) are determined from the label and class probabilities predicted by scikit-learn.

Truth Sets

We evaluated NPSV and the comparison SV genotypers with deletion and insertion SVs in the GIAB version 0.6 call set (GRCh37) for HG002, and the Polaris 2.0 (GRCh37), Polaris 2.1 (GRCh38) and SV-plaudit (GRCh37) call sets for NA12878. The truth sets were obtained from the GIAB FTP site[50], Polaris repository[31] and SV-Plaudit supplemental materials[51]. GIAB

SVs smaller than 50 bp or larger than 15 Mbp, SVs outside the GIAB tier 1 and 2 regions, SVs without genotypes and ~~no~~ filtered (i.e., not ~~n-~~PASS)~~ing~~ SVs, except for those variants filtered as "LongReadHomRef" (i.e., "long reads supported homozygous reference for all individuals"), were excluded. SV-plaudit and Polaris SVs smaller than 50 bp or larger than 15 Mbp, SVs without genotypes and ~~non-PASSing~~ filtered SVs were similarly excluded. In the SV-plaudit report[32] nine researchers manually inspected ~~the~~ SVs called in NA12878 by the 1000 Genomes Project[10]. The researchers were shown visualizations of data for the NA12878 trio and asked: "Does the sample support the variant type shown? […]", with the possible answers "True", "False", or "denovo". Only SVs for which more than 50% of the curators reported the sample supports the variant were retained. Almost all the curated SVs were deletions ~~(with the remainder inversions or duplications)~~, so we limited the SV-plaudit analysis to deletions. Supplemental Table S2 lists the counts of each genotype in the different truth sets.

Short-read Sequencing Data and SV Discovery

We genotyped the GIAB SVs in a subset of the NIST Illumina HiSeq 2500 2×148 PCR-free WGS data[52–55] with coverage representative of typical WGS (mean coverage of 25.5×, 20.4× and 24.7× for HG002, HG003 and HG004 respectively). We aligned the WGS reads to GRCh37 and performed point variant calling and SV discovery (using Lumpy[33]/SVTyper[19] via smoove[56] and Manta[34]) with version 1.0.9 ~~1.2.3~~ of the BCBio pipeline using the default BWA and GATK-based configuration[44]. We genotyped the NA12878 SVs in the Illumina Platinum Genomes 2×100 WGS data[57,58] (mean coverage of 50.5×). We aligned the NA12878 WGS reads to GRCh37 and GRCh38 with version 1.2.3 of the ~~the same~~ BCBio pipeline.

Comparison Tools

We compared NPSV to a representative set of stand-alone SV genotyping tools. The Delly2 (v0.8.3) genotyping module[18], ~~and~~ SVTyper (v0.7.1) [19] and GenomeSTRiP (v2.00.1958) [26] predict the genotype using a parameterized model incorporating multiple forms of evidence, e.g., depth, split-reads and read-pairs, extracted from original alignments. The svviz2 (commit b2c5126)[20] reporting module predicts the genotype assuming a binomial model for counts of reads realigned to the SV alleles with BWA. Paragraph (v2.4a)[25] and GraphTyper2 (v2.5.1)[21] employ a parametric model of reads realigned to a graph representation of the SV. SV2 (v1.5)[22] uses an SVM classifier trained on features extracted from 1000 Genomes data.

Unless otherwise noted, all tools were run with the truth~~call~~ set VCFs and BAMs produced by the BCBio pipeline as the inputs~~ and produced a genotyped VCF as an output~~. SV2, SVTyper and GenomeSTRiP do not support the insertion SVs in the GIAB and Polaris call sets and so were evaluated on the deletion SVs only. Prior to genotyping with Paragraph, we normalized the VCF to add a padding base for complex variants. The svviz2 genotypes were extracted from the "GT_mapq" field in the report to generate a genotyped VCF (we observed the "mapq" genotypes to generally be the most accurate for the GIAB call set). For SV2, variants called by GATK haplotype caller (as implemented in the BCBio pipeline) were used as the "SNV" input. For GraphTyper2, the GIAB tier 1 and 2 BED file was used to generate the regions for genotyping the GIAB HG002 call set, while the entire chromosomes were used as the regions for the NA12878 call sets; the "AGGREGATE" model was used as the output genotypes. GraphTyper2 converts insertions to duplications, those SVs were~~are~~ converted back to the call set representation to facilitate concordance analysis. Delly modifies the representation of some indel SVs such that the modified SV is no longer matched to the corresponding SV in the truth set

during evaluation, reducing the reported concordance by up to 0.3 percentage points. Each tool was run with its default parameters and thus the results presented here may not represent the best possible performance that could be achieved with expert tuning of the available configuration parameters. For example, GenomeSTRiP's high rate of "no-calls" (./.) for some smaller Polaris SVs can be impacted by the "minimum length to include depth-based genotype likelihoods" depth.effectiveLengthThreshold parameter (default of 200)[29]. The VCF FILTER annotations introduced by the Delly, GraphTyper, Paragraph and SV2 tools reduced genotyping accuracy (filtered genotypes are treated as "no calls" during concordance analysis) and so were ignored in all evaluations.

This evaluation does not exercise all of the capabilities of the different comparison tools, which may support other variant types, e.g., inversions, not yet implemented in NPSV, provide other features, such as visualization, or are explicitly designed for efficient population-scale genotyping as opposed to the single sample and trio analyses performed here.

Evaluation

We measured genotyping accuracy using Truvari[59], modified to report the genotype confusion matrix[60]. Figure S1a-b shows the definitions of concordance metrics calculated from the confusion matrix when using the "truth" SVs as the input to SV genotyping. Figure S1c-d shows the definition of the concordance metrics when using the output of an SV discovery tool as the input to the SV genotyper.

MEs were identified in autosomal regions using BCFTools[61]. We categorized MEs as a heterozygous or homozygous *de novo,* or other (e.g., homozygous alternate proband with a homozygous reference parent).

To evaluate the impact of imprecise breakpoints, we computed the genotype concordance for GIAB deletion SVs in tier 1 regions grouped by the maximum offset between the GIAB SV breakpoints and the corresponding SV breakpoints called in long-read sequencing data[25]. We used SV calls generated by PBSV 2.2.1 in PacBio CCS reads (obtained from the GIAB FTP repository). We matched the GIAB and PBSV calls with Truvari using the GIAB recommend configured to match SVs withination (2000 bp window, with 70% size and sequence similarity) [30] and extracted the offsets from the Truvari annotations.

Availability of source code and requirements

Project name: npsv

Project home page: https://github.com/mlinderm/npsv

Operating system(s): Linux

Programming language: Python, C++, BASH

License: MIT

RRID: SCR_020984

Availability of supporting data

The GIAB SV call set is available in the GIAB FTP repository, ftp://ftp-trace.ncbi.nlm.nih.gov/giab/ftp/release/AshkenazimTrio/HG002_NA24385_son/NIST_SV_v0.6 and the sequencing data at ftp://ftp-trace.ncbi.nlm.nih.gov/ReferenceSamples/giab/data//AshkenazimTrio/HG002_NA24385_son/NIST_HiSeq_HG002_Homogeneity-10953946/HG002_HiSeq300x_fastq/140528_D00360_0018_AH8VC6ADXX, ftp://ftp-trace.ncbi.nlm.nih.gov/ReferenceSamples/giab/data//AshkenazimTrio/HG003_NA24149_father/NIST_HiSeq_HG003_Homogeneity-12389378/HG003_HiSeq300x_fastq/140721_D00360_0044_AHA66RADXX, ftp://ftp-trace.ncbi.nlm.nih.gov/ReferenceSamples/giab/data//AshkenazimTrio/HG004_NA24143_mother/NIST_HiSeq_HG004_Homogeneity-14572558/HG004_HiSeq300x_fastq/140818_D00360_0046_AHA5R5ADXX for HG002, HG003 and HG004 respectively[30,52]

The SV-plaudit call set is available in the supplemental materials at http://gigadb.org/dataset/100450.[32] The Polaris call sets are available via GitHub, https://github.com/Illumina/Polaris. The NA12878 sequencing data is available in the European Nucleotide Archive under project PRJEB3381, ftp://ftp.sra.ebi.ac.uk/vol1/fastq/ERR194/ERR194147/ERR194147.fastq.gz.[57]

Declarations

Abbreviation

ME: Mendelian Error, MER: Mendelian Error Rate, NGS: Next-generation sequencing, RF: Random Forest, SV: Structural variant, SVM: Support vector machine, WGS: Whole genome sequencing

Ethics approval and consent to participate

Not applicable

Consent for publication

Not applicable

Competing interests

The authors declare that they have no competing interests

Funding

Research reported in this publication was supported by an Institutional Development Award (IDeA) from the NIGMS of the NIH under grant number P20GM103449, award UM1HL098123 from the NHLBI of the NIH, and the NSF under Grant No. 1827373. Its contents are solely the responsibility of the authors and do not necessarily represent the official views of NIGMS, NHLBI, NIH or the NSF.

Authors' contributions

MDL, AB and BDG conceived of the project. MDL, CP, MS and WK developed the software and performed the evaluation. MDL, AB and BDG wrote the manuscript. All authors read and approved the final manuscript.

Acknowledgements

References

1. Weischenfeldt J, Symmons O, Spitz F, Korbel JO. Phenotypic impact of genomic structural variation: insights from and for human disease. *Nat Rev Genet*. Nature Publishing Group; 2013; doi: 10.1038/nrg3373.

2. Brandler WM, Antaki D, Gujral M, Noor A, Rosanio G, Chapman TR, et al.. Frequency and Complexity of De Novo Structural Mutation in Autism. *Am J Hum Genet*. 2016; doi: 10.1016/j.ajhg.2016.02.018.

3. Kloosterman WP, Francioli LC, Hormozdiari F, Marschall T, Hehir-Kwa JY, Abdellaoui A, et al.. Characteristics of de novo structural changes in the human genome. *Genome Res*. Cold Spring Harbor Laboratory Press; 2015; doi: 10.1101/gr.185041.114.

4. Guan P, Sung W-K. Structural variation detection using next-generation sequencing data. *Methods*. 2016; doi: 10.1016/j.ymeth.2016.01.020.

5. Kosugi S, Momozawa Y, Liu X, Terao C, Kubo M, Kamatani Y. Comprehensive evaluation of structural variation detection algorithms for whole genome sequencing. *Genome Biol*. BioMed Central; 2019; doi: 10.1186/s13059-019-1720-5.

6. Mahmoud M, Gobet N, Cruz-Dávalos DI, Mounier N, Dessimoz C, Sedlazeck FJ. Structural variant calling: the long and the short of it. *Genome Biol*. 2019; doi: 10.1186/s13059-019-1828-7.

7. Chander V, Gibbs RA, Sedlazeck FJ. Evaluation of computational genotyping of structural variation for clinical diagnoses. *Gigascience*. Narnia; 2019; doi: 10.1093/gigascience/giz110.

8. Lappalainen I, Lopez J, Skipper L, Hefferon T, Spalding JD, Garner J, et al.. dbVar and DGVa: public archives for genomic structural variation. *Nucleic Acids Res*. Narnia; 2012; doi: 10.1093/nar/gks1213.

9. Alkan C, Coe BP, Eichler EE. Genome structural variation discovery and genotyping. *Nat Rev Genet*. Nature Publishing Group; 2011; doi: 10.1038/nrg2958.

10. Sudmant PH, Rausch T, Gardner EJ, Handsaker RE, Abyzov A, Huddleston J, et al.. An integrated map of structural variation in 2,504 human genomes. *Nature*. Nature Publishing Group; 2015; doi: 10.1038/nature15394.

11. Mills RE, Walter K, Stewart C, Handsaker RE, Chen K, Alkan C, et al.. Mapping copy number variation by population-scale genome sequencing. *Nature*. Nature Publishing Group, a division of Macmillan Publishers Limited. All Rights Reserved.; 2011; doi: 10.1038/nature09708.

12. Tattini L, D'Aurizio R, Magi A. Detection of Genomic Structural Variants from Next-Generation Sequencing Data. *Front Bioeng Biotechnol*. 2015; doi: 10.3389/fbioe.2015.00092.

13. Teo SM, Pawitan Y, Ku CS, Chia KS, Salim A. Statistical challenges associated with detecting copy number variations with next-generation sequencing. *Bioinformatics*. 2012; doi: 10.1093/bioinformatics/bts535.

14. Sedlazeck FJ, Rescheneder P, Smolka M, Fang H, Nattestad M, von Haeseler A, et al.. Accurate detection of complex structural variations using single-molecule sequencing. *Nat Methods*. Nature Publishing Group; 2018; doi: 10.1038/s41592-018-0001-7.

15. Huddleston J, Chaisson MJP, Steinberg KM, Warren W, Hoekzema K, Gordon D, et al.. Discovery and genotyping of structural variation from long-read haploid genome sequence data. *Genome Res*. 2017; doi: 10.1101/gr.214007.116.

16. English AC, Salerno WJ, Reid JG. PBHoney: identifying genomic variants via long-read discordance and interrupted mapping. *BMC Bioinformatics*. 2014; doi: 10.1186/1471-2105-15-180.

17. Goodwin S, McPherson JD, McCombie WR. Coming of age: ten years of next-generation sequencing technologies. *Nat Rev Genet*. 2016; doi: 10.1038/nrg.2016.49.

18. Rausch T, Zichner T, Schlattl A, Stütz AM, Benes V, Korbel JO. DELLY: structural variant discovery by integrated paired-end and split-read analysis. *Bioinformatics*. Bioinformatics; 2012; doi: 10.1093/bioinformatics/bts378.

19. Chiang C, Layer RM, Faust GG, Lindberg MR, Rose DB, Garrison EP, et al.. SpeedSeq: ultra-fast personal genome analysis and interpretation. *Nat Methods*. Nature Publishing Group; 2015; doi: 10.1038/nmeth.3505.

20. Spies N, Zook JM, Salit M, Sidow A. svviz: a read viewer for validating structural variants. *Bioinformatics*. 2015; doi: 10.1093/bioinformatics/btv478.

21. Eggertsson HP, Kristmundsdottir S, Beyter D, Jonsson H, Skuladottir A, Hardarson MT, et al.. GraphTyper2 enables population-scale genotyping of structural variation using pangenome graphs. *Nat Commun*. Nature Publishing Group; 2019; doi: 10.1038/s41467-019-13341-9.

22. Antaki D, Brandler WM, Sebat J. SV2: accurate structural variation genotyping and de novo mutation detection from whole genomes. Birol I, editor. *Bioinformatics*. Oxford University Press; 2018; doi: 10.1093/bioinformatics/btx813.

23. Audano PA, Sulovari A, Graves-Lindsay TA, Cantsilieris S, Sorensen M, Welch AE, et al.. Characterizing the Major Structural Variant Alleles of the Human Genome. *Cell*. 2019; doi: https://doi.org/10.1016/j.cell.2018.12.019.

24. Hickey G, Heller D, Monlong J, Sibbesen JA, Sirén J, Eizenga J, et al.. Genotyping structural variants in pangenome graphs using the vg toolkit. *Genome Biol*. BioMed Central; 2020; doi: 10.1186/s13059-020-1941-7.

25. Chen S, Krusche P, Dolzhenko E, Sherman RM, Petrovski R, Schlesinger F, et al.. Paragraph: a graph-based structural variant genotyper for short-read sequence data. *Genome Biol*. BioMed Central; 2019; doi: 10.1186/s13059-019-1909-7.

26. Handsaker RE, Van Doren V, Berman JR, Genovese G, Kashin S, Boettger LM, et al.. Large multiallelic copy number variations in humans. *Nat Genet*. Nature Publishing Group; 2015; doi: 10.1038/ng.3200.

27. Russell SJ, Norvig P, Davis E. Artificial Intelligence: A Modern Approach. Upper Saddle River, NJ: Prentice Hall;

28. Mohiyuddin M, Mu JC, Li J, Bani Asadi N, Gerstein MB, Abyzov A, et al.. MetaSV: an accurate and integrative structural-variant caller for next generation sequencing. *Bioinformatics*. 2015; doi: 10.1093/bioinformatics/btv204.

29. Chu C, Zhang J, Wu Y. GINDEL: accurate genotype calling of insertions and deletions from low coverage population sequence reads. *PLoS One*. PLoS One; 2014; doi: 10.1371/journal.pone.0113324.

30. Zook JM, Hansen NF, Olson ND, Chapman L, Mullikin JC, Xiao C, et al.. A robust benchmark for detection of germline large deletions and insertions. *Nat Biotechnol*. Nat Biotechnol; 2020; doi: 10.1038/s41587-020-0538-8.

31. : Polaris. https://github.com/Illumina/Polaris Accessed 2020 Jul 10.

32. Belyeu JR, Nicholas TJ, Pedersen BS, Sasani TA, Havrilla JM, Kravitz SN, et al.. SV-plaudit: A cloud-based framework for manually curating thousands of structural variants. *Gigascience*. 2018; doi: 10.1093/gigascience/giy064.

33. Layer RM, Chiang C, Quinlan AR, Hall IM. LUMPY: a probabilistic framework for structural variant discovery. *Genome Biol*. BioMed Central; 2014; doi: 10.1186/gb-2014-15-6-r84.

34. Chen X, Schulz-Trieglaff O, Shaw R, Barnes B, Schlesinger F, Källberg M, et al.. Manta: rapid detection of structural variants and indels for germline and cancer sequencing applications. *Bioinformatics*. Oxford Academic; 2016; doi: 10.1093/bioinformatics/btv710.

35. pbsv (2020). pbsv https://github.com/PacificBiosciences/pbsv

36. Pedersen BS, Collins RL, Talkowski ME, Quinlan AR. Indexcov: fast coverage quality control for whole-genome sequencing. *Gigascience*. Oxford University Press; 2017; doi: 10.1093/gigascience/gix090.

37Picard toolkit (2020). Picard Toolkit (Version 2.22.7) http://broadinstitute.github.io/picard/

38. Collins RL, Brand H, Karczewski KJ, Zhao X, Alföldi J, Francioli LC, et al.. An open resource of structural variation for medical and population genetics. *bioRxiv*. Cold Spring Harbor Laboratory; 2019; doi: 10.1101/578674.

39. Quinlan AR, Hall IM. BEDTools: a flexible suite of utilities for comparing genomic features. *Bioinformatics*. 2010; doi: 10.1093/bioinformatics/btq033.

40. Li H, Handsaker B, Wysoker A, Fennell T, Ruan J, Homer N, et al.. The Sequence Alignment/Map format and SAMtools. *Bioinformatics*. 2009; doi: 10.1093/bioinformatics/btp352.

41. Huang W, Li L, Myers JR, Marth GT. ART: a next-generation sequencing read simulator. *Bioinformatics*. Oxford University Press; 2012; doi: 10.1093/bioinformatics/btr708.

42. Li H. Aligning sequence reads, clone sequences and assembly contigs with BWA-MEM. 2013;

43. Faust GG, Hall IM. SAMBLASTER: fast duplicate marking and structural variant read extraction. *Bioinformatics*. 2014; doi: 10.1093/bioinformatics/btu314.

44. Chapman B, Kirchner R, Pantano L, Smet M De, Beltrame L, Khotiainsteva T, et al.. bcbio/bcbio-nextgen: v1.2.3. 2020; doi: 10.5281/ZENODO.3743344.

45. Tarasov A, Vilella AJ, Cuppen E, Nijman IJ, Prins P. Sambamba: fast processing of NGS alignment formats. *Bioinformatics*. 2015; doi: 10.1093/bioinformatics/btv098.

46. Parikh H, Mohiyuddin M, Lam HYK, Iyer H, Chen D, Pratt M, et al.. svclassify: a Method To Establish Benchmark Structural Variant Calls. *BMC Genomics*. 2016; doi: 10.1186/s12864-016-2366-2.

47. Wala J, Beroukhim R. SeqLib: a C ++ API for rapid BAM manipulation, sequence alignment and sequence assembly. *Bioinformatics*. Oxford Academic; 2016; doi: 10.1093/bioinformatics/btw741.

48. Pedersen BS, Quinlan AR. Duphold: scalable, depth-based annotation and curation of high-confidence structural variant calls. *Gigascience*. Oxford University Press; 2019; doi: 10.1093/gigascience/giz040.

49. Pedregosa F, Varoquaux G, Gramfort A, Michel V, Thirion B, Grisel O, et al.. Scikit-learn: Machine Learning in Python. *J Mach Learn Res*. 12:2825–302011;

50. : Genome in a Bottle Sequence-Resolved SV Calls v0.6. ftp://ftp-trace.ncbi.nlm.nih.gov/giab/ftp/release/AshkenazimTrio/HG002_NA24385_son/NIST_SV_v0.6 Accessed 2019 Jun 18.

51. : SV-plaudit Supplemental Data. https://oup.silverchair-cdn.com/oup/backfile/Content_public/Journal/gigascience/7/7/10.1093_gigascience_giy064/1/giy064_supp.zip Accessed 2018 Jul 24.

52. Zook JM, Catoe D, McDaniel J, Vang L, Spies N, Sidow A, et al.. Extensive sequencing of seven human genomes to characterize benchmark reference materials. *Sci Data*. Nature Publishing Group; 2016; doi: 10.1038/sdata.2016.25.

53. : Genome in a Bottle HG002 Sequencing Data. ftp://ftp-

trace.ncbi.nlm.nih.gov/ReferenceSamples/giab/data//AshkenazimTrio/HG002_NA24385_son/NIST_HiSeq_HG002_Homogeneity-10953946/HG002_HiSeq300x_fastq/140528_D00360_0018_AH8VC6ADX Accessed 2019 Jun 4.

54. : Genome in a Bottle HG003 Sequencing Data. ftp://ftp-trace.ncbi.nlm.nih.gov/ReferenceSamples/giab/data//AshkenazimTrio/HG003_NA24149_father/NIST_HiSeq_HG003_Homogeneity-12389378/HG003_HiSeq300x_fastq/140721_D00360_0044_AHA66RADXX Accessed 2019 Jun 4.

55. : Genome in a Bottle HG004 Sequencing Data. ftp://ftp-trace.ncbi.nlm.nih.gov/ReferenceSamples/giab/data//AshkenazimTrio/HG004_NA24143_mother/NIST_HiSeq_HG004_Homogeneity-14572558/HG004_HiSeq300x_fastq/140818_D00360_0046_AHA5R5ADXX Accessed 2019 Jun 4.

56. smoove (2020). smoove https://github.com/brentp/smoove

57. Eberle MA, Fritzilas E, Krusche P, Källberg M, Moore BL, Bekritsky MA, et al.. A reference data set of 5.4 million phased human variants validated by genetic inheritance from sequencing a three-generation 17-member pedigree. *Genome Res*. Cold Spring Harbor Laboratory Press; 2017; doi: 10.1101/gr.210500.116.

58. : Whole genome sequencing and variant calls for the Coriell CEPH/UTAH 1463 family to create a "platinum" standard comprehensive set for variant calling improvement. ftp://ftp.sra.ebi.ac.uk/vol1/fastq/ERR194/ERR194147/ERR194147.fastq.gz Accessed 2018 May 29.

59. Truvari (2020) Truvari https://github.com/spiralgenetics/truvari.

60. : Truvari fork. https://github.com/mlinderm/truvari/tree/genotype_stats

61. Danecek P, Bonfield JK, Liddle J, Marshall J, Ohan V, Pollard MO, et al.. Twelve years of SAMtools and BCFtools. *Gigascience*. Gigascience; 2021; doi: 10.1093/gigascience/giab008.

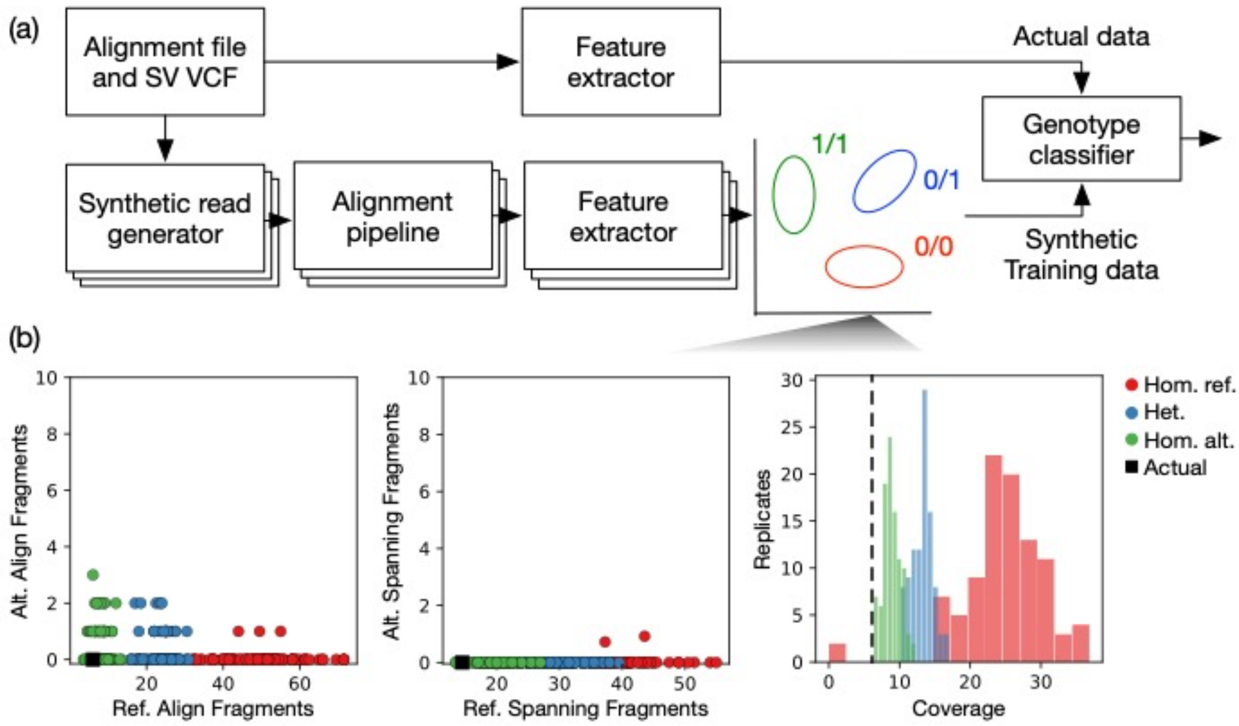

Figure 1: NPSV dataflow and example SV evidence. (a) NPSV dataflow showing the matched training and prediction pipelines. For each putative SV and genotype, NPSV generates one or more simulated replicates. This simulated data, shown in in the schematic as red, blue and green clusters for homozygous reference, heterozygous and homozygous alternate genotypes respectively, that are used to train sample- and variant-specific classifiers for predicting the SV genotype. (b) Synthetic training data (colored circles/bars) and actual data (black square/line) for a homozygous alternate 822 bp deletion in HG002. This SV is the deletion of one copy of a repeat and as a result of the repetitive genomic context, no fragments were uniquely re-aligned to the SV's alternate allele and no alternate spanning fragments were identified. The actual data is consistent with the simulated homozygous alternate data and not a homozygous reference genotype as might be expected from the absence of alternate allele re-andalignments. This SV is successfully genotyped as homozygous alternate by NPSV when building a variant-specific classifier.

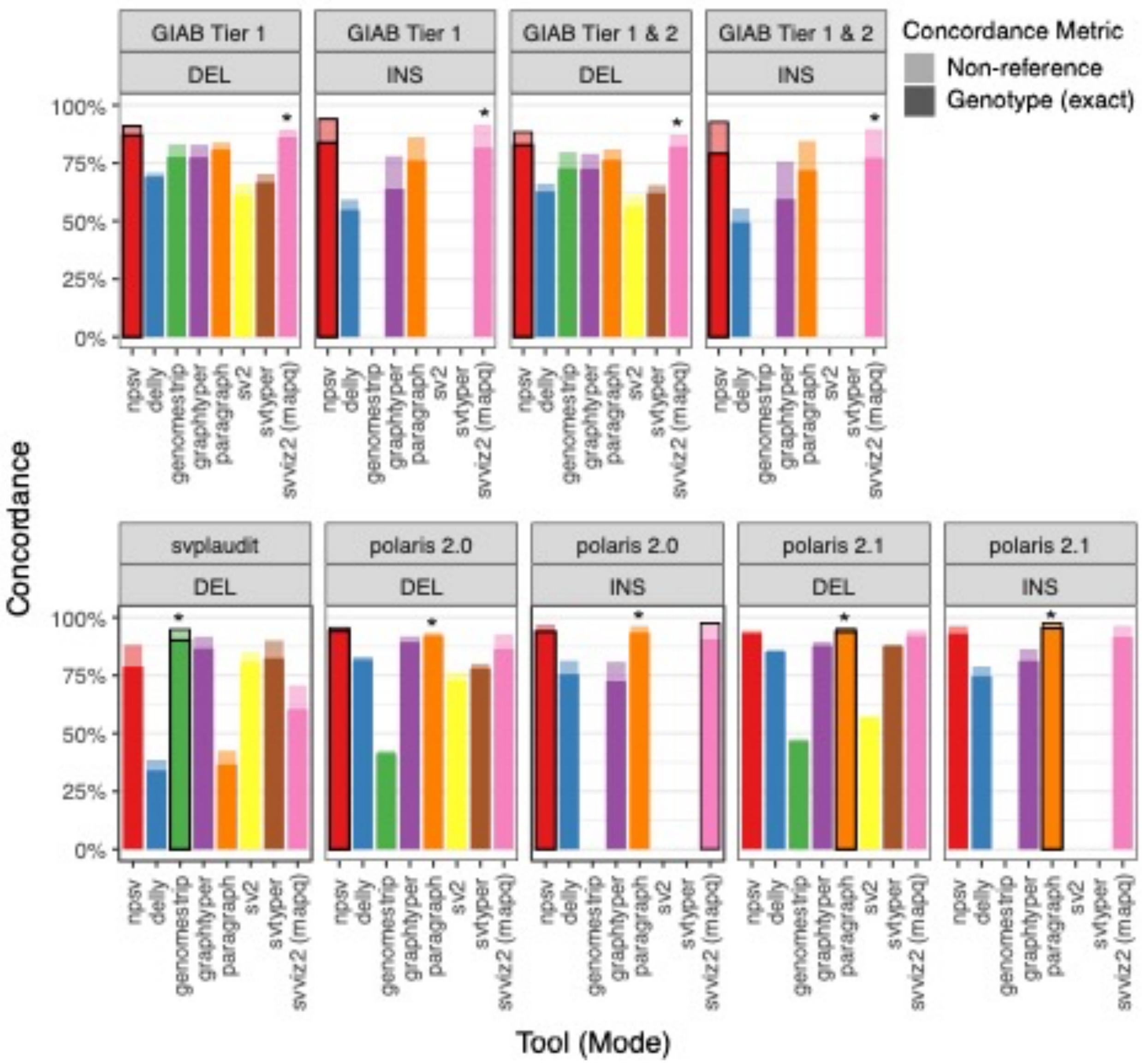

Figure 2: Genotyping accuracy for HG002 and NA12878 SVs. (a) Genotype concordance and non-reference concordance (presence or absence) for GIAB SVs (including "LongReadHomRef" SVs where "long reads supported homozygous reference for all individuals") in high-confidence tier 1 regions and the tier 1 regions and lower-confidence tier 2 SVs combined. (b) Concordance for NA12878 call sets. The NPSV accuracy is the mean of 10 runs. The best concordance is indicated with a black outline. The * shows tools used in the construction of that call set.

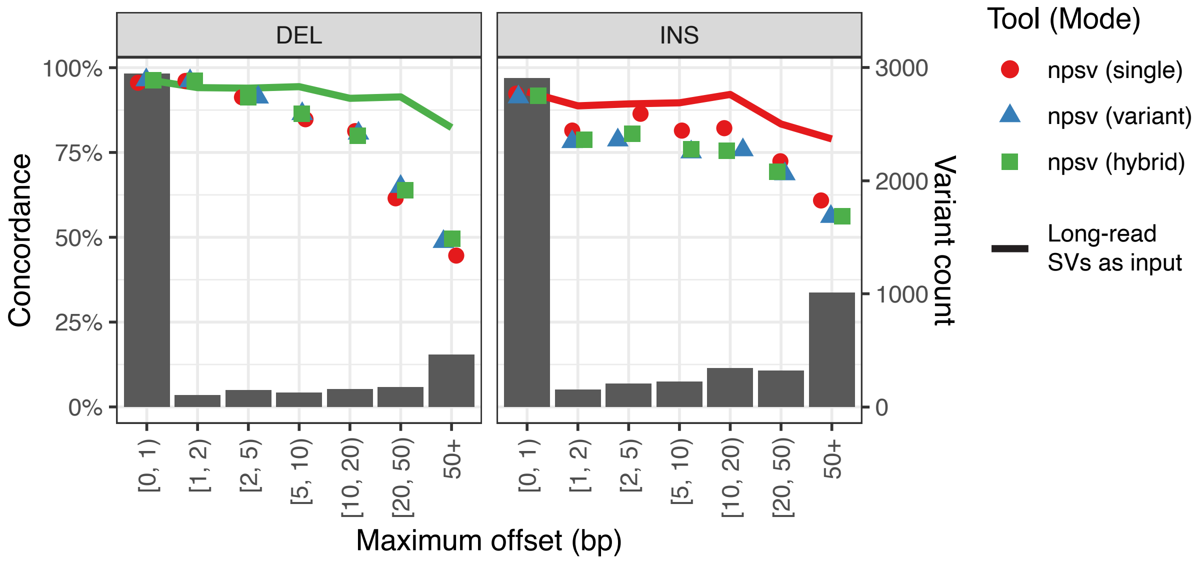

Figure 3: Genotype concordance for GIAB SVs with offset breakpoints. Genotype concordance for GIAB variant-only SVs in tier 1 regions grouped by the maximum offset between the GIAB breakpoints and the breakpoints for the corresponding SV called with PBSV in PacBio long-read sequencing data. The line shows the concordance when using the PBSV SVs as the input to NPSV running the default genotyoping mode ("hybrid" for deletions, "single" for insertions). The background bar chart shows the underlying distribution of offsets. The same analysis for select comparison tools is included in Supplemental Figure S54.

Table 1: Genotyping accuracy with discovery SVs as the input to SV genotyping and~~in~~ GIAB SVs in tier 1 regions as the truth set. Concordance is calculated for the subset of SVs successfully identified by the discovery tool.

			Caller Genotyping		NPSV Genotyper	
**Caller**	**Type**	**Discovery Recall**	**Concordance**	**Non-reference Concordance**	**Concordance**	**Non-reference Concordance**
lumpy	DEL	30.5~~5~~%	82.1~~3~~%	87.2~~0~~%	88.5~~8~~%	92.7~~5~~%
manta	DEL	67.9~~3~~%	90.1~~1~~%	91.8~~79~~%	92.2~~08~~%	93.6~~55~~%
manta	INS	25.2~~19~~%	87.3~~27~~%	93.54%	89.1~~8.96~~%	93.7~~66~~%

Table 2: Mendelian error rate (MER) and Mendelian error (ME) breakdown for GIAB autosomal SVs in tier 1 regions. NPSV default mode is shaded.

	DEL				INS			
**Tool**	**MER**	**Het. *de novo***	**Hom. *de novo***	**Other**	**MER**	**Het. *de novo***	**Hom. *de novo***	**Other**
npsv (single)	3.690% (23150/6416)	997	74	12549	4.645.36% (291(336/6269)	5865	69	22762
npsv (variant)	3.09% (198/6416)	111	1	86	5.14% (322/6269)	74	4	244
npsv (hybrid)	3.212.99% (206192/6416)	1063	12	9987	5.1025% (32029/6269)	657	66	24596
delly	1.66% (92/5535)	50	2	40	1.92% (78/4059)	26	0	52
genomestrip	2.02% (128/6337)	81	3	44				
graphtyper	5.53% (353/6386)	109	16	228	10.13% (608/6004)	86	27	495
paragraph	2.76% (175/6351)	85	2	88	5.42% (329/6067)	91	4	234
sv2	8.80% (536/6089)	129	47	360				
svtyper	2.28% (145/6349)	79	6	60				
svviz2 (mapq)	2.46% (158/6416)	94	3	61	3.32% (208/6269)	75	3	130

Figure 1

Click here to access/download;Figure;Figure1.pdf

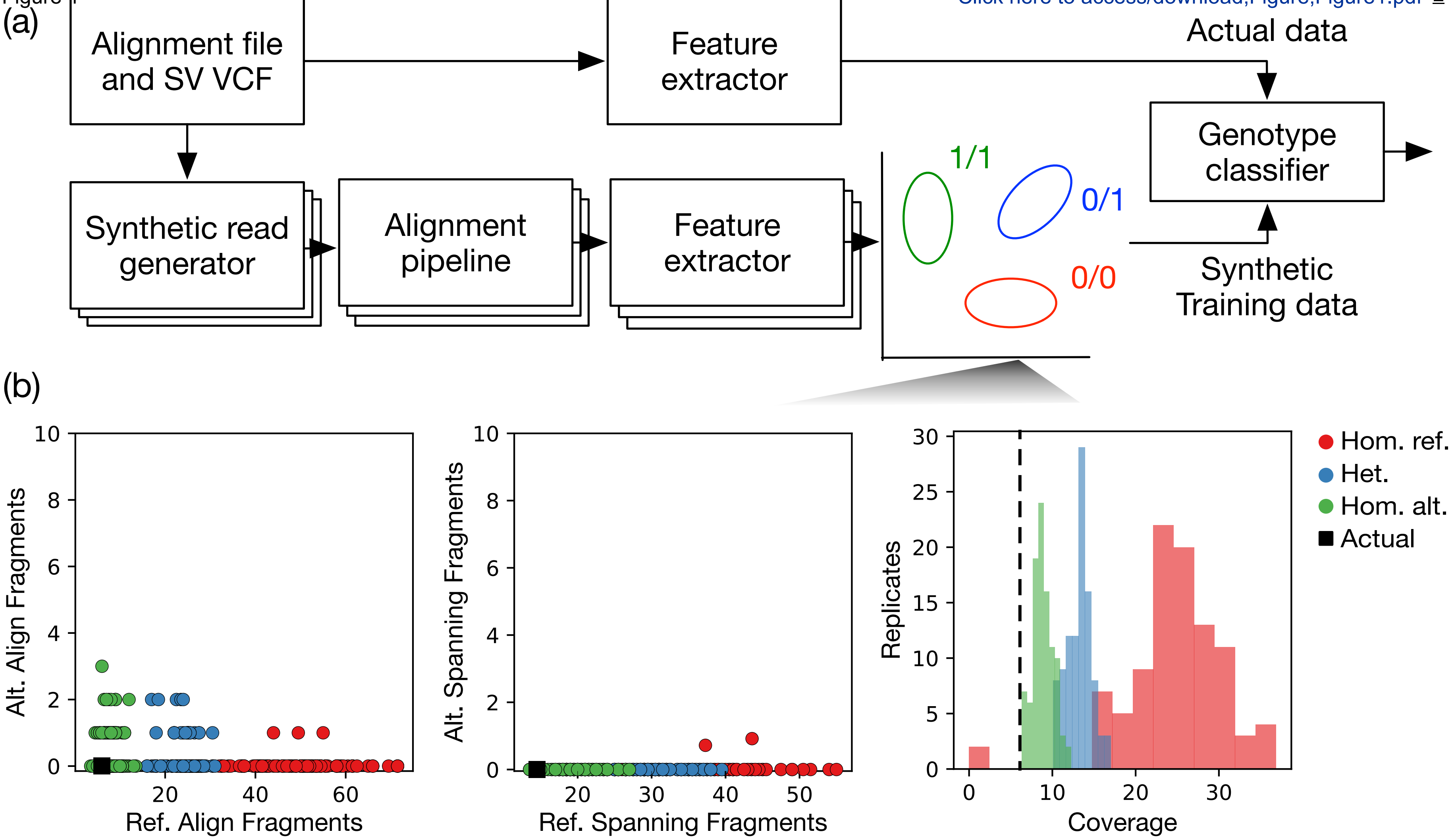

Figure 2

Click here to access/download;Figure;Figure2.pdf

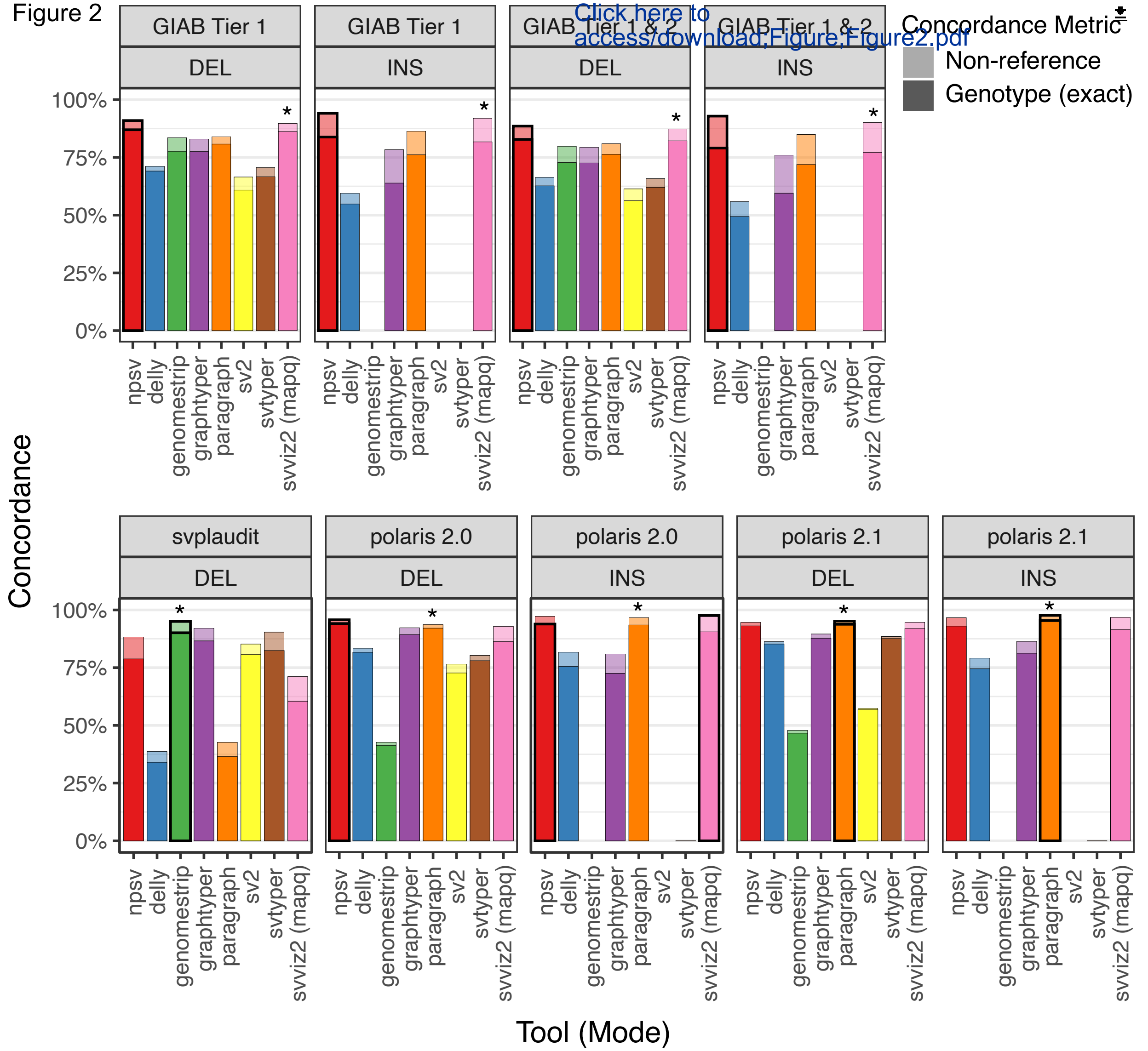

Figure 3

Click here to access/download;Figure;Figure3pdf.pdf

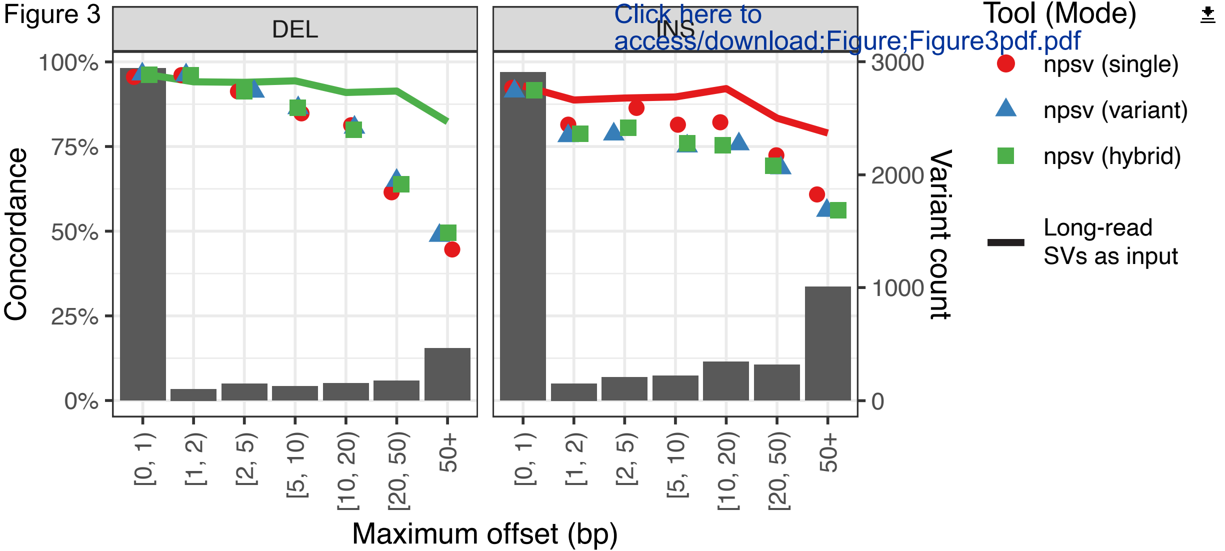

Click here to access/download
Supplementary Material
NPSVManuscriptSupplementalRevision1.pdf

Response to reviewers
Click here to access/download;Personal Cover;GigaScienceResponsetoReviewers1.pdf

Thank you to the reviewers for their thoughtful comments and feedback. Your feedback has significantly strengthened the manuscript. Please find the reviews below in *italics* and our point-by-point response in regular text.

The editor highlighted three main areas of revision in light of the reviews. We wanted to briefly summarize our revisions in those areas here in as an introduction, prior to the point-by-point responses that follow.

1) Reproducibility, also including documentation and instructions in your github repository.

We made the following revisions to the manuscript and additions/revisions to the GitHub repository:

- We incorporated an "end-to-end" example script into the repository for downloading and preparing the Genome in a Bottle data and then running the NPSV genotyper
- We added the benchmarking script we used to evaluate the run time for NPSV and comparison tools to the repository. This includes the commands used with each tool.
- We added additional documentation to the README including description of the tool arguments, the different workflows and links to the evaluation data
- We created a separate repository (https://github.com/mlinderm/npsv-commands) with the Makefiles we used to perform the evaluation
- We revised Figure 2 and the associated tables to show/report the mean and standard deviation for NPSV genotyping metrics across 10 end-to-end runs

2) Benchmarking / run times / validation, also including more tools in the comparisons.

We added a section on tool runtime to the results which includes runtime and memory usage for NPSV and the comparison tools. We have also added GenomeSTRiP to the set of comparison tools.

3) Some clarification with respect to aspects of the method.

We added additional details throughout the Methods section, with a particular focus on the classification approach. We have reorganized and expanded the Supplemental Methods section with a focus on describing the features used for classification and the workflow for proposing and selecting among alternate SV representations.

After the original submission, we identified a bias in the handling of homozygous reference training data. For efficiency, we evaluate the single and hybrid approaches by downsampling from a larger number of replicates. For homozygous reference replicates, this downsampling was biased towards the "beginning" of the genome instead of uniformly sampling from the entire genome. In comparing performance for GIAB SVs in tier 1 regions before and after correcting this bias, the difference in accuracy is typically 0.1-0.3 percentage points and not more than 0.7 percentage points. Those differences are not much larger than the standard deviation in accuracy we observe due to the inherent randomness in the simulation and classifier training (0.1 - 0.2 percentage points). We have rerun the single and hybrid model evaluations with the unbiased

downsampling and updated the results throughout. We also identified that Truvari was excluding approximately 200 variants in the Polaris 2.0 dataset during concordance analysis due to incorrect contig entries in the VCF header (in the original source file). We corrected the header and have updated the results throughout.

Since the original submission we have made algorithmic and other enhancements that have improved the performance of the SV “proposal” experiments. We have updated the manuscript with the current performance achieved in that mode.

Reviewer reports:

Reviewer #1: The authors present NPSV a tool to genotype structural variants. They evaulate it on several truthsets and show that it performs well across SV types and sizes. The analysis is well-done and this looks to be a nice improvement in the field.

My only major comments relate to 1) evaluation and 2) usage.

1. When genotyping large cohorts, a majority (>95% and more depending on size of cohort) variants genotyped in each will be homozygous reference. It's unclear how this would affect the final outcome. There is some hint of this in comparing the Polaris 2 vs

2.1 truthset where the latter has dramatically more hom-ref calls; in the 2.1, the performance of NPSV is always better in Polaris 2.

It could be that this information can be gleaned from the many tables, but as a potential user of this software, I'd like to be sure that the FDR will not be a problem when this is scaled to large call-sets.

The recall and precision (and thus FDR) for detecting non-reference genotypes (*i.e.*, heterozygous or homozygous alternate) were included in Supplemental Tables S4 and S6.

The large number of homozygous reference calls in Polaris 2.1 (and the contrast to the smaller number in the Polaris 2.0 GRCh37 call set) was one of the motivations to use that call set. As you point out, the large fraction of homozygous reference calls in Polaris 2.1 is reflective of the population genotyping use case. As shown in Supplemental Table S6, compared to Paragraph and svviz2, NPSV has higher recall but lower precision/higher FDR for detecting non-reference genotypes in the Polaris 2.1 call set (although ultimately similar F1 scores).

Table 1: FDR for genotyping homozygous reference vs. non-reference for the Polaris 2.1 dataset (adapted from Table S6). NPSV default mode is highlighted.

	**DEL FDR**	**INS FDR**
npsv (single)	0.218	0.055
npsv (variant)	0.176	0.036
npsv (hybrid)	0.180	0.036
paragraph	0.123	0.023
svviz2	0.129	0.026

We have added an additional paragraph in the results summarizing the precision, recall and FDR on the Polaris 2.1 call set in particular (as an example of the population genotyping use case).

*2. Again, related to large cohorts, what will the run-time be on huge cohort? The run-time should be noted more upfront as that is important; currently, it's only noted in passing in the discussion. It's also not clear the mechanics and total run-time for a cohort. Given a cohort of $n samples and $s SVs, is the simulation cost a one-time cost related to $s? or is it related to $n * $s? By this I mean can the simulation be done once per cohort or is it once per sample? It seems*

*it must be done once per sample ($n * $s); if so, I'd be interested to read about possible optimizations in the discussion that would mitigate run-time to be closer to $s.*

We have added a section to the results about the run-time and memory usage, including all of the comparison tools.

By default the simulation is O(n*s). This permits building models at any scale, including just a single variant in a single sample. However, it is possible to reuse the simulated training data across samples, *i.e.*, perform O(s) simulations. The features are designed to be relative with respect to coverage and so should be generalizable, to some extent, across different sequencing runs/samples (impact on accuracy is discussed more below). When reusing the training data, the computational time for the "first" sample, *i.e.*, to generate the training data, would be the same as the single sample times reported in the paper. The remaining samples can be genotyped much more quickly - on the order of 10s of minutes using a single core, since no simulation is being performed (we only need to extract features from the real data and predict the genotype).

Table 2 shows the concordance for GIAB Tier 1 SVs in HG002 when using simulations from different samples as the training data (for one run). We observe minimal degradation in accuracy when using the other sample in the HG002 trio for training and, in some cases, an improvement (although the differences are similar to the typical variation in accuracy that results from randomization in simulation, classifier training, *etc*.). In this case, the sequencing data is similar across all the samples (like we would expect in a single experiment), we might observe different results with more dissimilar data.

Table 2: Concordance for GIAB Tier 1 SVs in HG002 when using simulations from different samples as the training data. Analysis performed for a single run.

		DEL		INS	
	**Source**	**Genotype Concordance**	**Non-reference Concordance**	**Genotype Concordance**	**Non-reference Concordance**
npsv (single)	HG002	0.857	0.897	0.838	0.941
npsv (single)	HG003	0.857	0.894	0.845	0.944
npsv (single)	HG004	0.860	0.898	0.844	0.945
npsv (variant)	HG002	0.871	0.911	0.817	0.922
npsv (variant)	HG003	0.869	0.909	0.819	0.924
npsv (variant)	HG004	0.870	0.910	0.818	0.922
npsv (hybrid)	HG002	0.872	0.911	0.818	0.923
npsv (hybrid)	HG003	0.871	0.911	0.823	0.927
npsv (hybrid)	HG004	0.871	0.909	0.819	0.924

The above approach does not try to "pool" the samples during analysis. As we note in the manuscript, large highly consistent cohorts can use the other samples as the reference panel. Tools designed for population scale SV genotyping can potentially exploit multiple observations of the same SVs across the cohort in ways that NPSV does not. We initially focused on genotyping at the scale of a single variant and/or sample so that NPSV could be used in both targeted and genome scale contexts.

An alternate approach for reducing the simulation burden is to train a single model on a specific set of SVs, which is then reused across other samples and call sets. To investigate that approach, we genotyped HG002 GIAB Tier 1 and NA12878 Polaris 2.0 deletion SVs using the single model (*i.e.*, a single SVM) using the other sample and call set as the training data. Further, we did so using both the real and simulated data for training.

Table 3: Concordance for GIAB Tier 1 and Polaris 2.0 deletion SVs genotyped with the single model using different training data. Analysis performed for a single run.

**Training Data Source**		**GIAB Tier 1**		**Polaris 2.0**	
		**Genotype Concordance**	**Non-reference Concordance**	**Genotype Concordance**	**Non-reference Concordance**
GIAB Tier 1	Sim	0.857	0.897	0.936	0.953
	Real	0.881	0.916	0.935	0.950
Polaris 2.0	Sim	0.841	0.886	0.942	0.957
	Real	0.857	0.899	0.946	0.960

As we would expect, we observe the best accuracy when using a sample's real data as the training data (with 5-fold cross validation). For these call sets and samples, we observe that using simulated data generated for a specific sample for training (i.e., based on that sample's SVs, coverage, read length, insert size distribution, etc.) produces similar or better accuracy than using the other sample's real or simulated data for training.

We incorporated a brief summary of these results into the discussion section. Our initial focus was on genotyping single variants/samples. Optimizing and adapting NPSV for larger cohorts is an area of ongoing work.

Minor Comments

==============

+ what is purpose of extending y-axis to 60 or 70 in the scatter plots in Fig 1b? Can the y-axis be set to < 10?

+ difficult to see the black square (actual SV) in Fig 1b (this might be fixed by limiting y-axis).

The intent was to show the asymmetry of ref. and alt. alleles, even when the simulated variant is heterozygous. However, since multiple reviewers commented on that choice, it is clearly more

confusing than illuminating. We have adjusted the axes and increased the size of the black square.

+ in figure legend for 1b. can you explain why there is no apparent alt support?

We added a brief explanation of how the repetitive genomic context impacts the observed re-alignments. The SV is the deletion of one copy of a repeat that is sufficiently long such that most (or all) of the “alternate” reads realign to the reference and alternate alleles with similar scores (and thus are considered not uniquely mapped and not assigned to a specific allele).

+ since you are using features derived in duphold, it would be interesting to compare calls filtered as recommended by duphold (DHFFC < 0.7) when comparing to NPSV. (this is not a requirement for my review, but potentially of interest)

In response to this comment, we ran duphold on the GIAB “tier 1” deletion SVs using the suggested DHFFC filter. Treating GIAB genotypes as the truth set, duphold excluded 73.8% (1656/2245) of the homozygous reference (i.e., LongReadHomRef) SVs and also 18.7% (786/4204) of heterozygous and homozygous alternate SVs. In comparison, NPSV correctly genotyped 85.5% (1920/2245) of the GIAB homozygous reference calls as homozygous reference and incorrectly genotyped 5.88% (247/4204) of the GIAB heterozygous and homozygous alternate SVs as homozygous reference. Given that duphold and NPSV solve different problems, for concision and consistency, we elected not to include the above comparison data in the manuscript.

More generally, these results are another example of the possibility for more precisely targeted ensemble approaches. In the best case, we would only employ the more expensive simulation-based genotyping when it would be uniquely beneficial. Precisely identifying those SVs is an area of ongoing work.

+ as the author of duphold and creator of the DHFC metrics it would be nice to see those metrics explicitly noted in the main text.

We updated the manuscript Methods section to explicitly name DHFFC, DHBFC and DHFC alongside the existing citation to duphold.

Software

========

It's not clear from the github how to run the software end-to-end. If picard metrics are not present, then one can run `npsvg preprocess` without those arguments? Then run `npsv --stats-path $json ...`?

Yes. The Picard metrics are a “short cut”. If pre-computed metrics are not available, the preprocess command will compute the coverage and insert size metrics directly. We have updated the README to make the end-to-end workflow more clear and incorporated an example script into the repository. We also updated the Methods section in the manuscript with more details about the different preprocessing approaches. We realized the underlying tools used in the preprocessing step were not cited in the manuscript. We have added those citations.

How does the `propose` fit in and `refine`?

Those two commands implement the experimental functionality for selecting alternate SV representations based on the simulated data. The *propose* command generates alternate possible

representations for an SV. That expanded set of SVs is genotyped by NPSV using the variant mode. The *refine* command reduces the expanded set of SVs back to the original SVs, updating the genotypes as appropriate if a more similar SV-genotype combination is observed. We have expanded the description of those pre- and post-processing tools in the Supplemental Methods section.

A single set of commands layed out end-to-end would be helpful.

We have included an "end-to-end" example script and a benchmarking script with the commands for all tools in the repository. We have also updated the README with more guidance on how to run the tool.

Reviewer #2: Author's present NPSV, a del/dup genotyping method that uses simulated data to set build a training set and uses machine learning methods to genotype SVs. This is an important problem.

The manuscript is written in acceptable language but needs to be checked for grammar and typos. Authors make interesting points but they could be presented better. While the results look convincing, but the study is missing key details (details of the methodology) and comparisons that need to be added.

I added my comments below, which I hope will make the study and the presentation to improve:

0. Firstly, I would like to thank authors for making a very detailed and comprehensive github page. Just to make it a little more complete, please add a small section to download the publicly available datasets that are used in the manuscript. Please also include the command line options to regenerate the results in the paper for each result.

We have updated README with additional links to the source data and more comprehensive usage examples (links to the source data were included in the manuscript's Data Availability section). We incorporated the script used for runtime benchmarking the tools into the repository itself to show how we ran all of the different tools and created an example script in the repository that downloads, prepares and genotypes the GIAB SVs. The results in the paper were generated with a series of Makefiles. We have created a separate public repository (https://github.com/mlinderm/npsv-commands) with those files.

1. Please have the manuscript grammar checked. While it reads generally well, there are numerous typos and grammatical mistakes.

As part of this revision, we have carefully copy-edited the manuscript.

2. NPSV is a genotyping tool and does not explicitly detect SVs, which is also a very complex problem. I think this distinction should be made clear early in the manuscript. I would even recommend adding this to the name of the method, i.e. changing it to NPSVG. This distinction becomes important when NPSV is compared to other methods such as Delly which are primarily designed for joint detection+genotyping of SVs.

We fully agree about the critical distinction between SV discovery and SV genotyping. In the very first paragraph, we note that we are exclusively focused on stand-alone genotyping: "While numerous tools integrate SV discovery and genotyping, our focus here is "stand-alone" genotyping of putative SVs identified by discovery tools and/or obtained from the literature/SV catalogs." To further distinguish NPSV from tools that perform both discovery and genotyping, we updated the abstract to describe NPSV as a "a machine learning-based approach for genotyping previously discovered SVs that employs NGS simulation…".

3. The authors should describe the limitations and strengths of each tool, for instance graphtyper2 is designed for population scale genotyping, and svviz is a visualization tool but it performs fairly well in comparisons.

In the Methods section "Comparison Tools", we had included a brief descriptor of the underlying approach of each tool. We have expanded that section with a paragraph noting the limitations of this evaluation as it relates to the comparison tools:

"This evaluation does not exercise all of the capabilities of the different comparison tools, which may support other variant types, *e.g.*, inversions, not yet implemented in NPSV, provide other

features, such as visualization, or are explicitly designed for efficient population-scale genotyping as opposed to the single sample and trio analyses performed here."

This manuscript is focused on the NPSV method; it is not intended to be a comprehensive comparison of SV genotypers, like the Chander *et al*. paper cited in the introduction (along with the Kosugi *et al*. evaluation of SV discovery tools). We feel that such a comparison is beyond those scope of this work. This evaluation is focused on relevant, but specific, use cases that do not include all variant types or potential kinds and scale of NGS data (*e.g*., large cohorts or low-coverage data). As such, we hesitate to discuss potential strengths or limitations that we did not specifically evaluate.

4. Fig 1b. How is this figure representing the simulated data? Why is the scale of y-axis (of the alternate allele fragments) is very large? It makes it weird and hard to view the data. Also the actual data points that are supposedly colored in black are not clearly visible.

The intent was to show the unexpected allele balance (*e.g*., there are not similar numbers of ref. and alt. fragments for heterozygous genotypes). However, since multiple reviewers commented on that choice, it is clearly more confusing than illuminating. We have adjusted the axes and made the real data (black square) larger to be more visible.

5. In Figure 1a, I find the coordinate system and the ellipses very vague, i do not understand what they represent.

The ellipses are intended to represent the clusters of simulated data, similar to what is shown for the actual data in Figure 1b. We updated the caption to better describe that aspect of the figure.

6. Most of the results are for Ins/Dels but methods is allegedly for SVs? Translocations, inversions, etc? This is important again because other methods like Delly is tuned to work on complex rearrangements, i.e., that is one of their main strentgth.

We note in the beginning of the methods that NPSV currently genotypes sequence-resolved deletions and insertions. At present, the NPSV implementation does also support DUP SVs that are effectively a sub-type of insertions. However, since the truth sets used in this evaluation contain few or no explicit DUP SVs of that type we think of that as an experimental feature. Currently NPSV does not support other SV types. Implementing and testing additional variant types is an area of ongoing work. We have further noted that focus/limitation in the beginning of the Results and Discussion sections.

7. How is the simulation strategy different from other simualtion tools? Authors need to make benchmarks of the simulation tools with reasonable comparison metrics.

We are not sure what comparison is intended here. NPSV is not intended to be used as an SV simulator, such as those designed to generate random SVs for testing SV discovery tools. NPSV uses the ART NGS simulator as an internal component to generate the synthetic training data. While simulation fidelity is important for NPSV, the ultimate metric of interest is SV genotyping accuracy.

8. Hyperparameters of the simulation strategy, how is ART simulator's parameters selected? These selections must be justified. What are the parameters of NPSV's simulation strategy that impacts the accuracy?

We use the ART simulator's pre-generated sequencer profiles (error models) for the relevant sequencer model, *e.g.*, HiSeq 2500 (HS25), and set the read length, coverage and insert size distribution to mimic the sample being genotyped. The simulated haplotypes are determined by the putative SV being genotyped. We have experimented with modeling GC bias (implemented by filtering the ART output based on fragment GC content and normalized GC coverage of the sample being genotyped) but did not observe a consistent improvement in genotyping accuracy. We have also experimented with other NGS simulators (*e.g.*, NEAT, insilicoseq, ReSeq, SimuSCoP), but either had trouble running the tools in our workflow (which performs thousands of simulations of small regions) or did not observe increased genotyping accuracy.

9. AF of the SV vs the accuracy of genotyping? This can be simulated on a subsample without the SVs and remaining samples can be tested.

We are interpreting "AF" here as population allele frequency, and the question as asking how the number of observations of an SV in a cohort impacts NPSV genotyping accuracy. If we interpreted the question correctly, the AF does not have any impact on NPSV genotyping accuracy. Since each sample is genotyped independently and the data are not "pooled", the population frequency does not impact accuracy.

10. While dels and dups are separated, dups are more complex then dels: Dups can have very high copy numbers while dels can take 3 values. Very high and very low CNs? These are really the hardest CNs to genotype.

As we note in the manuscript, NPSV is designed to genotype position-specific sequence-resolved variants for which we can simulate the putative alternate genome (and the genotype can have one of three values). We interpret this comment as referring to "position independent" duplications that cannot be readily simulated in the same way. From our perspective, genotyping that type of SV is a distinct problem, and not one that NPSV is designed for. We have further emphasized that limitation in the discussion section, noting "Since NPSV simulates the expected alleles, it is limited to sequence resolved SVs with discrete genotypes, and does not genotype "position independent" SVs, *e.g.*, high-copy number duplications."

Our development and evaluation focused on the DEL and INS variants included in the GIAB and Polaris call sets. We know, however, that some of the INS variants in the GIAB call set could be described as duplications (Pederson et al. [1] report that 805 of the insertions in GIAB call set are effectively duplications). Thus this evaluation does include duplications, albeit those that are "sub-type" of position-specific insertions. Explicitly converting those insertions to duplications, where relevant, could enable NPSV to better utilize coverage features; doing so is an area of ongoing work and evaluation.

[1] Brent S Pedersen, Aaron R Quinlan, Duphold: scalable, depth-based annotation and curation of high-confidence structural variant calls, GigaScience, Volume 8, Issue 4, April 2019, giz040, https://doi.org/10.1093/gigascience/giz040

11. It would be useful to summarize the number of SVs used in benchmarks (around line 115) and SV allele frequency distributions.

We added the DEL and INS SV counts for each dataset to that paragraph.

12. I am surprised that the authors did not make use of 1000 Genomes call sets, is there a specific reason for this? 1kG used a complex wet lab validation process to estimate accuracy of the variant calls. I would think this is the best dataset to use.

The SV-plaudit call set is extracted from the 1000 Genomes (1kG) calls in NA12878. The program record in the SV-plaudit VCF header indicates it was constructed by selecting calls with at least one alternate allele in NA12878 from the 1kg SV callset (NA12878 ALL.wgs.mergedSV.v8.20130502.svs.genotypes.vcf.gz). We modified the description of the SV-plaudit call set to note the upstream source of the SVs: "In the SV-plaudit report[30] nine researchers manually inspected SVs called in NA12878 by the 1000 Genomes Project[10]".

More generally, we sought to find benchmark sets with sequence-resolved, multi-technology calls, especially long-read calls (to mimic the common use case of genotyping SVs in NGS data first discovered in long-read calls), with full zygosity information (all three genotypes). We further looked for call sets that would allow us to reuse samples, specifically NA12878, while varying the reference genome and genotype balance, and incorporating manual curation.

We note that Human Genome Structural Variation Consortium (HGSVC) recently released a new set of calls in the 1kG samples[1]. However, the unified multi-technology VCF linked from that paper (available at dbVar nstd152) does not seem to have full zygosity information, just the presence or absence of the variant allele.

[1] Chaisson, M.J.P., Sanders, A.D., Zhao, X. et al. Multi-platform discovery of haplotype-resolved structural variation in human genomes. Nat Commun 10, 1784 (2019). https://doi.org/10.1038/s41467-018-08148-z

13. Following previous comment, i am confused by HG002 analysis which states that the SVs are called by Lumpy/SVTyper and Manta. Where does the ground truth come from in these experiments? Are they validated?

We are interpreting this question as referring to Table 1. That experiment used SVs called by Lumpy and Manta (as implemented in the BCB pipeline we used to align the data) as the input to the NPSV genotyper instead of using the GIAB call set as the input. The ground truth is still the GIAB call set and genotypes used throughout. The intent was to show the genotyping accuracy for SVs produced by representative discovery tools. We modified the caption to specify that the GIAB call set was used as the truth set and added a table to the supplemental with the Recall/Precision/F1 for those call sets to provide the same metrics we do when using the truth set as the genotyping input.

14. Is there a specific set of dels/dups that NPSV fails?

As we noted in the paper, we observed diminished performance for offset/imprecise SVs and particularly for larger offset/imprecise variants (> 1 kbp). In Supplemental Figure S2, we observe that the SV-plaudit/1000 Genomes calls are enriched for larger SVs (compared to the other call sets) and that NPSV accuracy decreases (especially relative to SVTyper, GraphTyper, SV2 and GenomeStrip) for those larger variants.

The figure below shows counts of SVs (dis)concordantly genotyped by NPSV in the default mode (hybrid) by maximum confidence interval for different SV size bins. A larger fraction of the SVs with SVLEN > 1 kbp have imprecise breakpoints, and particularly confidence intervals (width of VCF CIPOS and CIEND fields) of 100s or even 1000s of bases. Consistent with the discussion of offset variants in the manuscript, NPSV accuracy degrades for the larger imprecise

variants. At present NPSV does not utilize the confidence interval information. Doing so is an aspect of ongoing work and could potentially improve accuracy for some of these particular variants.

We observed similar trends when genotyping the Lumpy-discovered SVs. For the set of true positive SVs genotyped as such by Lumpy/SVTyper but not NPSV, none of the breakpoints exactly match the GIAB SV and 59% were greater than 1 kb size. Manual review of 20 random Lumpy/SVTyper putative false positive SVs indicated half were likely true positive calls, but with sufficiently different breakpoints that Truvari did not match the Lumpy SV to the GIAB SV.

We added further detail about genotyping the Lumpy call set to result section on genotyping discovery call sets and a description of the observations for larger imprecise SVs in SV-plaudit to the "Offset SV representations" section of the results.

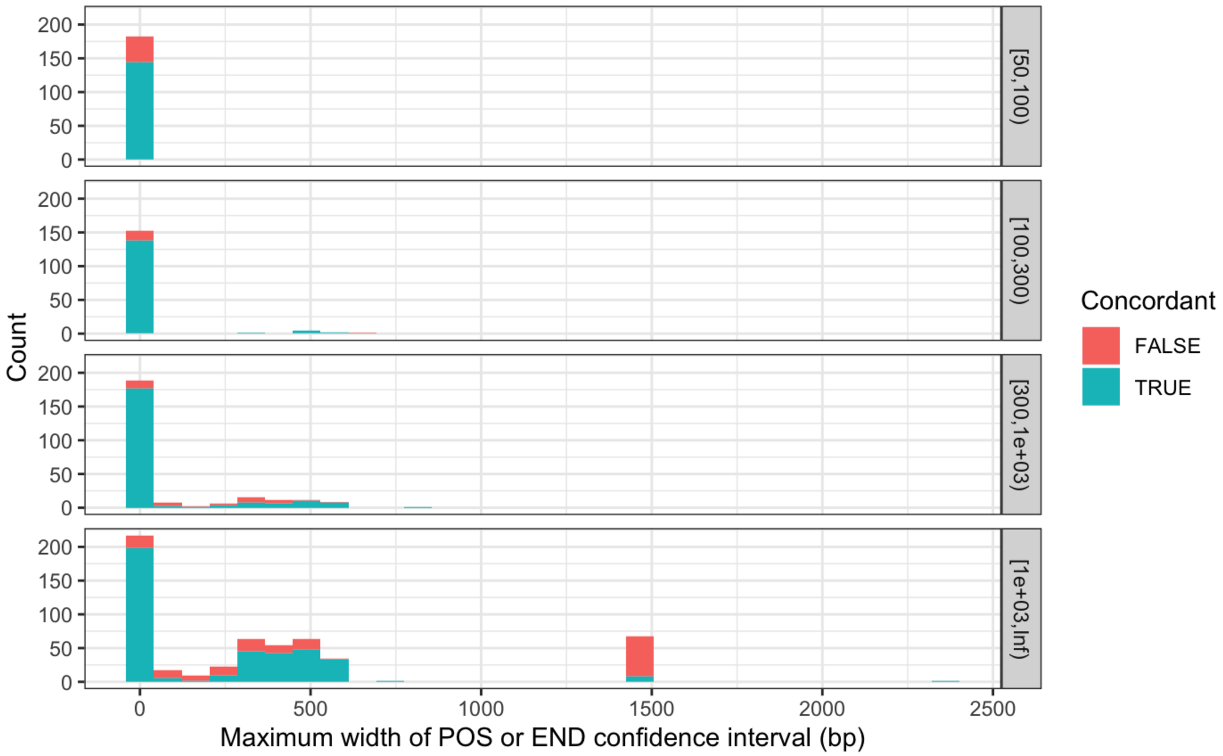

Figure 1: Histogram of genotype concordance for NPSV hybrid mode for SV-plaudit SVs by maximum width of breakpoint confidence intervals (CIPOS, CIEND)

15. *The authors should add the SV calling and genotyping methods used in the 1000 Genomes project such as CNVnator, GenomeStrip and also VariationHunter and BreakDancer (these may not be applicable) in the comparisons.*

We have implemented GenomeStrip (genotyping component-only) as a comparison tool for deletions (it does not appear to support the sequence resolved insertions included in the GIAB and Polaris truth sets).

Previous studies[1] indicate that CNVnator "less effectively detected small DELs and/or DUPs.", and specifically does not appear to effectively identify variants smaller than 1 kbp (termed DEL-

S by the authors of [1]). Since the majority of GIAB and Polaris SVs are less than 1 kbp in size, CNVnator seems inappropriate as a comparison tool for those datasets.

Tardis (which looks to have replaced VariationHunter) and BreakDancer appear to be SV discovery tools focused on presence or absence of SVs, instead of genotyping. Since we are focused on genotyping previously discovered SVs (as opposed to discovery), we do not think that incorporating those comparison tools will enhance the evaluation.

[1] Kosugi, S., Momozawa, Y., Liu, X. et al. Comprehensive evaluation of structural variation detection algorithms for whole genome sequencing. Genome Biol 20, 117 (2019). https://doi.org/10.1186/s13059-019-1720-5

16. It is necessary to include a time/memory benchmarking on a reasonable sized set of SVs.

We previously included computation time for over 16,000 SVs in HG002 in the discussion. We have expanded upon that benchmarking in the Results section and included the runtime and memory usage for all tools in the supplement.

17. What are the justifications of Random Forest and SVM for the classifiers? The hyperparameters should be defined and choices of the parameters should be justified.

Motivated by the SVM-based implementations of SV2 and SMRT-SV, we initially focused on using an SVM as the classifier. We also investigated other approaches, particularly for the per-variant model where much less training data would be available when building each classifier. Review of published comparisons[1] suggested that Random Forests (RF) could be very effective. We also tested "simpler" algorithms for the per-variant model, which we hypothesized might be more accurate when training data is limited, and the widely used, but more complex, XGBoost algorithm.

During development, we observed the combination of SVM for the single model and RF for the variant model to achieve consistently high accuracy across callsets and variant types compared to the other classifiers we tested. However, the differences in accuracy we observed between algorithms was generally minimal (typically within 1-1.5 percentage points). This suggested to us that the use of simulated training data and the feature implementations were more important drivers of genotyping accuracy than the choice of classifier.

For the single model, during development, we performed hyperparameter sweeps during training using scikit-learn's GirdSearchCV function with 5-fold cross validation. We did not observe a meaningful and consistent improvement in accuracy for GIAB SVs with the parameter sweep compared to the default parameters and so proceeded with the default parameters. That testing was originally performed outside of the NPSV code base. In response to this question, we integrated the parameter sweep into the NPSV code base. We repeated the earlier testing but observed similar results (no meaningful and consistent improvement in accuracy for GIAB SVs) and so disable the parameter sweep by default to reduce computation time. We added further description of the approach to parameter selection to the Methods section.

For the variant model where we potentially build thousands of classifiers, grid search during training is less practical. Here we tested different parameters for genotyping the GIAB tier 1 SVs. We similarly observed that the scikit-learn default parameters achieved generally high accuracy.

More generally, we sought to identify and implement a reasonable and effective machine learning approach. This evaluation represents the performance of a particular implementation. Our evaluation of potential classifier implementations is necessarily incomplete; there are too many possible algorithms to test and, as the reviewer notes, hyperparameter tuning is a subtle art. Thus, we fully expect that there are better algorithms or hyperparameters possible. Fortunately, the classifier is a modular component within NPSV that could readily be replaced to enable further experimentation and optimization.

[1] Rich Caruana and Alexandru Niculescu-Mizil. 2006. An empirical comparison of supervised learning algorithms. In Proceedings of the 23rd international conference on Machine learning (ICML '06). Association for Computing Machinery, New York, NY, USA, 161–168. DOI:https://doi.org/10.1145/1143844.1143865

18. What are the specific features that NPSV uses? It is not clear from the manuscript even what feature authors are referring to. The authors should clearly indicate and formulate how each feature is exactly computed. A lot of the methods must be re-written to be technically specific. I am afraid it is not beneficial unless these are defined exactly.

In the original submission the features were summarized in the main text and described in Supplemental Table S1. As we noted in the introduction to this response, we have reorganized and expanded the Supplemental Methods section with a more detailed description of the features used for classification.

Reviewer #3: In this manuscript, Linderman et al represented a novel method, NPSV, to genotype genomic structural variants (SVs) using short-read paired-end sequencing data. Benchmark of the method using SV calls from PacBio long reads revealed improved genotype concordance of NPSV against other algorithms. Overall, I think this method is of great value. I just have a few suggestions that I hope to help further improve the manuscript.

1. NPSV genotypes the putative SVs through simulating short-read data, aligning them against the reference genome, and comparing their alignment patterns with the actual data. This approach autonomously correct for multiple confounding issues in SV genotyping, including the size, type and genomic locations. However, this approach could be computationally expensive. It would be useful to systematically benchmark the computing cost of NPSV against other algorithms, including run time, cpu usage, and estimated computing cost on cloud (if applicable).

We have expanded upon that benchmarking in the results section and included runtime and memory usage for all tools in the supplemental.

2. NPSV showed lower genotyping accuracy for small SVs (<300bp) in tandem repeats (TRs), which is expected as short reads usually struggle in repeat sequences. Have the authors ever checked the genotype accuracy for large CNVs (typically >5Kb) that reside in segmental duplications? Segmental duplications are also of high repetitiveness, thus short-read data would have difficulty in these regions too, and it would be interesting to examine the performance of NPSV in these sequences.

In the GIAB call set, only 13 of the deletion SVs > 5 kbp considered in our tier 1 and tier 2 concordance analysis overlap (in any amount) with the UCSC Segmental Dups track. Of those, 76.9% (10/13) were genotyped correctly.

The Polaris datasets have more relevant deletions. NPSV default mode genotyping accuracy for large deletions overlapping a segdup is 77.8% (102/131) and 86.3% (176/204) for Polaris 2.0 and Polaris 2.1, respectively. As expected, this accuracy is lower than the genotype accuracy for larger SV deletions in general (> 90%).

The accuracy degradation we observe in the SV-plaudit callset is more striking. For deletions SVs > 5 kbp overlapping a segmental duplication, the accuracy is 37.0% (10/27). The difference between SV-plaudit and the Polaris datasets appears to be related, at least in part, to imprecise breakpoints: 14/17 discordantly genotyped SV-plaudit SVs reported imprecise breakpoints, with multiple variants having confidence intervals > 500 bp. As we note in the response to your next comment, accuracy for these variants is lower.

3. The evaluate for offset breakpoints is informative. However, for large CNVs, the breakpoints can be off by several hundred bases to kilobases. It would be interesting to extend the experiment for evaluation of large CNVs and inversions

As we noted in a response to reviewer 2, the SV-plaudit dataset, which is based on 1000 Genomes calls, enables us to investigate aspects of this question. The figure below shows counts of SVs (dis)concordantly genotyped by NPSV in the default mode (hybrid) by maximum confidence interval for different SV size bins. A larger fraction of the SVs with SVLEN > 1 kbp have imprecise breakpoints, and as you note, the confidence intervals (width of VCF CIPOS and CIEND fields) can be 100s or even 1000s of bases. Consistent with the discussion of offset variants in the manuscript, NPSV accuracy degrades for the larger imprecise variants.

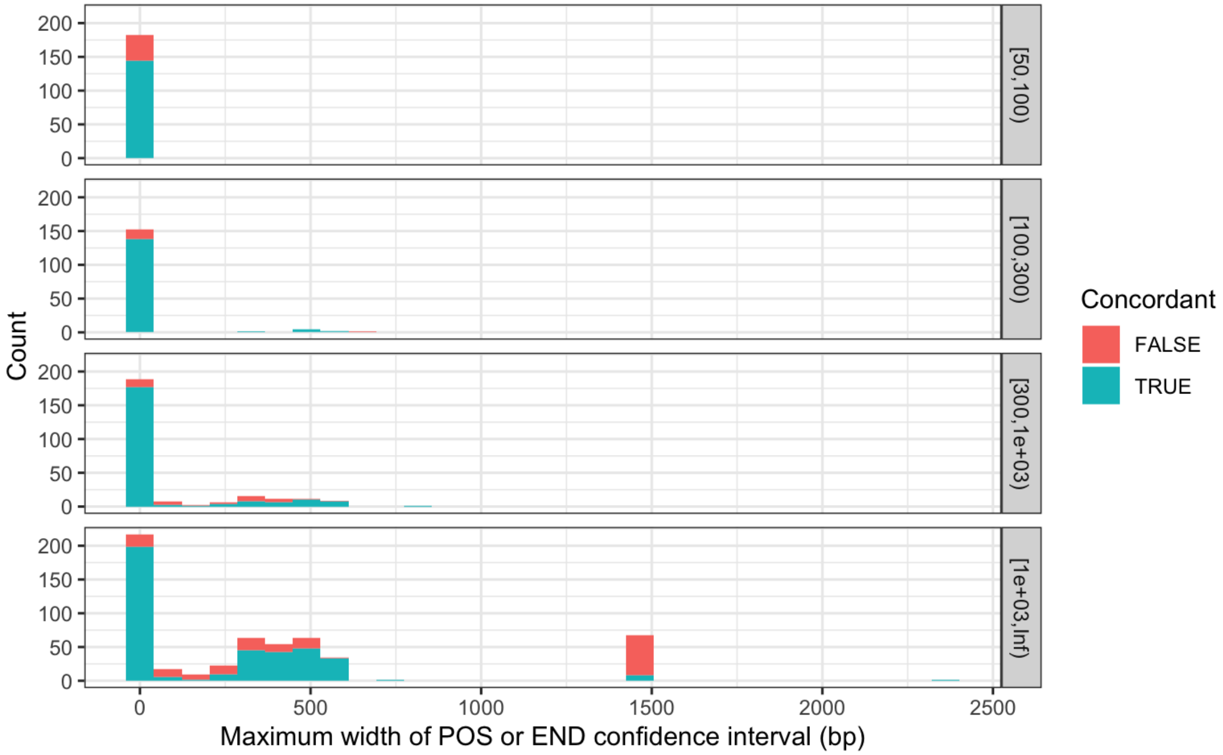

Figure 2: Histogram of genotype concordance for NPSV hybrid mode for SV-plaudit SVs by maximum width of breakpoint confidence intervals (CIPOS, CIEND)

We added a description of these observations for larger imprecise SVs to the "Offset SV representations" section of the results. At present, NSPV does not genotype inversions and so we cannot evaluate those variants.

Currently, NPSV features do not incorporate the confidence interval information. Doing so is an aspect of ongoing work and could potentially improve accuracy for these particular variants. However, as we note in the manuscript, in the ideal case we could correct/refine offset or imprecise SV descriptions as part of the genotyping process. The initial experiments we present suggest we can make some improvements in SV descriptions using the simulated data. Trying to refine the description of the SVs with breakpoint confidence intervals is an area of ongoing work.

More generally, we don't think that NPSV will be the most effective tool/approach for very large CNVs, especially those with imprecise breakpoints, both for the reasons described above and the increased computational cost for simulating very large variants.

Minor:

1. *I don't think "MEs" is defined in the manuscript?*

Thank you for catching that omission. We realize we defined MER but not ME. We have added that definition to the text.

2. It's surprising that there are 2 true-positive de novo deletions in HG002, as each genome is estimated to carry 0.2 de novo SVs (Werling et al 2018). Has any manual inspection or molecular validation done to confirm the quality of the de novo SVs, and rule out the possibility of mosaic CNVs arose during cell proliferation?

The events are those explicitly described in the GIAB paper. We had used the terms “true positive”, “false positive” and “correctly called” in the context of the Mendelian Error (ME) status from the GIAB paper. To avoid implications about validation we revised that paragraph and the table in the supplemental results to specifically quote or incorporate the description of those SVs in the GIAB paper (“likely de novo deletion” and a deletion in a “locus known to undergo somatic rearrangement”) and rewrote the description of the NPSV genotypes to more clearly focus on consistency with the GIAB ME status. For clarity and conciseness, we revised that section to focus on the two deletion MEs with clearly described genotypes as opposed to the insertions with ambiguous genotypes (from the GIAB paper: “two were insertions mis-genotyped as heterozygous in HG002 when in fact they were likely homozygous variant or complex”). The GIAB paper describes manually inspecting those variants but does not describe any other form of validation. Our manual review of the deletion pileups was consistent with the GIAB report.
