# Supplementary material for: NPSV: A simulation-driven approach to genotyping structural variants in whole-genome sequencing data
Source: Gigascience. 2021 Jul 1;10(7):giab046. doi: 10.1093/gigascience/giab046 (PMC8246072; doi:10.1093/gigascience/giab046)
Supplement: giab046_GIGA-D-20-00373_Revision_2 [file giab046_giga-d-20-00373_revision_2.pdf]

GigaScience

NPSV: A simulation-driven approach to genotyping structural variants in whole genome sequencing data

--Manuscript Draft--

**Manuscript Number:**	GIGA-D-20-00373R2
**Full Title:**	NPSV: A simulation-driven approach to genotyping structural variants in whole genome sequencing data
**Article Type:**	Technical Note
**Funding Information:**	National Institute of General Medical Sciences (P20GM103449) — Dr. Michael D Linderman<br>National Science Foundation (1827373) — Not applicable<br>National Heart, Lung, and Blood Institute (UM1HL098123) — Dr. Michael D Linderman<br>National Heart, Lung, and Blood Institute (U01HL153009) — Dr Bruce D. Gelb
**Abstract:**	Background: Structural variants (SV) play a causal role in numerous diseases but are difficult to detect and accurately genotype (determine zygosity) in whole genome next-generation sequencing (NGS) data. SV genotypers that assume the aligned sequencing data uniformly reflects the underlying SV or use existing SV call sets as training data can only partially account for variant and sample-specific biases.<br>Results: We introduce NPSV, a machine learning-based approach for genotyping previously discovered SVs that employs NGS simulation to model the combined effects of the genomic region, sequencer and alignment pipeline on the observed SV evidence. We evaluate NPSV alongside existing SV genotypers on multiple benchmark call sets. We show that NPSV consistently achieves or exceeds state-of-the-art genotyping accuracy across SV call sets, samples and variant types. NPSV can specifically identify putative de novo SVs in a trio context and is robust to offset SV breakpoints.<br>Conclusions: Growing SV databases and the increasing availability of SV calls from long-read sequencing make stand-alone genotyping of previously identified SVs an increasingly important component of genome analyses. By treating potential biases as a simulate-able "black box" NPSV provides a framework for accurately genotyping a broad range of SVs in both targeted and genome-scale applications.
**Corresponding Author:**	Michael Linderman<br><br>UNITED STATES
**Corresponding Author Secondary Information:**	
**Corresponding Author's Institution:**	
**Corresponding Author's Secondary Institution:**	
**First Author:**	Michael D Linderman
**First Author Secondary Information:**	
**Order of Authors:**	Michael D Linderman<br>Crystal Paudyal<br>Musab Shakeel<br>William Kelley<br>Ali Bashir<br>Bruce D. Gelb

Powered by Editorial Manager® and ProduXion Manager® from Aries Systems Corporation

Order of Authors Secondary Information:	
Response to Reviewers:	The change tracking has been removed, ORCIDs added for all authors where available and the data availability section has been updated with the gigaDB citations.
Additional Information:	
Question	Response
Are you submitting this manuscript to a special series or article collection?	No
**Experimental design and statistics**<br><br>Full details of the experimental design and statistical methods used should be given in the Methods section, as detailed in our Minimum Standards Reporting Checklist. Information essential to interpreting the data presented should be made available in the figure legends.<br><br>Have you included all the information requested in your manuscript?	Yes
**Resources**<br><br>A description of all resources used, including antibodies, cell lines, animals and software tools, with enough information to allow them to be uniquely identified, should be included in the Methods section. Authors are strongly encouraged to cite Research Resource Identifiers (RRIDs) for antibodies, model organisms and tools, where possible.<br><br>Have you included the information requested as detailed in our Minimum Standards Reporting Checklist?	Yes
**Availability of data and materials**<br><br>All datasets and code on which the conclusions of the paper rely must be either included in your submission or deposited in publicly available repositories (where available and ethically appropriate), referencing such data using a unique identifier in the references and in	Yes

the “Availability of Data and Materials” section of your manuscript.<br><br>Have you have met the above requirement as detailed in our Minimum Standards Reporting Checklist?	

Powered by Editorial Manager® and ProduXion Manager® from Aries Systems Corporation

Manuscript

Click here to access/download;Manuscript;NPSVManuscript_forproduction.do

NPSV: A simulation-driven approach to genotyping structural variants in whole genome sequencing data

Running Head: Genotyping SVs with simulation

Michael D. Linderman [0000-0002-9643-7148] [1*], Crystal Paudyal [0000-0001-5718-4515] [1], Musab Shakeel [0000-0003-1706-4652] [1], William Kelley,[1] Ali Bashir [0000-0002-5240-9604][2†], Bruce D. Gelb [0000-0001-8527-5027][3†]

[1]Department of Computer Science, Middlebury College, Middlebury, VT, USA
[2]Google, Mountain View, CA, USA
[3]Mindich Child Health and Development Institute and the Departments of Pediatrics and Genetics and Genomic Sciences, Icahn School of Medicine at Mount Sinai, New York, NY, USA

[†]These authors contributed equally

*Corresponding Author:

Michael D. Linderman
mlinderman@middlebury.edu
Department of Computer Science
Middlebury College
14 Old Chapel Road
Middlebury, VT 05753
(802) 443-5737

Abstract

Background: Structural variants (SV) play a causal role in numerous diseases but are difficult to detect and accurately genotype (determine zygosity) in whole genome next-generation sequencing (NGS) data. SV genotypers that assume the aligned sequencing data uniformly reflects the underlying SV or use existing SV call sets as training data can only partially account for variant and sample-specific biases.

Results: We introduce NPSV, a machine learning-based approach for genotyping previously discovered SVs that employs NGS simulation to model the combined effects of the genomic region, sequencer and alignment pipeline on the observed SV evidence. We evaluate NPSV alongside existing SV genotypers on multiple benchmark call sets. We show that NPSV consistently achieves or exceeds state-of-the-art genotyping accuracy across SV call sets, samples and variant types. NPSV can specifically identify putative *de novo* SVs in a trio context and is robust to offset SV breakpoints.

Conclusions: Growing SV databases and the increasing availability of SV calls from long-read sequencing make stand-alone genotyping of previously identified SVs an increasingly important component of genome analyses. By treating potential biases as a simulate-able “black box” NPSV provides a framework for accurately genotyping a broad range of SVs in both targeted and genome-scale applications.

Keywords

Structural variants, Next generation sequencing, Whole genome sequencing

Findings

Background

Structural variants (SVs) play a causal role in numerous diseases[1]. However, our ability to detect and analyze disease-causing SVs in short-read whole genome sequencing (WGS) data can be limited by inaccurate genotyping (determining zygosity)[2,3]. While numerous tools integrate SV discovery and genotyping[4–6], our focus here is “stand-alone” genotyping of putative SVs identified by discovery tools and/or obtained from the literature/SV catalogs [7]. Stand-alone genotyping is a critical step in ensemble pipelines that integrate multiple SV discovery tools, in clinical workflows, where we seek to accurately genotype known pathogenic SVs (e.g., from dbVar[8]) alongside detecting novel SVs, and in population studies, which generate “squared-off” genotypes for all variants in all samples[7].

SVs, defined here as variants greater than 50 bp[9], are similar in size to or larger than the read length of short-read next generation sequencers (NGS) and, thus, typically cannot be detected directly. Instead SVs must be inferred from secondary features in the sequencing data such as split reads, discordant read-pairs and read depth[9]. As a result, the precision and recall for detecting and genotyping SVs in NGS data can be much lower than for single nucleotide variants and short indels[4,7,10–13]. Long-read sequencing (read lengths of 10+ kbp) improves the recall and precision of SV detection (the long reads span more events and can be more reliably mapped)[14–16]. However, long-read sequencing is more expensive than NGS[17], so many more samples have been and will continue to be sequenced with NGS technologies. Thus, despite the growth in long-read sequencing, there is a need to develop improved NGS SV genotyping tools, including to genotype those SVs first (and exclusively) detected with long-read sequencing.

Existing SV genotyping tools[18–26] (see Chander et al.[7] for a recent comparison) exclusively target specific variant types/sizes, employ parametric (i.e., fixed-size[27]) models of SV evidence, and/or are trained on existing genome-wide call sets. These approaches assume that different SV call sets are similar and/or that the aligned sequencing data consistently and uniformly reflects the underlying variant (e.g., the read depth is proportional to copy-number, alternate alleles are identified at a consistent rate and/or consistent breakpoint features will be observed across all variants). However, these assumptions do not hold for all variants. The different types of SVs, range of SV sizes, different genomic contexts and different sequencers/pipelines, all of which influence the available evidence for predicting the SV genotype, motivate an ensemble of approaches, each optimized for a specific subset of SVs[10,28]. NGS simulation is a possible strategy to enable automatic ensemble creation. For example, for select SVs from the 1000 Genomes Project, Chu *et al.* showed that training the GINDEL SV genotyper on simulated data could achieve genotyping accuracy within a few percentage points of models trained on held-out data[29].

Here, we propose the Non-Parametric SV (NPSV) genotyper. NPSV extends current ensemble methods by automatically creating classifiers for predicting SV genotypes optimized for the specific SVs and sample under analysis and even a single, specific, SV. In this non-parametric approach, the number of models can grow to capture genomic-region, sequencer and pipeline-specific SV evidence. NPSV performs detailed simulation of the putative SVs to be genotyped. The simulated data, which are representative of the actual observed sequencing data, are used to train sample- and variant-specific classifiers for predicting SV genotypes. In contrast to training data sourced from existing SV call sets, by using simulation we can generate representative

training data for any putative SV, not just those previously observed, with accurate sequence-resolved breakpoints and “ground truth” genotype labels.

We present a rigorous evaluation of NPSV genotyping accuracy across multiple truth sets in the HG002 and NA12878 reference samples. We compare NPSV to similar standalone SV genotyping tools (that accept a VCF of putative SVs and aligned reads as input and predict the SV genotype), chosen to be representative of different alignment, graph and machine learning-based SV genotyping methods: Delly2[18], SVTyper[19], svviz2[20], Paragraph[25], GraphTyper2[21], SV2[22] and GenomeSTRiP[26]. We show that NPSV consistently achieves similar or better genotyping accuracy across the different datasets, samples and variant types, can sensitively and specifically identify putative *de novo* SVs in a trio context and is robust to offsets in SV breakpoints.

Simulation-driven SV genotyping

The NPSV dataflow is shown in Figure 1a. The inputs are the aligned reads (BAM/CRAM file), termed the “actual” data, and a VCF file of putative sequence-resolved deletion and insertion SVs. For each putative SV and possible genotype, NPSV generates synthetic short-read datasets using an NGS simulator configured to match the actual data (bottom path in Figure 1a). We process the simulated datasets with the same alignment pipeline as the actual data and then extract re-alignment, read-pair and coverage SV features from each simulated replicate. The features extracted from the simulated data are used to train sample- and variant-specific classifier(s) to predict the genotype from the SV evidence similarly extracted from the actual sequencing reads. The simulation, feature extraction and classification approaches are described in more detail in the Methods (NPSV Genotyping Algorithm) with the features specifically described in Table S1.

Figure 1b shows the simulated and actual SV evidence for an example homozygous alternate 822 bp deletion in the Genome in a Bottle (GIAB) HG002 call set[27], as would be generated to train a variant-specific classifier. The actual data is most consistent with the simulated homozygous alternate genotype. This SV is the deletion of one repeat of a tandem repeat. Due to the underlying repetitive sequence, no reads were uniquely re-aligned to the SV's alternate allele and no alternate spanning fragments were identified. The simulated data shows that the absence of both of those features is consistent with the alternate allele for this SV (and pipeline) and is not an indication of a homozygous reference genotype as might otherwise be expected (indicated by the actual and simulated features in the first two panels "massing" on the y-axis). The NPSV variant-specific classifier correctly genotyped this variant as homozygous alternate, while genotypers that exclusively use realignment, split-read and/or spanning read evidence did not.

NPSV implements two genotyping approaches: a 1) "variant" model, like described above, which creates variant-specific classifiers trained on 100 replicates per variant per zygosity (i.e., 300*n* synthetic samples for *n* variants), and a 2) "single" model that creates a single sample-specific genome-wide classifier for each variant type (e.g., deletions, insertions) trained on one replicate per variant per zygosity (i.e., *3n* synthetic samples for *n* variants). The former approach is more computationally demanding but can be applied at any scale, including for just a single SV in a single sample. To reduce the computational burden, a "hybrid" model only builds variant- specific classifiers for smaller SVs (< 1 kbp by default) and uses the single model for larger SVs. We generally observed the hybrid model to be most accurate for deletions and the single model to be most accurate for insertions and so set that as the default configuration.

Results

Genotyping accuracy

We evaluated NPSV and the comparison SV genotypers with multiple SV call sets across two samples: the GIAB version 0.6 call set for HG002[30], and the Polaris 2.0, Polaris 2.1 and SV-plaudit call sets for NA12878[31,32]. Genotype counts for each call set are shown in Supplemental Table S2. Using the call set SVs as the input, we report the genotype concordance, i.e., the fraction of predicted genotypes that exactly match the call set genotypes, and the non-reference concordance, which treats heterozygous and homozygous alternate genotypes as equivalent. The call sets and evaluation are described in more detail in the Methods.

Figure 2 shows the genotyping accuracy for NPSV and comparison tools for all truth sets. As a result of randomization in the simulations, SV sampling and classifier training, NPSV genotyping is not deterministic. In Figure 2 we show the mean accuracy for 10 complete NPSV genotyping runs and report the mean and standard deviation in Supplemental Tables S3-S6.

Figure 2a (Supplemental Table S3) shows the genotyping accuracy for GIAB SVs in the high-confidence tier 1 regions (6449 DEL and 6462 INS SVs) and in the tier 1 regions combined with lower-confidence tier 2 SVs (8370 DEL and 8413 INS SVs). NPSV achieves similar or better exact genotype concordance and non-reference concordance than the comparison tools for both deletions and insertions. For SVs in tier 1 regions, NPSV improves genotype and non-reference concordance for deletions and insertions by 0.8-2.1 percentage points. Figure 2b (Supplemental Table S5) shows the genotyping accuracy for the NA12878 truth sets (1143 DEL SVs in SV-plaudit, 8073 DEL and 6246 INS SVs in Polaris 2.0, and 20610 DEL and 12028 INS SVs in Polaris 2.1). NPSV generalizes across these datasets, achieving similar or better accuracy than the best comparison SV genotypers across all three datasets and both insertions and deletions.

Precision, recall and F1 scores for genotyping homozygous reference vs. non-reference SVs are shown in Table S4 and Table S6. We observed NPSV to achieve similar or better F1 scores than the comparison genotypers, albeit often with increased recall and reduced precision. For the Polaris 2.1 call set, which is enriched for homozygous reference calls and thus reflective of the common population genotyping use case, the mean DEL and INS recall are 0.939 and 0.985, respectively: the DEL and INS precision (false discovery rate) are 0.820 (0.180) and 0.945 (0.055). Given the small the variance across all metrics for NPSV, for concision, the remaining analyses utilize a single genotyping run.

Table S7 shows the genotype concordance for NPSV single and variant models for GIAB SVs in tier 1 regions grouped by SV length (SVLEN), the difference in length of reference and alternate alleles. Concordance generally increases with increasing SV length as read-pair and other features become more informative and a smaller fraction of variants overlap repetitive regions (see below). For deletions larger than 1 kbp, the single model showed increased accuracy. Those results motivated the default 1 kbp threshold for the hybrid approach, which uses the single model for larger SVs where that approach is more accurate and for which simulating an SV is more computationally demanding, and reserves the more computationally expensive but also potentially more accurate variant model for smaller variants. For GIAB insertions, the single model is more accurate than the variant model for all variant sizes. As noted above, based on these results, we set the NPSV default configuration to use the hybrid approach for deletions and single model for insertions. Figure S3 shows the genotype concordance for all call sets and tools grouped by SV length along with the underlying SV length distributions.

Due to the repetitive sequence, SVs in tandem repeats (TRs) are more difficult to accurately genotype. NPSV genotype concordance for GIAB DEL and INS SVs in tier 1 regions

overlapping a TR > 100 bp (as annotated by GIAB) was 77.5% and 69.0%, respectively, compared to 96.5% and 92.1% for DEL and INS SVs not overlapping a TR > 100 bp. Figure S4 shows the genotype concordance for all NPSV modes for GIAB SVs in tier 1 regions grouped by SV length and whether the SV overlaps a TR > 100 bp. The [50,100) and [100, 300) size bins are enriched for SVs overlapping a TR > 100 bp, contributing to the reduced genotyping accuracy for these smaller SVs reported above.

To evaluate the use of the NPSV stand-alone genotyper with SVs identified with SV discovery tools (as opposed to benchmark call sets), we re-genotyped SVs called with Lumpy[33]/SVTyper[19] and Manta[34] in HG002. Using the discovery SVs as the input, Table 1 shows the genotyping accuracy and Table S8 shows precision, recall, and F1 scores compared to the GIAB SVs in tier 1 regions. To focus on genotyping accuracy, SVs that were not detected (“no-calls”) were excluded from the concordance calculation. For the Lumpy call set the difference in genotyping accuracy is primarily driven by the count of putative false positives, which in turn is sensitive to the criteria for matching the call set to truth set SVs. Some of the putative false positive SVs may be true positive SVs (i.e., non-reference) that have sufficiently different breakpoints compared to the truth set SV so as not to match during concordance analysis. When the required size similarity is relaxed from 70% to 30%, 150 more Lumpy SVs matched GIAB truth set SVs and the exact genotype concordance (non-reference concordance) for NPSV, 84.9% (91.3%), and Lumpy/SVTyper, 82.8% (91.9%), became more similar. The impact of offset or imprecise SV descriptions is described further below.

Trio analysis

We evaluated SV genotyping in a trio context using the HG002 trio. Table 2 shows the mendelian error rate (MER) and counts of different types of mendelian errors (MEs) for GIAB

SVs in tier 1 regions. The NPSV MER for both deletions and insertions are greater than the MER for some of the existing genotypers, e.g., svviz2. However, most of the NPSV MEs are variants with low confidence genotypes and thus can be specifically filtered out based on the NPSV-reported genotype quality (GQ); for example 94% of ME deletions in tier 1 regions have a minimum GQ < 10 (among all trio members). The highest-quality NPSV deletion ME in the tier 1 regions was explicitly reported by Zook *et al.*[30] as a “likely *de novo* deletion”. Supplemental Table S9 lists the trio genotypes, minimum GQ and GQ ranking for that deletion and a second deletion ME reported by *Zook et al*. in a locus known to undergo somatic rearrangement. Consistent with Zook *et al.* NPSV genotyped the two deletions as *de novo*. NPSV in variant mode reported the two deletions as the most confident ME deletions in the tier 1 regions, and the hybrid mode reported the variants among the top 4 most confident.

Offset SV representations

As shown in Figure S4, the set of GIAB SVs with discordant NPSV genotypes (i.e., the NPSV genotype does not match the GIAB genotype) is enriched for variants that overlap TRs. Across all NPSV modes, 86+% of GIAB discordant deletions and 68+% of discordant insertions in tier 1 regions are annotated in the GIAB call set as overlapping a TR > 100 bp, while less than 44% of concordant deletions and 30% of concordant insertions SVs are similarly annotated. Differences between the description of the putative SV (breakpoints and sequence change) and the true SV is one of the factors that contribute to genotyping errors for SVs in these repetitive regions (and more generally)[25]. We manually reviewed the pileup for 10 randomly selected deletions discordantly genotyped by NPSV in variant mode; 8/10 SVs were offset from the location indicated by long-read PacBio sequencing data.

To evaluate the impact of offset breakpoints more generally, we matched the GIAB SVs (PASS variants only) in tier 1 regions to corresponding SVs called by PBSV[35] in PacBio long-read sequencing data (4114/4203 deletions and 5157/5443 insertions successfully matched). Making the assumption that the PacBio SV calls have correct breakpoints, we infer the offset from the distance between the GIAB breakpoints and the breakpoints identified in the long-read data (modeled on the approach in Chen et al.[25]). Figure 3 shows genotype concordance for SVs grouped by the breakpoint offset (the same analysis for select comparison tools is included in supplemental Figure S3**)**. For deletions, we observe an expected negative association between breakpoint offsets and genotyping accuracy; genotype concordance is 85+% for offsets up to 10 bp (and 95+% for no or single base offsets), decreasing to 49.6% for SVs with breakpoint offsets greater than 50 bp. At larger offsets, the variant model increasingly outperforms the single model suggesting that the variant-specific classifiers may be better able to model the specific genomic context around offset deletions. For insertions we observe a similar negative association between breakpoint offsets and genotyping accuracy, although with a plateau for offsets of 1-20 bp. Much of the genotype concordance is recovered when using the long-read-derived SV calls as the input call set instead of the GIAB SVs (solid line in Figure 3).

The challenge of offset or imprecise breakpoints is further observed in the SV-plaudit call set. The majority of SV-plaudit variants greater than 1 kb (410/551) have imprecise breakpoints (including SVs with breakpoint confidence intervals of 100s or 1000s of bases). NPSV genotype concordance was 93.6% (132/141) for SVs with exact breakpoints, dropping to 63.7% (261/410) otherwise.

To investigate the potential for correcting SV descriptions using only the NGS data, we experimentally extended NPSV to propose and select among possible alternate alignments for an

SV. We hypothesize that the actual data is most similar to the simulated data for the correct SV description and genotype, and thus we could identify a better SV representation based on the distance between the actual and simulated SV evidence. For deletions of one or more copies of a TR, we proposed up to 10 different alignments of the deletion within the repetitive region, choosing the SV description where the real data is closest to the non-reference synthetic data. The SV proposal algorithm is described in more detail in the Supplemental Methods. When applied to the GIAB callset SV proposal increases the sensitivity for calling heterozygous and homozygous alternate genotypes at the cost of smaller decreases in precision; the net effect is an increase in genotyping accuracy (genotype concordance of 87.8% vs. 87.2% for SVs in tier 1 regions, 83.7% vs. 82.9% for SVs in tier 1 and 2 regions) and F1 scores (0.940 vs. 0.933 for SVs in tier 1 regions, 0.926 vs. 0.914 for SVs in tier 1 and 2 regions) (Supplemental Table S10).

Computational requirements

The simulation process is computationally intensive, but also readily parallelizable. NPSV simulation and feature extraction are multi-threaded across variants. On a 36-core compute node (dual 18-core Intel Xeon 6140 2.3 GHz CPUs) genotyping 16,871 GIAB SVs required 21.1 hours (wall clock time as determined by the time utility); employing the single, variant and hybrid approaches exclusively required 53.3 minutes, 39.6 hours and 31.2 hours, respectively. Maximum memory resident set size was 22.1 GB as determined by the SLURM cluster manager (17.7 GB, 39.0 GB and 24.3 GB for the single, variant and hybrid modes). The NPSV preprocessing step, which generates sequencing statistics, e.g., insert size distribution, used in simulation and features extraction, is designed to use a combination of goleft[36], a fast alignment analysis tool, and metrics already calculated as part of a typical genome analysis pipeline, e.g. with the Picard tools[37]. In that approach, less than one minute is required for pre-

processing. The currently un-optimized fallback preprocessor required 2.4 hours for the 25.5× HG002 BAM file. Table S10 lists execution time and resident set size for all of the comparison tools running on the same system.

Discussion

NPSV is a novel stand-alone SV genotyper that simulates putative SVs to train sample and variant-specific machine learning classifiers. NPSV consistently achieved similar or better genotyping accuracy than the comparison SV genotypers across both variant types and all truth sets (Figure 2), including compared to the tools used to construct those truth sets (e.g., svviz2 for GIAB and Paragraph for Polaris). NPSV successfully and specifically identified the putative *de novo* SV deletions reported by GIAB. Improvements of 1-3 percentage points in genotyping accuracy translates to 10s-100s fewer incorrect genotypes per genome. Those incorrect genotypes leave cases unresolved, consume limited budgets for manual review and validation testing, and dilute downstream analyses.

SV call sets and reference databases can contain many SVs with incorrect or imprecise descriptions. For example, the clustering of SVs with similar but unique sequence changes during the construction of the GIAB call set reduced the number of SVs 2.3-fold[30], indicating many of the putative SVs did not have a single consensus description. Incorrect or imprecise SV descriptions can negatively impact genotyping accuracy[25]. NPSV maintains genotype concordances of 85+% (DEL) and 81+% (INS) for offsets up to 10 bp; similar to or more robust than comparison tools (Figure 3, Figure S4).

Making the SV features even more robust to incorrect/imprecise SV descriptions could improve genotyping accuracy. However, in a strict interpretation of the precise sequence-resolved SVs in the GIAB call set, genotyping a putative SV with an incorrect description as non-reference

would be inaccurate as that specific alternate allele is absent. Ideally, we would want to identify the correct SV descriptions as part of the genotyping process. We observed substantial increases in genotyping accuracy when using SVs called in long-read sequencing data as the input call set, suggesting there is an opportunity to further improve genotyping accuracy by refining the SV descriptions. We extended NPSV to select among alternative alignments for deletion SVs based on the similarity between the actual and simulated NGS data. The alternate representations increased the sensitivity for detecting non-reference genotypes with a net increase in genotyping accuracy and F1 score for detecting non-reference genotypes compared to the original SV descriptions (Table S9). A substantial gap remains, though, between NPSV's NGS-only approach for refining SV descriptions and the accuracies observed when genotyping the long read-derived calls (Figure 3). We are actively working on improving all aspects (SV proposal, NGS simulation fidelity, features, and the similarity metric) of the SV refinement algorithm. Since NPSV simulates the expected alleles, it is limited to sequence-resolved SVs with discrete genotypes, and does not genotype "position independent" SVs, e.g., high-copy number duplications. At present, NPSV only supports biallelic deletions and insertions and treats each SV independently. It does not currently genotype inversions or other SV types. However, the underlying method can be extended to support other SV types, e.g., inversions, and more complex variants/genotypes, e.g., compound heterozygous genotypes or multiple SVs on the same haplotypes. We hypothesize that the simulation-based approach, which is not dependent on the previous generation of representative training data, may be particularly useful for complex SVs. Minimal high-quality "ground truth" data is available for these sites; GIAB, for example, largely excluded complex SVs from the benchmark call set and most of the genotype errors identified in manual review were identified as complex[30].

The simulation process can be computationally intensive, particularly when simulating many replicates to build per-variant classifiers. However, since the variant-specific classifiers can be built at the granularity of a single variant, they can be employed in a targeted fashion, e.g., on SVs with low confidence genotypes or in repetitive regions, to model the biases introduced by the genomic region, sequencer and/or the analysis pipeline. Alternately when genotyping the same call set across multiple samples, the simulated training data could be reused. Preliminary experiments using the simulated training data generated for the parental samples to genotype the GIAB SVs in HG002 showed similar genotyping accuracy. When using shared training data, the simulation costs scale with the call set size, not the product of the call set and cohort sizes. Optimizing NPSV for large cohorts is an area of ongoing work. Large, highly consistent cohorts, such as gnomAD, can use other samples as the reference panel[38] but may have few and/or potentially ambiguous examples of extremely rare variants/genotypes. NPSV can effectively create a synthetic "reference panel" for all zygosities, for any variant, in any number of samples (including a single genome).

Conclusions

Here we present NPSV, a stand-alone SV genotyper for WGS data. Instead of attempting to develop a model for the complex and interconnected effects of the genomic region, sequencer and alignment pipeline on the observed SV evidence, NPSV employs detailed simulation of the sequencing process to train sample- and variant-specific classifiers for predicting SV genotypes. Since NPSV can generate relevant training data for any variant(s), at any granularity, it supports a range of targeted (a single variant) and large-scale (whole genome) SV genotyping applications. We showed that NPSV consistently achieves similar or improved genotyping

accuracy for benchmark call sets. Looking forward, NPSV's simulation-based approach provides a framework for genotyping the important "long tail" of SVs that are rare, complex and/or exclusively discovered with long-read technologies, and thus lack high-quality representative training examples.

Methods

NPSV Genotyping Algorithm

NPSV is a Python-based tool for stand-alone genotyping of sequence-resolved SV insertions and deletions. The inputs are the aligned reads (BAM/CRAM file), termed the "actual" data, and a VCF file of putative SVs. NPSV produces a copy of the input VCF with predicted SV genotypes. Prior to genotyping, a preprocessing step estimates the mean, per-chromosome and per-GC fraction coverage, and the insert size distribution. Those statistics inform the simulation and feature extraction. Many of those metrics are often already generated as part of the genome analysis pipeline and so do not need to be re-computed. In this evaluation, NPSV internally runs indexcov[36] and uses metrics previously computed with the Picard tools[37]. NPSV can also generate those statistics directly if needed using bedtools[39], SAMtools[40] and indexcov[36]. For each putative SV and possible genotype, NPSV generates one or more synthetic short-read datasets (termed replicates) using the ART NGS simulator[41] configured to model the actual sequencing data i.e., sequencer error model, read length, insert size distribution and coverage. In this evaluation we align the simulated WGS data with BWA-MEM[42] and mark duplicates with samblaster[43] to mimic the BCBio pipeline[44] used to align the actual data (along with SAMtools[40] and sambamba[45] for format conversion and sorting). The SV features extracted from the simulated replicates (and randomly simulated regions in the genome, see below) are

used to train sample- and variant-specific classifier(s). The SV features extracted from the actual data for putative SVs are only used to predict the genotypes (and not for training).

Features extracted from the simulation of the homozygous references genotype, i.e., the absence of the putative SV, can exhibit low variance, negatively impacting genotyping accuracy. To generate a more realistic “null” model, by default, NPSV generates the training data for homozygous reference genotypes by extracting features from the actual alignments for size-matched variants randomly sampled from the genome[46]. For haploid sex chromosomes, size matched variants are sampled from the sex chromosomes, otherwise variants are sampled from the autosome (and the X chromosome for SVs called on a diploid X chromosome).

NPSV extracts or derives the allele, spanning read and coverage SV features listed in Table S1 and described in more detail in the Supplemental Methods. NPSV counts the reference and alternate reads by locally realigning read pairs to the reference and alternate sequences (derived from the putative SV description) using BWA[42] (via SeqLib[47]) and a read pair-aware alignment scoring metric adapted from svviz2[20]. Only reads originally aligned within some flanking distance (default of 99th percentile of the insert size) of the putative SV breakpoints are realigned. We extract insert size probability-weighted counts of spanning reads (adapted from SVTyper[19]), counts of clipped reads (adapted from SMRT-SV2[23]), and the mean event depth relative to flank regions, chromosome and regions of similar GC coverage features (DHFFC, DHFC, DHBFC adapted from duphold[48]) from the actual (original) alignments.

NPSV currently implements a Support Vector Machine (SVM) classifier for the single model and a random-forest (RF) classifier for the variant model using the scikit-learn framework[49]. The specific features used with each classifier are listed in Table S1. We observed this combination to achieve consistently high accuracy across variant types and call sets, although the

differences in accuracy between classifier algorithms was typically small (1-1.5 percentage points). Data is centered and normalized to unit variance (using StandardScaler) prior to training the SVM (using a radial basis function kernel) with the same scaling used for genotyping. When training the single SVM model, NPSV can perform a grid search of the C (1, 10, 100, 500, 1000, 5000, 10000) and gamma ("scale", 0.001, 0.0055, 0.01, 0.055, 0.1, 0.55) hyperparameters with 5-fold cross validation [23], however we did not observe consistently improved accuracy over default parameters for the GIAB call set and so disable the parameter sweep by default to reduce the training time. When training the single-model classifier, the training data is optionally filtered by genomic region. For the GIAB call set we excluded data outside the GIAB tier 1 regions. When training the per-variant classifiers, observations with features more than 5 standard deviations from the mean are excluded. To reduce execution time for the per-variant model, by default, we do not implement parameter sweeps during training. Evaluation of the RF-based variant model on the GIAB call set with different numbers of trees (10, 50, 100, 200) and maximum tree depth (variable, 3) indicated the default parameters (100 trees with variable depth) achieves high accuracy with reasonable execution time. The final genotypes and genotype quality (GQ) are determined from the label and class probabilities predicted by scikit-learn.

Truth Sets

We evaluated NPSV and the comparison SV genotypers with deletion and insertion SVs in the GIAB version 0.6 call set (GRCh37) for HG002, and the Polaris 2.0 (GRCh37), Polaris 2.1 (GRCh38) and SV-plaudit (GRCh37) call sets for NA12878. The truth sets were obtained from the GIAB FTP site[50], Polaris repository[31] and SV-Plaudit supplemental materials[51]. GIAB SVs smaller than 50 bp or larger than 15 Mbp, SVs outside the GIAB tier 1 and 2 regions, SVs without genotypes and filtered (i.e., not PASS) SVs, except for those variants filtered as

"LongReadHomRef" (i.e., "long reads supported homozygous reference for all individuals"), were excluded. SV-plaudit and Polaris SVs smaller than 50 bp or larger than 15 Mbp, SVs without genotypes and filtered SVs were similarly excluded. In the SV-plaudit report[32] nine researchers manually inspected SVs called in NA12878 by the 1000 Genomes Project[10]. The researchers were shown visualizations of data for the NA12878 trio and asked: "Does the sample support the variant type shown? […]", with the possible answers "True", "False", or "denovo". Only SVs for which more than 50% of the curators reported the sample supports the variant were retained. Almost all the curated SVs were deletions, so we limited the SV-plaudit analysis to deletions. Supplemental Table S2 lists the counts of each genotype in the different truth sets.

Short-read Sequencing Data and SV Discovery

We genotyped the GIAB SVs in a subset of the NIST Illumina HiSeq 2500 2×148 PCR-free WGS data[52–55] with coverage representative of typical WGS (mean coverage of 25.5×, 20.4× and 24.7× for HG002, HG003 and HG004 respectively). We aligned the WGS reads to GRCh37 and performed point variant calling and SV discovery (using Lumpy[33]/SVTyper[19] via smoove[56] and Manta[34]) with version 1.0.9 of the BCBio pipeline using the default BWA and GATK-based configuration[44]. We genotyped the NA12878 SVs in the Illumina Platinum Genomes 2×100 WGS data[57,58] (mean coverage of 50.5×). We aligned the NA12878 WGS reads to GRCh37 and GRCh38 with version 1.2.3 of the BCBio pipeline.

Comparison Tools

We compared NPSV to a representative set of stand-alone SV genotyping tools. The Delly2 (v0.8.3) genotyping module[18], SVTyper (v0.7.1) [19] and GenomeSTRiP (v2.00.1958) [26] predict the genotype using a parameterized model incorporating multiple forms of evidence, e.g.,

depth, split-reads and read-pairs, extracted from original alignments. The svviz2 (commit b2c5126)[20] reporting module predicts the genotype assuming a binomial model for counts of reads realigned to the SV alleles with BWA. Paragraph (v2.4a)[25] and GraphTyper2 (v2.5.1)[21] employ a parametric model of reads realigned to a graph representation of the SV. SV2 (v1.5)[22] uses an SVM classifier trained on features extracted from 1000 Genomes data. Unless otherwise noted, all tools were run with the truth set VCFs and BAMs produced by the BCBio pipeline as the inputs. SV2, SVTyper and GenomeSTRiP do not support the insertion SVs in the GIAB and Polaris call sets and so were evaluated on the deletion SVs only. Prior to genotyping with Paragraph, we normalized the VCF to add a padding base for complex variants. The svviz2 genotypes were extracted from the "GT_mapq" field in the report to generate a genotyped VCF (we observed the "mapq" genotypes to generally be the most accurate for the GIAB call set). For SV2, variants called by GATK haplotype caller (as implemented in the BCBio pipeline) were used as the "SNV" input. For GraphTyper2, the GIAB tier 1 and 2 BED file was used to generate the regions for genotyping the GIAB HG002 call set, while the entire chromosomes were used as the regions for the NA12878 call sets; the "AGGREGATE" model was used as the output genotypes. GraphTyper2 converts insertions to duplications, those SVs were converted back to the call set representation to facilitate concordance analysis. Delly modifies the representation of some indel SVs such that the modified SV is no longer matched to the corresponding SV in the truth set during evaluation, reducing the reported concordance by up to 0.3 percentage points. Each tool was run with its default parameters and thus the results presented here may not represent the best possible performance that could be achieved with expert tuning of the available configuration parameters. For example, GenomeSTRiP's high rate of "no-calls" (./.) for some smaller Polaris SVs can be impacted by the "minimum length to

include depth-based genotype likelihoods" depth.effectiveLengthThreshold parameter (default of 200)[29]. The VCF FILTER annotations introduced by Delly, GraphTyper, Paragraph and SV2 reduced genotyping accuracy (filtered genotypes are treated as "no calls" during concordance analysis) and so were ignored in all evaluations.

This evaluation does not exercise all of the capabilities of the different comparison tools, which may support other variant types, e.g., inversions, not yet implemented in NPSV, provide other features, such as visualization, or are explicitly designed for efficient population-scale genotyping as opposed to the single sample and trio analyses performed here.

Evaluation

We measured genotyping accuracy using Truvari[59], modified to report the genotype confusion matrix[60]. Figure S1a-b shows the definitions of concordance metrics calculated from the confusion matrix when using the "truth" SVs as the input to SV genotyping. Figure S1c-d shows the definition of the concordance metrics when using the output of an SV discovery tool as the input to the SV genotyper.

MEs were identified in autosomal regions using BCFTools[61]. We categorized MEs as a heterozygous or homozygous *de novo*, or other (e.g., homozygous alternate proband with a homozygous reference parent).

To evaluate the impact of imprecise breakpoints, we computed the genotype concordance for GIAB deletion SVs in tier 1 regions grouped by the maximum offset between the GIAB SV breakpoints and the corresponding SV breakpoints called in long-read sequencing data[25]. We used SV calls generated by PBSV 2.2.1 in PacBio CCS reads (obtained from the GIAB FTP repository). We matched the GIAB and PBSV calls with Truvari configured to match SVs within

2000 bp window, with 70% size and sequence similarity[30] and extracted the offsets from the Truvari annotations.

Availability of source code and requirements

Project name: npsv
Project home page: https://github.com/mlinderm/npsv
Operating system(s): Linux
Programming language: Python, C++, BASH
License: MIT
RRID: SCR_020984

Availability of supporting data

Supporting data is available via the GigaScience database [62]. The GIAB SV call set is available in the GIAB FTP repository [50] and the sequencing data for HG002, HG003 and HG004 respectively at [30,52,53,54,55].
The SV-plaudit call set is available in the supplemental materials at [32,51]. The Polaris call sets are available via GitHub [31]. The NA12878 sequencing data is available in the European Nucleotide Archive under project PRJEB3381 [57,58].

Declarations

Abbreviation

ME: Mendelian Error, MER: Mendelian Error Rate, NGS: Next-generation sequencing, RF: Random Forest, SV: Structural variant, SVM: Support vector machine, WGS: Whole genome sequencing

Ethics approval and consent to participate

Not applicable

Consent for publication

Not applicable

Competing interests

The authors declare that they have no competing interests

Funding

Research reported in this publication was supported by an Institutional Development Award (IDeA) from the NIGMS of the NIH under grant number P20GM103449, awards UM1HL098123 and U01HL153009 from the NHLBI of the NIH, and the NSF under Grant No. 1827373. Its contents are solely the responsibility of the authors and do not necessarily represent the official views of NIGMS, NHLBI, NIH or the NSF.

Authors' contributions

MDL, AB and BDG conceived of the project. MDL, CP, MS and WK developed the software and performed the evaluation. MDL, AB and BDG wrote the manuscript. All authors read and approved the final manuscript.

Acknowledgements

References

1. Weischenfeldt J, Symmons O, Spitz F, Korbel JO. Phenotypic impact of genomic structural variation: insights from and for human disease. *Nat Rev Genet*. Nature Publishing Group; 2013; doi: 10.1038/nrg3373.

2. Brandler WM, Antaki D, Gujral M, Noor A, Rosanio G, Chapman TR, et al.. Frequency and Complexity of De Novo Structural Mutation in Autism. *Am J Hum Genet*. 2016; doi: 10.1016/j.ajhg.2016.02.018.

3. Kloosterman WP, Francioli LC, Hormozdiari F, Marschall T, Hehir-Kwa JY, Abdellaoui A, et al.. Characteristics of de novo structural changes in the human genome. *Genome Res*. Cold Spring Harbor Laboratory Press; 2015; doi: 10.1101/gr.185041.114.

4. Guan P, Sung W-K. Structural variation detection using next-generation sequencing data. *Methods*. 2016; doi: 10.1016/j.ymeth.2016.01.020.

5. Kosugi S, Momozawa Y, Liu X, Terao C, Kubo M, Kamatani Y. Comprehensive evaluation of structural variation detection algorithms for whole genome sequencing. *Genome Biol*. BioMed Central; 2019; doi: 10.1186/s13059-019-1720-5.

6. Mahmoud M, Gobet N, Cruz-Dávalos DI, Mounier N, Dessimoz C, Sedlazeck FJ. Structural variant calling: the long and the short of it. *Genome Biol*. 2019; doi: 10.1186/s13059-019-1828-7.

7. Chander V, Gibbs RA, Sedlazeck FJ. Evaluation of computational genotyping of structural variation for clinical diagnoses. *Gigascience*. Narnia; 2019; doi: 10.1093/gigascience/giz110.

8. Lappalainen I, Lopez J, Skipper L, Hefferon T, Spalding JD, Garner J, et al.. dbVar and DGVa: public archives for genomic structural variation. *Nucleic Acids Res*. Narnia; 2012; doi: 10.1093/nar/gks1213.

9. Alkan C, Coe BP, Eichler EE. Genome structural variation discovery and genotyping. *Nat Rev Genet*. Nature Publishing Group; 2011; doi: 10.1038/nrg2958.

10. Sudmant PH, Rausch T, Gardner EJ, Handsaker RE, Abyzov A, Huddleston J, et al.. An integrated map of structural variation in 2,504 human genomes. *Nature*. Nature Publishing Group; 2015; doi: 10.1038/nature15394.

11. Mills RE, Walter K, Stewart C, Handsaker RE, Chen K, Alkan C, et al.. Mapping copy number variation by population-scale genome sequencing. *Nature*. Nature Publishing Group, a division of Macmillan Publishers Limited. All Rights Reserved.; 2011; doi: 10.1038/nature09708.

12. Tattini L, D'Aurizio R, Magi A. Detection of Genomic Structural Variants from Next-Generation Sequencing Data. *Front Bioeng Biotechnol*. 2015; doi: 10.3389/fbioe.2015.00092.

13. Teo SM, Pawitan Y, Ku CS, Chia KS, Salim A. Statistical challenges associated with detecting copy number variations with next-generation sequencing. *Bioinformatics*. 2012; doi: 10.1093/bioinformatics/bts535.

14. Sedlazeck FJ, Rescheneder P, Smolka M, Fang H, Nattestad M, von Haeseler A, et al.. Accurate detection of complex structural variations using single-molecule sequencing. *Nat Methods*. Nature Publishing Group; 2018; doi: 10.1038/s41592-018-0001-7.

15. Huddleston J, Chaisson MJP, Steinberg KM, Warren W, Hoekzema K, Gordon D, et al.. Discovery and genotyping of structural variation from long-read haploid genome sequence data. *Genome Res*. 2017; doi: 10.1101/gr.214007.116.

16. English AC, Salerno WJ, Reid JG. PBHoney: identifying genomic variants via long-read discordance and interrupted mapping. *BMC Bioinformatics*. 2014; doi: 10.1186/1471-2105-15-180.

17. Goodwin S, McPherson JD, McCombie WR. Coming of age: ten years of next-generation sequencing technologies. *Nat Rev Genet*. 2016; doi: 10.1038/nrg.2016.49.

18. Rausch T, Zichner T, Schlattl A, Stütz AM, Benes V, Korbel JO. DELLY: structural variant discovery by integrated paired-end and split-read analysis. *Bioinformatics*. Bioinformatics; 2012; doi: 10.1093/bioinformatics/bts378.

19. Chiang C, Layer RM, Faust GG, Lindberg MR, Rose DB, Garrison EP, et al.. SpeedSeq: ultra-fast personal genome analysis and interpretation. *Nat Methods*. Nature Publishing Group; 2015; doi: 10.1038/nmeth.3505.

20. Spies N, Zook JM, Salit M, Sidow A. svviz: a read viewer for validating structural variants. *Bioinformatics*. 2015; doi: 10.1093/bioinformatics/btv478.

21. Eggertsson HP, Kristmundsdottir S, Beyter D, Jonsson H, Skuladottir A, Hardarson MT, et al.. GraphTyper2 enables population-scale genotyping of structural variation using pangenome graphs. *Nat Commun*. Nature Publishing Group; 2019; doi: 10.1038/s41467-019-13341-9.

22. Antaki D, Brandler WM, Sebat J. SV2: accurate structural variation genotyping and de novo mutation detection from whole genomes. Birol I, editor. *Bioinformatics*. Oxford University Press; 2018; doi: 10.1093/bioinformatics/btx813.

23. Audano PA, Sulovari A, Graves-Lindsay TA, Cantsilieris S, Sorensen M, Welch AE, et al.. Characterizing the Major Structural Variant Alleles of the Human Genome. *Cell*. 2019; doi: https://doi.org/10.1016/j.cell.2018.12.019.

24. Hickey G, Heller D, Monlong J, Sibbesen JA, Sirén J, Eizenga J, et al.. Genotyping structural variants in pangenome graphs using the vg toolkit. *Genome Biol*. BioMed Central; 2020; doi: 10.1186/s13059-020-1941-7.

25. Chen S, Krusche P, Dolzhenko E, Sherman RM, Petrovski R, Schlesinger F, et al.. Paragraph: a graph-based structural variant genotyper for short-read sequence data. *Genome Biol*. BioMed Central; 2019; doi: 10.1186/s13059-019-1909-7.

26. Handsaker RE, Van Doren V, Berman JR, Genovese G, Kashin S, Boettger LM, et al.. Large multiallelic copy number variations in humans. *Nat Genet*. Nature Publishing Group; 2015; doi: 10.1038/ng.3200.

27. Russell SJ, Norvig P, Davis E. Artificial Intelligence: A Modern Approach. Upper Saddle River, NJ: Prentice Hall;

28. Mohiyuddin M, Mu JC, Li J, Bani Asadi N, Gerstein MB, Abyzov A, et al.. MetaSV: an accurate and integrative structural-variant caller for next generation sequencing. *Bioinformatics*. 2015; doi: 10.1093/bioinformatics/btv204.

29. Chu C, Zhang J, Wu Y. GINDEL: accurate genotype calling of insertions and deletions from low coverage population sequence reads. *PLoS One*. PLoS One; 2014; doi: 10.1371/journal.pone.0113324.

30. Zook JM, Hansen NF, Olson ND, Chapman L, Mullikin JC, Xiao C, et al.. A robust benchmark for detection of germline large deletions and insertions. *Nat Biotechnol*. Nat Biotechnol; 2020; doi: 10.1038/s41587-020-0538-8.

31. Polaris. https://github.com/Illumina/Polaris Accessed 2020 Jul 10.

32. Belyeu JR, Nicholas TJ, Pedersen BS, Sasani TA, Havrilla JM, Kravitz SN, et al.. SV-plaudit: A cloud-based framework for manually curating thousands of structural variants. *Gigascience*. 2018; doi: 10.1093/gigascience/giy064.

33. Layer RM, Chiang C, Quinlan AR, Hall IM. LUMPY: a probabilistic framework for structural variant discovery. *Genome Biol*. BioMed Central; 2014; doi: 10.1186/gb-2014-15-6-r84.

34. Chen X, Schulz-Trieglaff O, Shaw R, Barnes B, Schlesinger F, Källberg M, et al.. Manta: rapid detection of structural variants and indels for germline and cancer sequencing applications. *Bioinformatics*. Oxford Academic; 2016; doi: 10.1093/bioinformatics/btv710.

35. pbsv (2020) pbsv https://github.com/PacificBiosciences/pbsv

36. Pedersen BS, Collins RL, Talkowski ME, Quinlan AR. Indexcov: fast coverage quality control for whole-genome sequencing. *Gigascience*. Oxford University Press; 2017; doi: 10.1093/gigascience/gix090.

37.Picard toolkit (2020). Picard Toolkit (Version 2.22.7) http://broadinstitute.github.io/picard

38. Collins RL, Brand H, Karczewski KJ, Zhao X, Alföldi J, Francioli LC, et al.. An open resource of structural variation for medical and population genetics. *bioRxiv*. Cold Spring Harbor Laboratory; 2019; doi: 10.1101/578674.

39. Quinlan AR, Hall IM. BEDTools: a flexible suite of utilities for comparing genomic features. *Bioinformatics*. 2010; doi: 10.1093/bioinformatics/btq033.

40. Li H, Handsaker B, Wysoker A, Fennell T, Ruan J, Homer N, et al.. The Sequence Alignment/Map format and SAMtools. *Bioinformatics*. 2009; doi: 10.1093/bioinformatics/btp352.

41. Huang W, Li L, Myers JR, Marth GT. ART: a next-generation sequencing read simulator. *Bioinformatics*. Oxford University Press; 2012; doi: 10.1093/bioinformatics/btr708.

42. Li H. Aligning sequence reads, clone sequences and assembly contigs with BWA-MEM. 2013;

43. Faust GG, Hall IM. SAMBLASTER: fast duplicate marking and structural variant read extraction. *Bioinformatics*. 2014; doi: 10.1093/bioinformatics/btu314.

44. Chapman B, Kirchner R, Pantano L, Smet M De, Beltrame L, Khotiainsteva T, et al.. bcbio/bcbio-nextgen: v1.2.3. 2020; doi: 10.5281/ZENODO.3743344.

45. Tarasov A, Vilella AJ, Cuppen E, Nijman IJ, Prins P. Sambamba: fast processing of NGS alignment formats. *Bioinformatics*. 2015; doi: 10.1093/bioinformatics/btv098.

46. Parikh H, Mohiyuddin M, Lam HYK, Iyer H, Chen D, Pratt M, et al.. svclassify: a Method To Establish Benchmark Structural Variant Calls. *BMC Genomics*. 2016; doi: 10.1186/s12864-016-2366-2.

47. Wala J, Beroukhim R. SeqLib: a C ++ API for rapid BAM manipulation, sequence alignment and sequence assembly. *Bioinformatics*. Oxford Academic; 2016; doi: 10.1093/bioinformatics/btw741.

48. Pedersen BS, Quinlan AR. Duphold: scalable, depth-based annotation and curation of high-confidence structural variant calls. *Gigascience*. Oxford University Press; 2019; doi: 10.1093/gigascience/giz040.

49. Pedregosa F, Varoquaux G, Gramfort A, Michel V, Thirion B, Grisel O, et al.. Scikit-learn: Machine Learning in Python. *J Mach Learn Res*. 12:2825–302011;

50. : Genome in a Bottle Sequence-Resolved SV Calls v0.6. ftp://ftp-trace.ncbi.nlm.nih.gov/giab/ftp/release/AshkenazimTrio/HG002_NA24385_son/NIST_SV_v0.6 Accessed 2019 Jun 18.

51. Belyeu J, Nicholas T, Pedersen B, Sasani T, Havrilla J, Kravitz S, et al.: Supporting data for "SV-plaudit: A cloud-based framework for manually curating thousands of structural variants." GigaScience Database.

52. Zook JM, Catoe D, McDaniel J, Vang L, Spies N, Sidow A, et al.. Extensive sequencing of seven human genomes to characterize benchmark reference materials. *Sci Data*. Nature Publishing Group; 2016; doi: 10.1038/sdata.2016.25.

53. : Genome in a Bottle HG002 Sequencing Data. ftp://ftp-

trace.ncbi.nlm.nih.gov/ReferenceSamples/giab/data//AshkenazimTrio/HG002_NA24385_son/NIST_HiSeq_HG002_Homogeneity-10953946/HG002_HiSeq300x_fastq/140528_D00360_0018_AH8VC6ADX Accessed 2019 Jun 4.

54. : Genome in a Bottle HG003 Sequencing Data. ftp://ftp-trace.ncbi.nlm.nih.gov/ReferenceSamples/giab/data//AshkenazimTrio/HG003_NA24149_father/NIST_HiSeq_HG003_Homogeneity-12389378/HG003_HiSeq300x_fastq/140721_D00360_0044_AHA66RADXX Accessed 2019 Jun 4.

55. : Genome in a Bottle HG004 Sequencing Data. ftp://ftp-trace.ncbi.nlm.nih.gov/ReferenceSamples/giab/data//AshkenazimTrio/HG004_NA24143_mother/NIST_HiSeq_HG004_Homogeneity-14572558/HG004_HiSeq300x_fastq/140818_D00360_0046_AHA5R5ADXX Accessed 2019 Jun 4.

56. smoove (2020). smoove https://github.com/brentp/smoove.

57. Eberle MA, Fritzilas E, Krusche P, Källberg M, Moore BL, Bekritsky MA, et al.. A reference data set of 5.4 million phased human variants validated by genetic inheritance from sequencing a three-generation 17-member pedigree. *Genome Res*. Cold Spring Harbor Laboratory Press; 2017; doi: 10.1101/gr.210500.116.

58. : Whole genome sequencing and variant calls for the Coriell CEPH/UTAH 1463 family to create a “platinum” standard comprehensive set for variant calling improvement. ftp://ftp.sra.ebi.ac.uk/vol1/fastq/ERR194/ERR194147/ERR194147.fastq.gz Accessed 2018 May 29.

59. Truvari (2020) Truvari https://github.com/spiralgenetics/truvari.

60. Truvari fork. https://github.com/mlinderm/truvari/tree/genotype_stats

61. Danecek P, Bonfield JK, Liddle J, Marshall J, Ohan V, Pollard MO, et al.. Twelve years of SAMtools and BCFtools. *Gigascience*. Gigascience; 2021; doi: 10.1093/gigascience/giab008.

62. Linderman M, Paudyal C, Shakeel M, Kelley W, Bashir A, Gelb B: Supporting data for “NPSV: A simulation-driven approach to genotyping structural variants in whole genome sequencing data.” GigaScience Database. http://dx.doi.org/10.5524/100908 (2021). Accessed 2021 Jun 1.

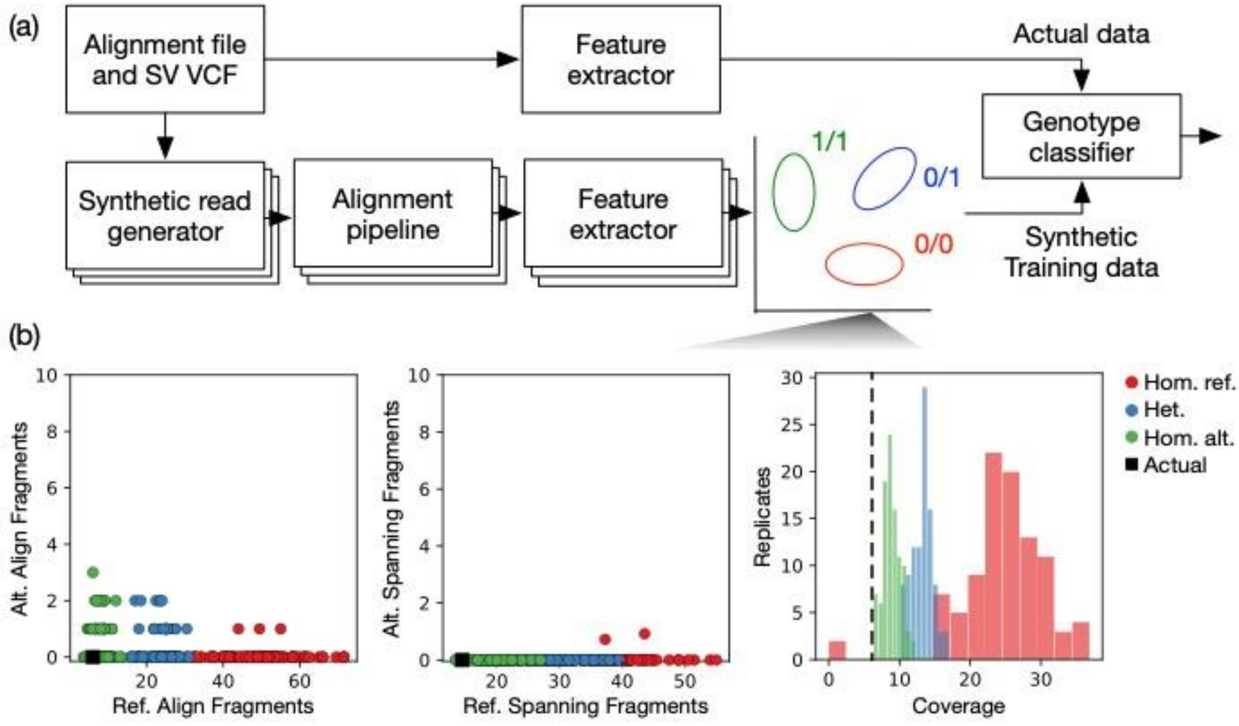

Figure 1: NPSV dataflow and example SV evidence. (a) NPSV dataflow showing the matched training and prediction pipelines. For each putative SV and genotype, NPSV generates one or more simulated replicates. This simulated data, shown in in the schematic as red, blue and green clusters for homozygous reference, heterozygous and homozygous alternate genotypes respectively, are used to train sample- and variant-specific classifiers for predicting the SV genotype. (b) Synthetic training data (colored circles/bars) and actual data (black square/line) for a homozygous alternate 822 bp deletion in HG002. This SV is the deletion of one copy of a repeat and as a result of the repetitive genomic context, no fragments were uniquely re-aligned to the SV's alternate allele and no alternate spanning fragments were identified. The actual data is consistent with the simulated homozygous alternate data and not a homozygous reference genotype as might be expected from the absence of alternate allele re-alignments. This SV is successfully genotyped as homozygous alternate by NPSV when building a variant-specific classifier.

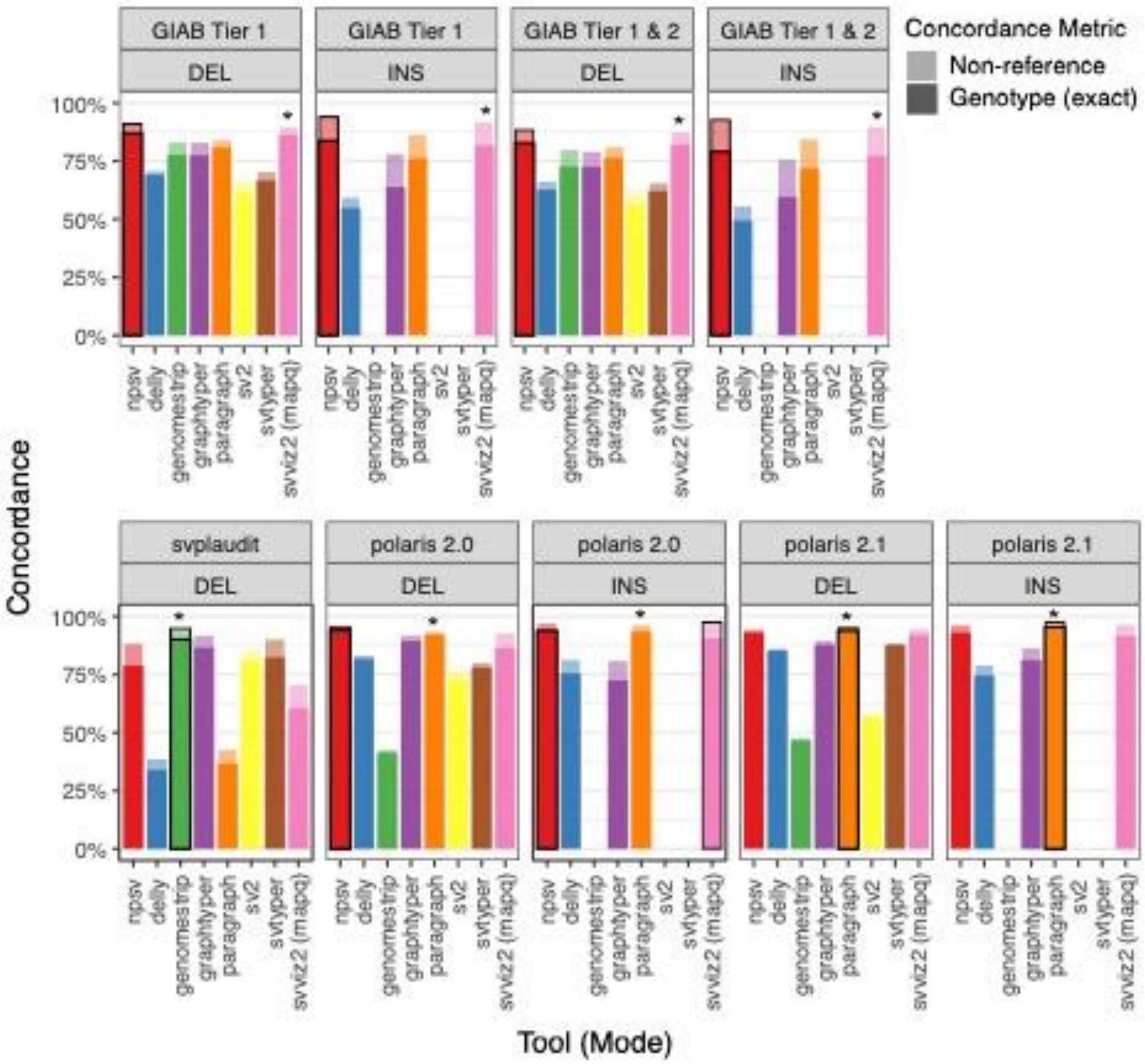

Figure 2: Genotyping accuracy for HG002 and NA12878 SVs. (a) Genotype concordance and non-reference concordance (presence or absence) for GIAB SVs (including "LongReadHomRef" SVs where "long reads supported homozygous reference for all individuals") in high-confidence tier 1 regions and the tier 1 regions and lower-confidence tier 2 SVs combined. (b) Concordance for NA12878 call sets. The NPSV accuracy is the mean of 10 runs. The best concordance is indicated with a black outline. The * shows tools used in the construction of that call set.

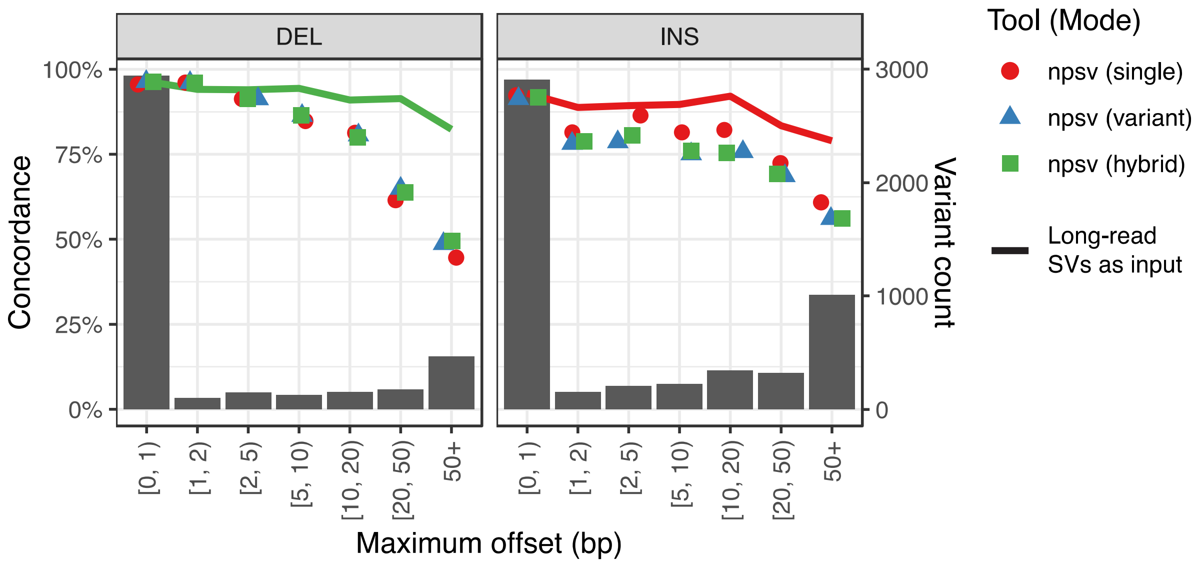

Figure 3: Genotype concordance for GIAB SVs with offset breakpoints. Genotype concordance for GIAB variant-only SVs in tier 1 regions grouped by the maximum offset between the GIAB breakpoints and the breakpoints for the corresponding SV called with PBSV in PacBio long-read sequencing data. The line shows the concordance when using the PBSV SVs as the input to NPSV running the default genotyoping mode ("hybrid" for deletions, "single" for insertions). The background bar chart shows the underlying distribution of offsets. The same analysis for select comparison tools is included in Supplemental Figure S5.

Table 1: Genotyping accuracy with discovery SVs as the input to SV genotyping and GIAB SVs in tier 1 regions as the truth set. Concordance is calculated for the subset of SVs successfully identified by the discovery tool.

			Caller Genotyping		NPSV Genotyper	
**Caller**	**Type**	**Discovery Recall**	**Concordance**	**Non-reference Concordance**	**Concordance**	**Non-reference Concordance**
lumpy	DEL	30.5%	82.1%	87.2%	88.5%	92.7%
manta	DEL	67.9%	90.1%	91.8%	92.2%	93.6%
manta	INS	25.2%	87.3%	93.5%	89.1%	93.7%

Table 2: Mendelian error rate (MER) and Mendelian error (ME) breakdown for GIAB autosomal SVs in tier 1 regions. NPSV default mode is shaded.

	DEL				INS			
**Tool**	**MER**	**Het. *de novo***	**Hom. *de novo***	**Other**	**MER**	**Het. *de novo***	**Hom. *de novo***	**Other**
npsv (single)	3.60% (231/6416)	99	7	125	4.64% (291/6269)	58	6	227
npsv (variant)	3.09% (198/6416)	111	1	86	5.14% (322/6269)	74	4	244
npsv (hybrid)	3.21% (206/6416)	106	1	99	5.10% (320/6269)	65	6	249
delly	1.66% (92/5535)	50	2	40	1.92% (78/4059)	26	0	52
genomestrip	2.02% (128/6337)	81	3	44				
graphtyper	5.53% (353/6386)	109	16	228	10.13% (608/6004)	86	27	495
paragraph	2.76% (175/6351)	85	2	88	5.42% (329/6067)	91	4	234
sv2	8.80% (536/6089)	129	47	360				
svtyper	2.28% (145/6349)	79	6	60				
svviz2 (mapq)	2.46% (158/6416)	94	3	61	3.32% (208/6269)	75	3	130

Figure 1

Click here to access/download;Figure;Figure1.pdf

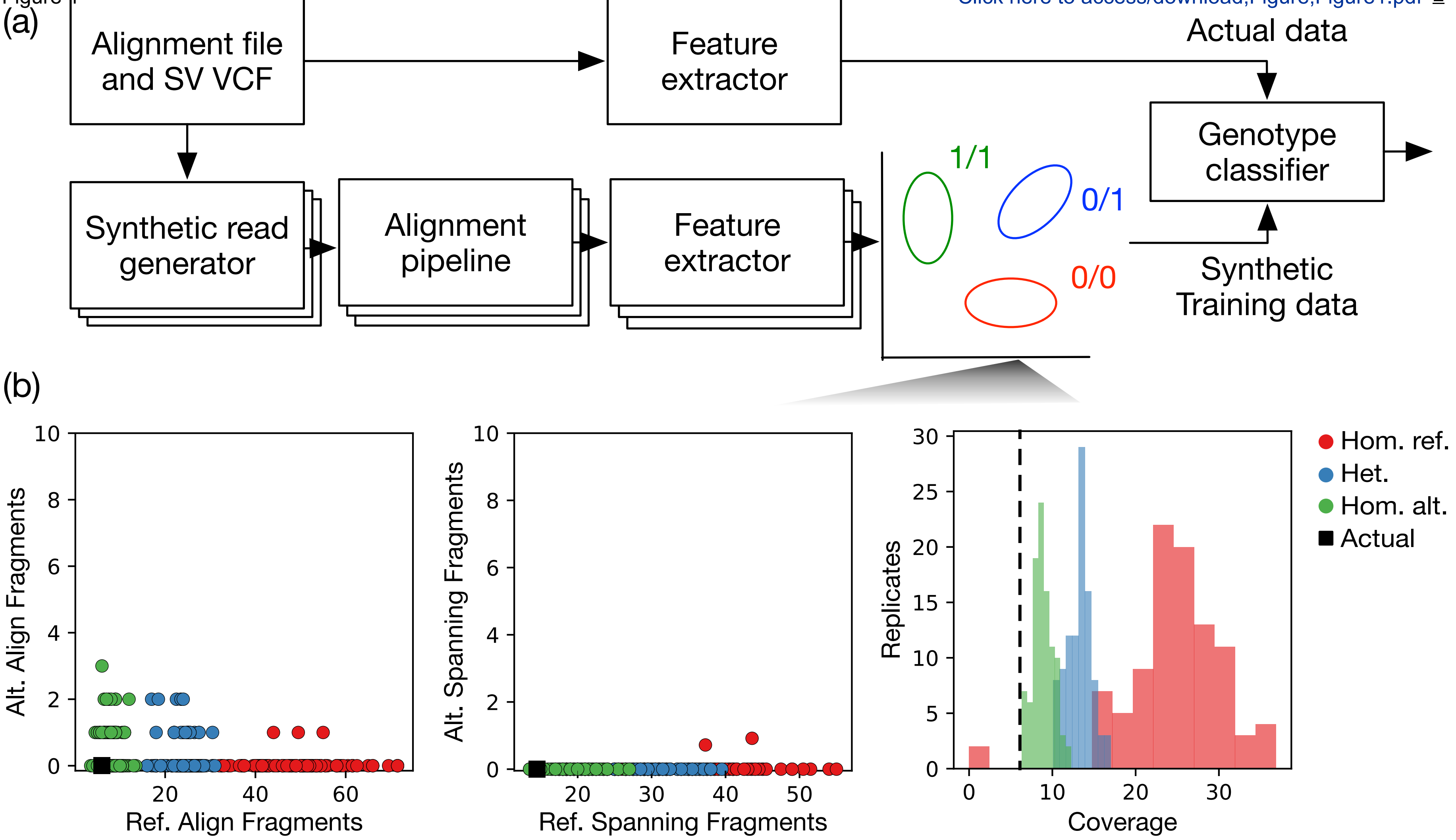

Figure 2

Click here to access/download;Figure;Figure2.pdf

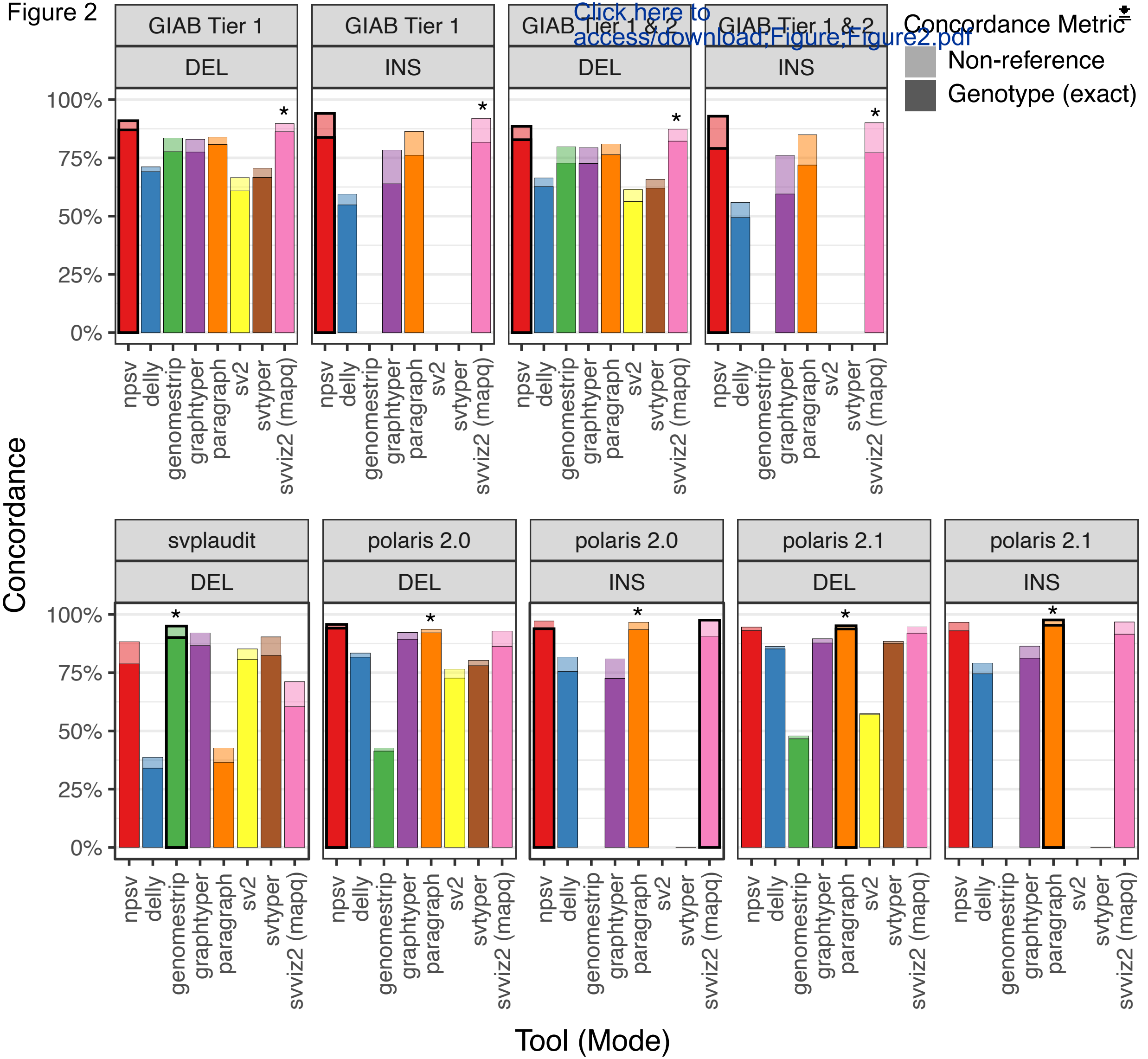

Figure 3

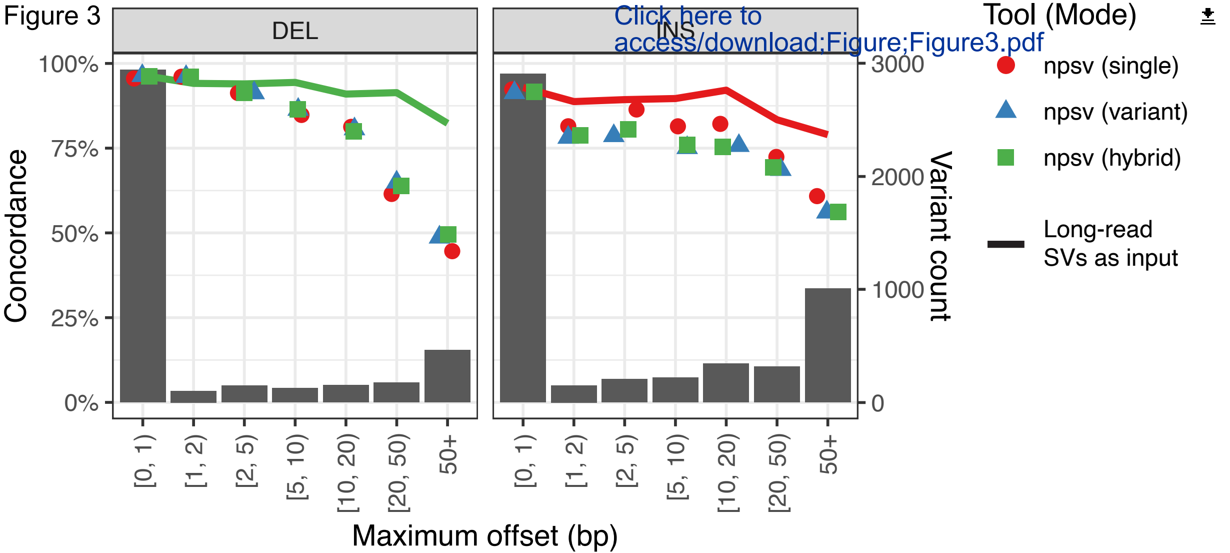

DEL
INS
Click here to access/download;Figure;Figure3.pdf
Concordance
100%
75%
50%
25%
0%
Variant count
3000
2000
1000
0
[0, 1)
[1, 2)
[2, 5)
[5, 10)
[10, 20)
[20, 50)
50+
Maximum offset (bp)
Tool (Mode)
npsv (single)
npsv (variant)
npsv (hybrid)
Long-read SVs as input

Click here to access/download
Supplementary Material
NPSVManuscriptSupplemental.pdf
