# Supplementary material for: NPSV: A simulation-driven approach to genotyping structural variants in whole-genome sequencing data
Source: Gigascience. 2021 Jul 1;10(7):giab046. doi: 10.1093/gigascience/giab046 (PMC8246072; doi:10.1093/gigascience/giab046)
Supplement: giab046_Supplemental_File [file giab046_supplemental_file.pdf]

NPSV: A simulation-driven approach to genotyping structural variants in whole genome sequencing data

Supplemental Methods and Results

Michael D. Linderman,[1*] Crystal Paudyal,[1] Musab Shakeel,[1] William Kelley,[1] Ali Bashir[2†], Bruce D. Gelb[3†]

[1]Department of Computer Science, Middlebury College, Middlebury, VT, USA
[2]Google, Mountain View, CA, USA
[3]Mindich Child Health and Development Institute and the Departments of Pediatrics and Genetics and Genomic Sciences, Icahn School of Medicine at Mount Sinai, New York, NY, USA

[†]These authors contributed equally

*Corresponding Author:

Michael D. Linderman
mlinderman@middlebury.edu
Department of Computer Science
Middlebury College
14 Old Chapel Road
Middlebury, VT 05753
(802) 443-5737

Table of Contents

1 Supplemental Methods **3**

1.1 Feature Extraction *3*

1.2 Concordance Analysis *4*

1.3 Selecting Alternate Variant Representations *5*

2 Supplemental Results **7**

3 References **20**

1 Supplemental Methods

1.1 Feature Extraction

Table S1 summarizes the features extracted from the real and simulated data during genotyping. The features are described in more detail below.

Table S1: Features extracted from real and simulated NGS data. The SVM and RF columns show the features used with the respective classifiers. Coverage features are not extracted for insertions.

Feature	SVM	RF	Description
SVLEN	✓	✓	Difference in length between reference and alternate alleles
REF_READ_REL	✓		Fraction of reads realigned to reference and alternates alleles via BWA. Adapted from svviz2[1].
ALT_READ_REL	✓	✓	
REF_WEIGHTED_SPAN_REL	✓		Fraction of fragments spanning reference and alternate breakpoints weighted by insert-size probability. Adapted from SVTyper[2].
ALT_WEIGHTED_SPAN_REL	✓	✓	
INSERT_LOWER	✓	✓	Fraction of fragments spanning entire event with insert sizes with Z-scores < -1.5 and > 1.5. Adapted from SMRT-SV2[1].
INSERT_UPPER	✓	✓	
CLIP_PRIMARY	✓	✓	Fraction of reads overlapping breakpoints that are clipped. Adapted from SMRT-SV2[3].
DHFC	✓		Mean coverage within the event relative to mean coverage over the chromosome. Adapted from duphold[4].
DHBFC	✓		Mean coverage within the event relative to mean coverage in regions with the same GC fraction. Adapted from duphold[4].
DHFFC	✓	✓	Mean coverage within the event relative to coverage of the flanking regions. Adapted from duphold[4].
PROB_HOMREF	✓	✓	Probability of a homozygous reference, heterozygous and homozygous alternate genotypes assuming a binomial model for allelic depth and probabilities for the alternate allele of 0.05, 0.5, and 0.95 respectively. Adapted from SVTyper2], svviz2[1] and SMRT-SV2[3].
PROB_HET	✓	✓	
PROB_HOMALT	✓	✓	

The realignment features (REF_READ_REL, ALT_READ_REL) are the relative fractions of fragments realigned to the reference and alternate breakpoints with BWA (adapted from

svviz2[1]). Read fragments are first extracted from the original alignments in a region around the putative SV. The extracted fragments are realigned with BWA to both the reference and alternate haplotypes for a putative SV with each haplotype treated as a separate “reference” genome.

The deletion and insertion SVs have three breakpoints (reference left and right breakpoints and a single alternate breakpoint for deletions, a single reference breakpoint and two alternate breakpoints for insertions). A fragment is assigned to an allele breakpoint if the fragment overlaps that breakpoint, the overlapping alignment has the best score of all alignments, and that score is greater than the best alignment score for the other allele by a settable threshold. The alignment score is a product the individual read alignment probabilities and the insert size probability. Breakpoint overlap is determined by the entire fragment span, i.e., the fragment can “straddle” the breakpoint.

A single fragment can be aligned to both of the breakpoints of an allele (but not both alleles). The final read counts for the reference and alternate alleles are calculated by normalizing the counts of overlapping fragments by the number of breakpoints for that allele.

The read pair features (REF_WEIGHTED_SPAN_REL, ALT_WEIGHTED_SPAN_REL) are weighted relative fractions of reads spanning the SV breakpoints in the original alignments (adapted from SVTyper[2]). For deletions, fragments count towards the reference and alternate allele if they straddle the entire event, and just the reference allele if they only straddle one of the two reference breakpoints. The counts are weighted by the probability of observing that insert size given the insert size distribution for the sample assuming the reference or alternate allele.

The CLIP_PRIMARY feature is the fraction of reads in the original alignments overlapping the breakpoint(s) that contain soft/hard clipped bases (with the length of clipping above a settable threshold) relative to the total number of reads overlapping the breakpoint (adapted from SMRT-SV2[3]). For deletions the clipping must occur within the event, for insertions the clipping occurs on either side of the breakpoint.

The PROB_HOMREF, PROB_HET, and PROB_HOMALT features are calculated from the realigned read counts (that are used to compute REF_READ_REL, ALT_READ_REL) as described in Table S1.

1.2 Concordance Analysis

Figure S1 shows how the accuracy metrics, genotype concordance and non-reference concordance, are calculated from the contingency table. In most evaluations the truth set SVs are used as the genotyping input and thus the test and truth sets should have the same SVs. The truth sets are filtered to only include genotyped SVs. Test “./.” includes SV dropped by the genotyper and “no genotypes”, i.e., an explicit “./.” genotype (panels a-b). When genotyping SVs produced by an SV discovery tool (the “discovery” context), the “./.” could include both missing genotypes and missing SV calls. To focus on genotyping accuracy, SVs that were not detected by the discovery tool were excluded from the concordance calculation (panels b-c). Thus “Test ./.” represents an SV that is successfully called but not genotyped. The discovery analysis is performed on SVs in GIAB Tier 1 regions; we assume any extra SVs, the “Truth ./.” entries, are false positives[5], and thus should be genotyped as homozygous reference.

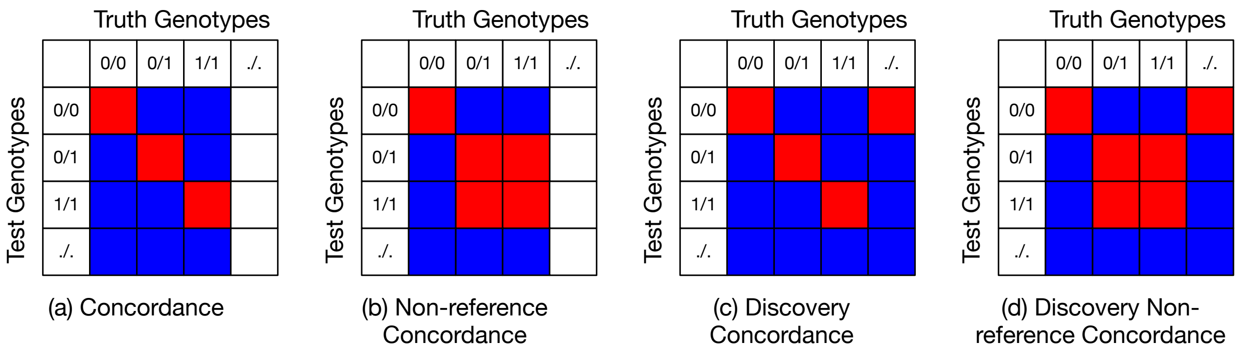

Figure S1: Accuracy metrics calculation from the contingency table. Concordance calculated as the ratio of red squares to all filled squares (red and blue). In the discovery context, SVs that are not called by the discovery tool are excluded (i.e., a test genotype of "./." represents an SV that is successfully called but explicitly genotyped as "./.").

The contingency table is generated by Truvari[6]. In the genotyping context, Truvari is configured with "`--sizemax 15000000 -s 50 -S 30 --pctsim 0 --refdist 20 --pctovl 0.6`"[4], and in the discovery context with "`--sizemax 15000000 -s 50 -S 30 –pctsim 0 –pctsize 0.7 --refdist 2000`"[5].

1.3 Selecting Alternate Variant Representations

Figure S2 shows the dataflow for proposing and selecting alternate SV descriptions.

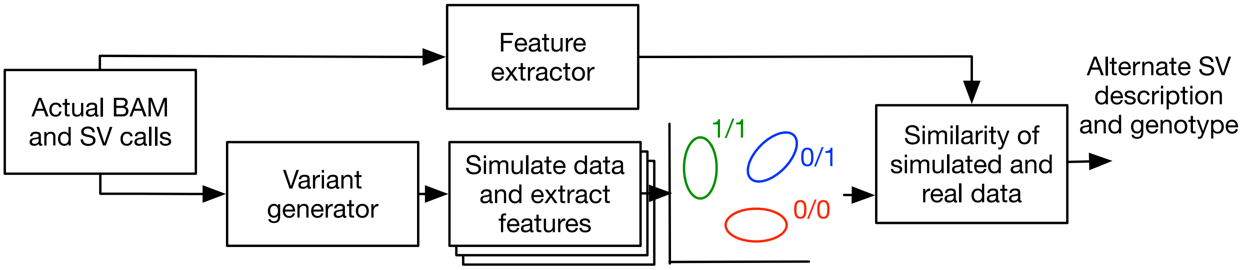

Figure S2: Dataflow for proposing and selecting alternate variant SV descriptions.

For putative SV deletions overlapping a tandem repeat (as determined by intersection with the UCSC Genome Browser simple repeats track[7]), the variant generator identifies up to 10 alternate alignments of the nearest integer number of repeat copies (e.g., if the original SV length was equivalent to 1.9 copies of the consensus repeat sequence, a sequence of 2 copies in length would be aligned). Alternate alignments are identified and ranked by the hamming distance. These alternate alignments are converted to SV deletions.

The original SV and the proposed alternate SVs are genotyped in "variant" mode. We calculate the Mahalanobis distance (D_M) between the SV features in the actual data and the simulated data for each genotype. The hypothesis is that the actual data will be closest to the simulated data (smallest D_M) for the true SV description and genotype. After genotyping all SV representations, we select the genotype from an alternate SV representation if: 1) the closest D_M for the alternate SV is a non-reference genotype (i.e., heterozygous or homozygous alternate), and 2) its closest D_M is the minimum among all D_Ms, including for the original SV description. By default, the selection rule is applied once per-block of overlapping SVs, for the SV with the smallest non-

reference D_M. All other SVs in the block that would be updated are set to homozygous reference (reflecting the hypothesis that there is one underlying SV in the block).

The workflow in Figure S2 is implemented with additional pre- and post-processing steps wrapped around the NPSV genotyper. The pre-processing step (*propose*) generates alternate SV representations as described above. All of the SVs, i.e., both the original SV and the proposed alternated SVs, are genotyped with NPSV in variant mode. The post-processing step (*refine*) selects the single best SV representation for each original SV according to the algorithm described above.

2 Supplemental Results

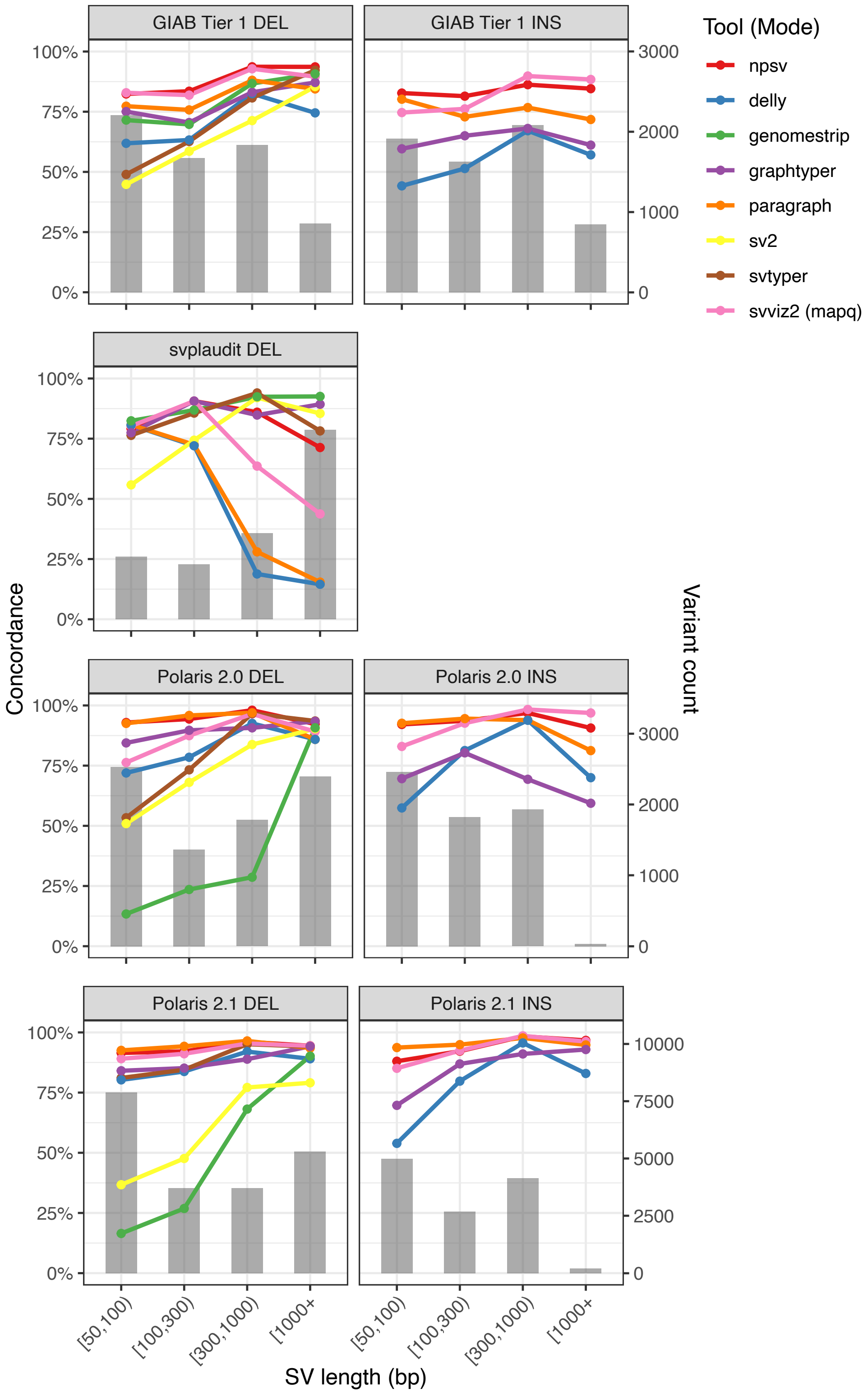

Figure S3: Genotype concordance for different SV lengths (SVLEN). The background bar chart shows the underlying distribution of SV lengths in the call sets.

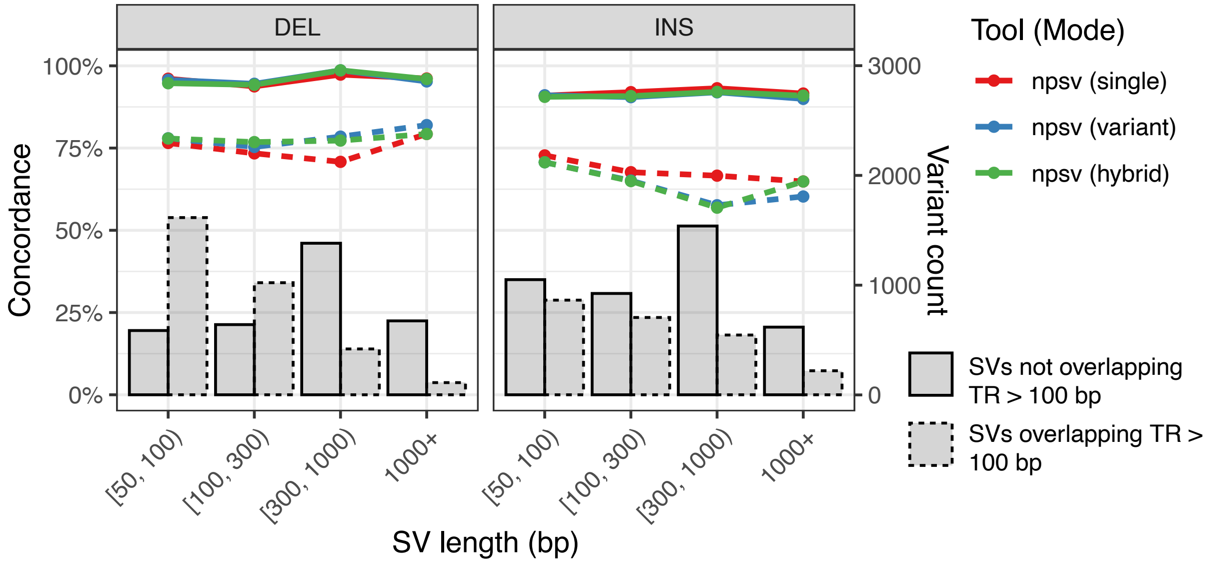

Figure S4: Genotype concordance for GIAB SVs in tier 1 regions grouped by SV length (SVLEN) and TR overlap. The dashed line shows the concordance for SVs annotated as overlapping a TR > 100 bp, the solid line for SVs that do not. The default NPSV configuration uses hybrid mode for deletions and the single model for insertions. The background bar chart shows the underlying distribution of SV lengths and TR overlap.

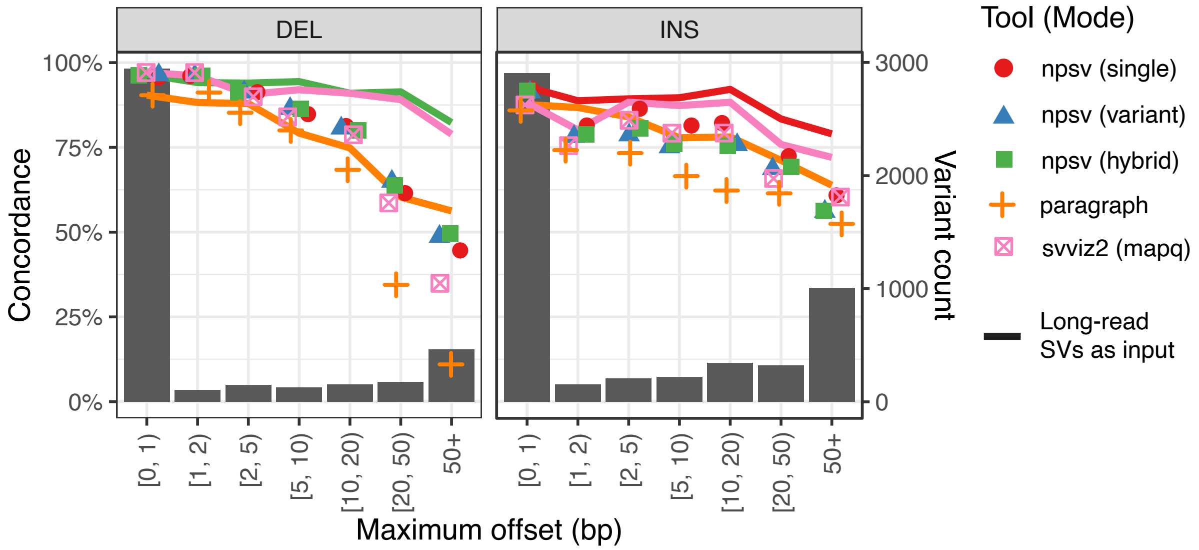

Figure S5: Genotype concordance for GIAB SVs with offset breakpoints. Genotype concordance for GIAB variant-only SVs in tier 1 regions grouped by the maximum offset between the GIAB breakpoints and the breakpoints for the corresponding SV called with PBSV in PacBio long-read sequencing data. The line shows the concordance when using the PBSV SVs as the input to the genotyper. The background bar chart shows the underlying distribution of offsets. This expands Figure 3 in the main text with representative comparison tools.

Table S2: Counts of genotypes for SV truth sets in concordance evaluation.

Dataset			Hom. Ref.	Het.	Hom. Alt.
GIAB	Tier 1	DEL	2245	2561	1643
		INS	1018	2634	2810
	Tier 1 and 2	DEL	2905	3435	2030
		INS	1136	3503	3774
SV-plaudit		DEL	0	778	365
Polaris 2.0		DEL	3811	2449	1813
		INS	1619	2075	2552
Polaris 2.1		DEL	16433	2663	1514
		INS	6281	2452	3295

Table S3: Genotyping accuracy for GIAB SVs. Best concordance is bolded. The mean (SD) accuracy for 10 runs is shown for NPSV. NPSV default mode is shaded.

	**Tier 1**				**Tier 1 and 2**			
	**DEL**		**INS**		**DEL**		**INS**	
	**Genotype Concordance**	**Non-reference Concordance**	**Genotype Concordance**	**Non-reference Concordance**	**Genotype Concordance**	**Non-reference Concordance**	**Genotype Concordance**	**Non-reference Concordance**
npsv (single)	0.857 (0.0016)	0.898 (0.0017)	**0.838 (0.0011)**	**0.941 (0.0014)**	0.812 (0.0015)	0.870 (0.0017)	0.791 (0.0011)	0.929 (0.0014)
npsv (variant)	0.869 (0.0014)	0.909 (0.0011)	0.818 (0.0016)	0.923 (0.0008)	0.827 (0.0013)	**0.886 (0.0007)**	0.765 (0.0011)	0.904 (0.0010)
npsv (hybrid)	**0.870 (0.0015)**	**0.910 (0.0012)**	0.821 (0.0008)	0.925 (0.0011)	**0.828 (0.0013)**	0.885 (0.0012)	0.770 (0.0009)	0.908 (0.0010)
delly	0.691	0.711	0.548	0.595	0.627	0.664	0.494	0.559
genomestrip	0.776	0.836			0.728	0.798		
graphtyper	0.775	0.830	0.639	0.784	0.726	0.794	0.595	0.761
paragraph	0.808	0.840	0.761	0.864	0.763	0.810	0.719	0.849
sv2	0.608	0.665			0.562	0.614		
svtyper	0.666	0.706			0.620	0.658		
svviz2 (mapq)	0.862	0.897	0.817	0.920	0.822	0.874	0.772	0.901

Table S4: Recall, precision and F1 for genotyping homozygous reference vs. non-reference GIAB SVs. The mean (SD) for 10 runs is shown for NPSV. Best result is bolded. NPSV default mode is shaded.

	Tier 1						Tier 1 and 2					
	DEL			INS			DEL			INS		
	**Recall**	**Precision**	**F1**	**Recall**	**Precision**	**F1**	**Recall**	**Precision**	**F1**	**Recall**	**Precision**	**F1**
npsv (single)	0.933 (0.0028)	0.912 (0.0024)	0.923 (0.0013)	**0.952 (0.0018)**	0.978 (0.0005)	**0.964 (0.0009)**	0.916 (0.0031)	0.889 (0.0021)	0.902 (0.0014)	**0.944 (0.0020)**	0.973 (0.0005)	**0.958 (0.0009)**
npsv (variant)	0.940 (0.0011)	0.923 (0.0009)	0.931 (0.0008)	0.926 (0.0008)	0.981 (0.0006)	0.953 (0.0005)	0.924 (0.0011)	0.903 (0.0005)	**0.914 (0.0006)**	0.912 (0.0011)	0.976 (0.0005)	0.943 (0.0006)
npsv (hybrid)	**0.941 (0.0013)**	0.922 (0.0011)	**0.931 (0.0009)**	0.929 (0.0012)	0.981 (0.0006)	0.954 (0.0007)	**0.924 (0.0013)**	0.902 (0.0010)	0.913 (0.0009)	0.917 (0.0012)	0.976 (0.0005)	0.945 (0.0006)
delly	0.722	0.894	0.799	0.587	0.986	0.736	0.683	0.872	0.766	0.553	0.981	0.708
genomestrip	0.853	0.893	0.872				0.804	0.879	0.840			
graphtyper	0.858	0.881	0.869	0.787	0.952	0.862	0.808	0.870	0.838	0.764	0.955	0.849
paragraph	0.821	0.929	0.872	0.860	0.979	0.916	0.790	0.910	0.846	0.850	0.976	0.909
sv2	0.537	0.956	0.687				0.469	0.938	0.626			
svtyper	0.561	**0.982**	0.714				0.490	**0.976**	0.653			
svviz2 (mapq)	0.920	0.922	0.921	0.913	**0.991**	0.950	0.901	0.905	0.903	0.897	**0.987**	0.940

Table S5: Genotyping accuracy for NA12878 call sets. Best concordance is bolded. The mean (SD) accuracy for 10 runs is shown for NPSV. NPSV default mode is shaded.

	**SV-plaudit**		**Polaris 2.0 (GRCh37)**				**Polaris 2.1 (GRCh38)**			
	**DEL**		**DEL**		**INS**		**DEL**		**INS**	
	**Genotype Concordance**	**Non-reference Concordance**	**Genotype Concordance**	**Non-reference Concordance**	**Genotype Concordance**	**Non-reference Concordance**	**Genotype Concordance**	**Non-reference Concordance**	**Genotype Concordance**	**Non-reference Concordance**
npsv (single)	0.792 (0.0026)	0.884 (0.0035)	0.941 (0.0008)	0.956 (0.0006)	**0.939 (0.0005)**	0.972 (0.0005)	0.922 (0.0023)	0.936 (0.0022)	0.929 (0.0011)	0.966 (0.0010)
npsv (variant)	0.759 (0.0025)	0.859 (0.0027)	0.939 (0.0006)	0.955 (0.0006)	0.933 (0.0018)	0.973 (0.0009)	0.930 (0.0007)	0.946 (0.0005)	0.929 (0.0009)	0.969 (0.0008)
npsv (hybrid)	0.788 (0.0035)	0.883 (0.0026)	**0.941 (0.0008)**	**0.957 (0.0008)**	0.933 (0.0015)	0.973 (0.0006)	0.931 (0.0012)	0.946 (0.0011)	0.929 (0.0010)	0.969 (0.0006)
delly	0.340	0.387	0.817	0.834	0.755	0.817	0.852	0.863	0.745	0.792
genomestrip	**0.901**	**0.950**	0.414	0.427			0.466	0.479		
graphtyper	0.866	0.921	0.893	0.922	0.726	0.810	0.877	0.896	0.812	0.864
paragraph	0.366	0.427	0.921	0.936	0.935	0.967	**0.938**	**0.951**	**0.954**	**0.976**
sv2	0.807	0.852	0.727	0.766			0.569	0.575		
svtyper	0.824	0.904	0.780	0.803			0.876	0.885		
svviz2 (mapq)	0.605	0.711	0.863	0.929	0.906	**0.976**	0.920	0.947	0.915	0.968

Table S6: Recall, precision and F1 for genotyping homozygous reference vs. non-reference for NA12878 call sets. SV-plaudit is not included due to absence of "true negative" homozygous reference genotypes. The mean (SD) for 10 runs is shown for NPSV. Best result is bolded. NPSV default mode is shaded.

	Polaris 2.0 (GRCh37)						Polaris 2.1 (GRCh38)					
	DEL			INS			DEL			INS		
	Recall	Precision	F1	Recall	Precision	F1	Recall	Precision	F1	Recall	Precision	F1
npsv (single)	0.958 (0.0009)	0.959 (0.0010)	0.959 (0.0006)	**0.990 (0.0007)**	0.972 (0.0008)	0.981 (0.0003)	**0.949 (0.0025)**	0.782 (0.0076)	0.857 (0.0041)	0.985 (0.0007)	0.945 (0.0023)	0.965 (0.0010)
npsv (variant)	0.956 (0.0009)	0.958 (0.0008)	0.957 (0.0005)	0.982 (0.0011)	0.981 (0.0007)	0.982 (0.0006)	0.935 (0.0013)	0.824 (0.0015)	0.876 (0.0011)	0.972 (0.0016)	0.964 (0.0009)	0.968 (0.0009)
npsv (hybrid)	**0.961 (0.0011)**	0.958 (0.0008)	**0.960 (0.0008)**	0.982 (0.0007)	0.981 (0.0007)	0.982 (0.0004)	0.939 (0.0018)	0.820 (0.0038)	0.875 (0.0023)	0.973 (0.0011)	0.964 (0.0009)	0.968 (0.0007)
delly	0.755	0.965	0.847	0.853	0.976	0.911	0.669	0.792	0.725	0.800	0.951	0.869
genomestrip	0.296	0.955	0.452				0.361	0.771	0.491			
graphtyper	0.927	0.926	0.927	0.800	0.936	0.862	0.866	0.695	0.771	0.863	0.857	0.860
paragraph	0.890	0.989	0.937	0.970	**0.987**	0.979	0.886	0.877	**0.882**	0.975	**0.977**	**0.976**
sv2	0.631	0.971	0.765				0.339	0.898	0.492			
svtyper	0.636	**0.990**	0.774				0.468	**0.948**	0.627			
svviz2 (mapq)	0.890	0.973	0.930	0.981	0.986	**0.984**	0.865	0.871	0.868	0.958	0.974	0.966

Table S7: Genotype concordance for GIAB SVs in tier 1 regions grouped by SVLEN

SVLEN	DEL		INS	
	NPSV (single)	NSPV (variant)	NPSV (single)	NPSV (variant)
[50, 100)	0.817	0.827	0.827	0.819
[100, 300)	0.812	0.827	0.815	0.795
[300, 1000)	0.912	0.939	0.862	0.830
1000+	0.938	0.934	0.846	0.822

Table S8: Recall, precision and F1 for genotyping homozygous reference vs. non-reference with discovery SVs as the input to SV genotyping and GIAB SVs in tier 1 regions as the truth set. To focus on genotyping accuracy, SVs that were not detected ("no-calls") were excluded from the false negative count.

Caller	Type	Genotyper	TP	FP	FN	Recall	Precision	F1
Lumpy	DEL	Caller	1275	261	9	0.993	0.830	0.904
		NPSV	1258	128	26	0.980	0.908	0.942
Manta	DEL	Caller	2785	271	70	0.975	0.911	0.942
		NPSV	2834	244	21	0.993	0.921	0.955
	INS	Caller	1353	89	18	0.987	0.938	0.962
		NPSV	1362	95	9	0.993	0.935	0.963

Table S9: Genotyping Mendelian error SVs reported in Zook et al.[5] Trio genotyping of mendelian error SVs reported in GIAB HG002 trio. For the two ME deletions, we report the minimum genotyper-computed GQ among the trio and the rank (in descending order) of that GQ among all ME deletions. Zook et al. described these SVs as “a likely *de novo* deletion in HG002” and “a deletion in the T cell receptor alpha locus known to undergo somatic rearrangement”, respectively.

**Zook et al. manual review**		**NPSV (single)**			**NPSV (variant)**			**NPSV (hybrid)**			**svviz2 (mapq)**		
**Variant**	***de novo***	***de novo***	**Min GQ**	**GQ Rank**	***de novo***	**Min GQ**	**GQ Rank**	***de novo***	**Min GQ**	**GQ Rank**	***de novo***	**Min GQ**	**GQ Rank**
17:51417826-51417932	✓	✓	22	3	✓	99	1	✓	99	1	✓	49	3
14:22918114-22982920	✓	✓	16	16	✓	99	2	✓	15	4	✓	99	1

Table S10: Genotyping accuracy and recall, precision and F1 for genotyping homozygous reference vs. non-reference SVs for original GIAB SVs and proposed alternative SVs. Proposed SVs are generated with the algorithm shown **Figure S2**.

	Tier 1 Deletions					Tier 1 and 2 Deletions				
	**Genotype Concordance**	**Non-reference Concordance**	**Recall**	**Precision**	**F1**	**Genotype Concordance**	**Non-reference Concordance**	**Recall**	**Precision**	**F1**
Original SVs	0.872	0.911	0.941	0.924	0.933	0.829	0.886	0.925	0.903	0.914
Proposed SVs	0.878	0.920	0.965	0.917	0.940	0.837	0.900	0.957	0.897	0.926

Table S11: Execution time and memory usage for genotyping GIAB SVs in the HG002 sample. All tools were run on a 36-core cluster node with dual 18-core Intel Xeon 6140 2.3 GHz CPUs. Wall clock runtime determined by the time utility. Memory usage was determined from the maximum resident set size reported by the SLURM cluster manager. Parallelization across variants was used if available. For NPSV, runtime with the fallback preprocessing step (instead of using pre-computed metrics, which requires less than one minute) is reported in parentheses. Tools are separated by the input SV set (not all tools support the GIAB INS SVs). Some of the tools are parallelized across samples, a capability not exploited in this single sample setting. This evaluation only uses parallelization built into the tools; however, otherwise single threaded tools could be parallelized by implementing a separate wrapper that splits the call set into independent inputs.

**Tool**	**Threads**	**Wall Clock Time (Min)**	**Memory (GB)**
DEL and INS SVs (16871 SVs)			
npsv (default)	36	1263 (1407)	22.1
npsv (single)	36	53.3 (198)	17.7
npsv (variant)	36	2373 (2517)	39.0
npsv (hybrid)	36	1871 (2015)	24.3
delly	1	25.5	8.1
graphtyper	1	875	5.3
paragraph	36	15.9	5.2
svviz2 (mapq)	1	749	6.7
DEL SVs only (8439 SVs)			
genomestrip	1	190*	1.7
sv2	1	34.5	0.56
svtyper	1	2.1	0.53

* Sum of preprocessing and genotyping execution time

3 References

1. Spies N, Zook JM, Salit M, Sidow A. svviz: a read viewer for validating structural variants. *Bioinformatics*. 2015; doi: 10.1093/bioinformatics/btv478.

2. Chiang C, Layer RM, Faust GG, Lindberg MR, Rose DB, Garrison EP, et al.. SpeedSeq: ultra-fast personal genome analysis and interpretation. *Nat Methods*. Nature Publishing Group; 2015; doi: 10.1038/nmeth.3505.

3. Audano PA, Sulovari A, Graves-Lindsay TA, Cantsilieris S, Sorensen M, Welch AE, et al.. Characterizing the Major Structural Variant Alleles of the Human Genome. *Cell*. 2019; doi: https://doi.org/10.1016/j.cell.2018.12.019.

4. Pedersen BS, Quinlan AR. Duphold: scalable, depth-based annotation and curation of high-confidence structural variant calls. *Gigascience*. Oxford University Press; 2019; doi: 10.1093/gigascience/giz040.

5. Zook JM, Hansen NF, Olson ND, Chapman L, Mullikin JC, Xiao C, et al.. A robust benchmark for detection of germline large deletions and insertions. *Nat Biotechnol*. Nat Biotechnol; 2020; doi: 10.1038/s41587-020-0538-8.

6. Truvari (2020) Truvari https://github.com/spiralgenetics/truvari

7. Benson G. Tandem repeats finder: A program to analyze DNA sequences. *Nucleic Acids Res*. Nucleic Acids Res; 1999; doi: 10.1093/nar/27.2.573.
