# Supplementary material for: NPSV: A simulation-driven approach to genotyping structural variants in whole-genome sequencing data
Source: Gigascience. 2021 Jul 1;10(7):giab046. doi: 10.1093/gigascience/giab046 (PMC8246072; doi:10.1093/gigascience/giab046)
Supplement: giab046_Response_to_Reviewer_Comments_Original_Submission [file giab046_response_to_reviewer_comments_original_submission.pdf]

Author's Response To Reviewer Comments

Close

Please see uploaded document.

Close
