# Supplementary material for: NPSV: A simulation-driven approach to genotyping structural variants in whole-genome sequencing data
Source: Gigascience. 2021 Jul 1;10(7):giab046. doi: 10.1093/gigascience/giab046 (PMC8246072; doi:10.1093/gigascience/giab046)
Supplement: giab046_Response_to_Reviewer_Comments_Revision_1 [file giab046_response_to_reviewer_comments_revision_1.pdf]

Author's Response To Reviewer Comments

Close

The change tracking has been removed, ORCIDs added for all authors where available and the data availability section has been updated with the gigaDB citations.

Close
