# Supplementary material for: NPSV: A simulation-driven approach to genotyping structural variants in whole-genome sequencing data
Source: Gigascience. 2021 Jul 1;10(7):giab046. doi: 10.1093/gigascience/giab046 (PMC8246072; doi:10.1093/gigascience/giab046)
Supplement: giab046_Reviewer_1_Report_Original_Submission — Brent Pedersen -- 2/3/2021 Reviewed [file giab046_reviewer_1_report_original_submission.pdf]

Reviewer Report

Title: NPSV: A simulation-driven approach to genotyping structural variants in whole genome sequencing data

Version: Original Submission **Date:** 2/3/2021

Reviewer name: Brent Pedersen

Reviewer Comments to Author:

The authors present NPSV a tool to genotype structural variants. They evaulate it on several truthsets and show
that it performs well across SV types and sizes. The analysis is well-done and this looks to be a nice improvement in the field.
My only major comments relate to 1) evaluation and 2) usage.
1.
When genotyping large cohorts, a majority (>95% and more depending on size of cohort)
variants genotyped in each will be homozygous reference. It's unclear how this would
affect the final outcome. There is some hint of this in comparing the Polaris 2 vs
2.1 truthset where the latter has dramatically more hom-ref calls; in the 2.1, the
performance of NPSV is always better in Polaris 2.
It could be that this information can be gleaned from the many tables, but as a potential
user of this software, I'd like to be sure that the FDR will not be a problem when this is scaled to large call-sets.
2. Again, related to large cohorts, what will the run-time be on huge cohort? The run-time should
be noted more upfront as that is important; currently, it's only noted in passing in the discussion.
It's also not clear the mechanics and total run-time for a cohort. Given a cohort of $n samples and $s SVs,
is the simulation cost a one-time cost related to $s? or is it related to $n * $s? By this I mean can the simulation be done once per cohort or is it once per sample? It seems it must be done once per sample ($n * $s); if so, I'd be interested to read about possible optimizations in the discussion
that would mitigate run-time to be closer to $s.
Minor Comments
==============
+ what is purpose of extending y-axis to 60 or 70 in the scatter plots in Fig 1b? Can the y-axis be set to < 10?
+ difficult to see the black square (actual SV) in Fig 1b (this might be fixed by limiting y-axis).
+ in figure legend for 1b. can you explain why there is no apparent alt support?
+ since you are using features derived in duphold, it would be interesting to compare calls filtered as recommended by duphold (DHFFC < 0.7)
when comparing to NPSV.7). (this is not a requirement for my review, but potentially of interest)
+ as the author of duphold and creator of the DHFC metrics it would be nice to see those metrics

explicitly noted in the main text.
Software
========
It's not clear from the github (https://github.com/mlinderm/npsv) how to run the software end-to-end.
If picard metrics are not present, then one can run `npsvg preprocess` without those arguments?
Then run `npsv --stats-path $json ...`?
How does the `propose` fit in and `refine`?
A single set of commands layed out end-to-end would be helpful.

Level of Interest

Please indicate how interesting you found the manuscript: Choose an item.

Quality of Written English

Please indicate the quality of language in the manuscript: Choose an item.

Declaration of Competing Interests

Please complete a declaration of competing interests, considering the following questions:

- Have you in the past five years received reimbursements, fees, funding, or salary from an organisation that may in any way gain or lose financially from the publication of this manuscript, either now or in the future?
- Do you hold any stocks or shares in an organisation that may in any way gain or lose financially from the publication of this manuscript, either now or in the future?
- Do you hold or are you currently applying for any patents relating to the content of the manuscript?
- Have you received reimbursements, fees, funding, or salary from an organization that holds or has applied for patents relating to the content of the manuscript?
- Do you have any other financial competing interests?
- Do you have any non-financial competing interests in relation to this paper?

If you can answer no to all of the above, write 'I declare that I have no competing interests' below. If your reply is yes to any, please give details below.

I declare that I have no competing interests

I agree to the open peer review policy of the journal. I understand that my name will be included on my report to the authors and, if the manuscript is accepted for publication, my named report including any attachments I upload will be posted on the website along with the authors' responses. I agree for my report to be made available under an Open Access Creative Commons CC-BY license

(http://creativecommons.org/licenses/by/4.0/). I understand that any comments which I do not wish to be included in my named report can be included as confidential comments to the editors, which will not be published.

Choose an item.

To further support our reviewers, we have joined with Publons, where you can gain additional credit to further highlight your hard work (see: https://publons.com/journal/530/gigascience). On publication of this paper, your review will be automatically added to Publons, you can then choose whether or not to claim your Publons credit. I understand this statement.

Yes Choose an item.
