# Supplementary material for: NPSV: A simulation-driven approach to genotyping structural variants in whole-genome sequencing data
Source: Gigascience. 2021 Jul 1;10(7):giab046. doi: 10.1093/gigascience/giab046 (PMC8246072; doi:10.1093/gigascience/giab046)
Supplement: giab046_Reviewer_2_Report_Original_Submission — Arif Harmanci -- 2/6/2021 Reviewed [file giab046_reviewer_2_report_original_submission.pdf]

Reviewer Report

Title: NPSV: A simulation-driven approach to genotyping structural variants in whole genome sequencing data

Version: Original Submission **Date:** 2/6/2021

Reviewer name: Arif Harmanci

Reviewer Comments to Author:

Author's present NPSV, a del/dup genotyping method that uses simulated data to set build a training set and uses machine learning methods to genotype SVs. This is an important problem.
The manuscript is written in acceptable language but needs to be checked for grammar and typos. Authors make interesting points but they could be presented better. While the results look convincing, but the study is missing key details (details of the methodology) and comparisons that need to be added.
I added my comments below, which I hope will make the study and the presentation to improve:
0. Firstly, I would like to thank authors for making a very detailed and comprehensive github page. Just to make it a little more complete, please add a small section to download the publicly available datasets that are used in the manuscript. Please also include the command line options to regenerate the results in the paper for each result.
1. Please have the manuscript grammar checked. While it reads generally well, there are numerous typos and grammatical mistakes.
2. NPSV is a genotyping tool and does not explicitly detect SVs, which is also a very complex problem. I think this distinction should be made clear early in the manuscript. I would even recommend adding this to the name of the method, i.e. changing it to NPSVG. This distinction becomes important when NPSV is compared to other methods such as Delly which are primarily designed for joint detection+genotyping of SVs.
3. The authors should describe the limitations and strengths of each tool, for instance graphtyper2 is designed for population scale genotyping, and svviz is a visualization tool but it performs fairly well in comparisons.
4. Fig 1b. How is this figure representing the simulated data? Why is the scale of y-axis (of the alternate allele fragments) is very large? It makes it weird and hard to view the data. Also the actual data points that are supposedly colored in black are not clearly visible.
5. In Figure 1a, I find the coordinate system and the ellipses very vague, i do not understand what they represent.
6. Most of the results are for Ins/Dels but methods is allegedly for SVs? Translocations, inversions, etc? This is important again because other methods like Delly is tuned to work on complex rearrangements, i.e., that is one of their main strentgth.
7. How is the simulation strategy different from other simualtion tools? Authors need to make benchmarks of the simulation tools with reasonable comparison metrics.
8. Hyperparameters of the simulation strategy, how is ART simulator's parameters selected? These

selections must be justified. What are the parameters of NPSV's simulation strategy that impacts the accuracy?
9. AF of the SV vs the accuracy of genotyping? This can be simulated on a subsample without the SVs and remaining samples can be tested.
10. While dels and dups are separated, dups are more complex then dels: Dups can have very high copy numbers while dels can take 3 values. Very high and very low CNs? These are really the hardest CNs to genotype.
11. It would be useful to summarize the number of SVs used in benchmarks (around line 115) and SV allele frequency distributions.
12. I am surprised that the authors did not make use of 1000 Genomes call sets, is there a specific reason for this? 1kG used a complex wet lab validation process to estimate accuracy of the variant calls. I would think this is the best dataset to use.
13. Following previous comment, i am confused by HG002 analysis which states that the SVs are called by Lumpy/SVTyper and Manta. Where does the ground truth come from in these experiments? Are they validated?
14. Is there a specific set of dels/dups that NPSV fails?
15. The authors should add the SV calling and genotyping methods used in the 1000 Genomes project such as CNVnator, GenomeStrip and also VariationHunter and BreakDancer (these may not be applicable) in the comparisons.
16. It is necessary to include a time/memory benchmarking on a reasonable sized set of SVs.
17. What are the justifications of Random Forest and SVM for the classifiers? The hyperparameters should be defined and choices of the parameters should be justified.
18. What are the specific features that NPSV uses? It is not clear from the manuscript even what feature authors are referring to. The authors should clearly indicate and formulate how each feature is exactly computed. A lot of the methods must be re-written to be technically specific. I am afraid it is not beneficial unless these are defined exactly.

Level of Interest

Please indicate how interesting you found the manuscript: Choose an item.

Quality of Written English

Please indicate the quality of language in the manuscript: Choose an item.

Declaration of Competing Interests

Please complete a declaration of competing interests, considering the following questions:

- Have you in the past five years received reimbursements, fees, funding, or salary from an organisation that may in any way gain or lose financially from the publication of this manuscript, either now or in the future?

- Do you hold any stocks or shares in an organisation that may in any way gain or lose financially from the publication of this manuscript, either now or in the future?
- Do you hold or are you currently applying for any patents relating to the content of the manuscript?
- Have you received reimbursements, fees, funding, or salary from an organization that holds or has applied for patents relating to the content of the manuscript?
- Do you have any other financial competing interests?
- Do you have any non-financial competing interests in relation to this paper?

If you can answer no to all of the above, write 'I declare that I have no competing interests' below. If your reply is yes to any, please give details below.

I declare that I have no competing interests

I agree to the open peer review policy of the journal. I understand that my name will be included on my report to the authors and, if the manuscript is accepted for publication, my named report including any attachments I upload will be posted on the website along with the authors' responses. I agree for my report to be made available under an Open Access Creative Commons CC-BY license (http://creativecommons.org/licenses/by/4.0/). I understand that any comments which I do not wish to be included in my named report can be included as confidential comments to the editors, which will not be published.

Choose an item.

To further support our reviewers, we have joined with Publons, where you can gain additional credit to further highlight your hard work (see: https://publons.com/journal/530/gigascience). On publication of this paper, your review will be automatically added to Publons, you can then choose whether or not to claim your Publons credit. I understand this statement.

Yes Choose an item.
