# Supplementary material for: NPSV: A simulation-driven approach to genotyping structural variants in whole-genome sequencing data
Source: Gigascience. 2021 Jul 1;10(7):giab046. doi: 10.1093/gigascience/giab046 (PMC8246072; doi:10.1093/gigascience/giab046)
Supplement: giab046_Reviewer_3_Report_Original_Submission — Xuefang Zhao -- 2/11/2021 Reviewed [file giab046_reviewer_3_report_original_submission.pdf]

Reviewer Report

Title: NPSV: A simulation-driven approach to genotyping structural variants in whole genome sequencing data

Version: Original Submission **Date:** 2/11/2021

Reviewer name: Xuefang Zhao

Reviewer Comments to Author:

In this manuscript, Linderman et al represented a novel method, NPSV, to genotype genomic structural variants (SVs) using short-read paired-end sequencing data. Benchmark of the method using SV calls from PacBio long reads revealed improved genotype concordance of NPSV against other algorithms. Overall, I think this method is of great value. I just have a few suggestions that I hope to help further improve the manuscript.

1. NPSV genotypes the putative SVs through simulating short-read data, aligning them against the reference genome, and comparing their alignment patterns with the actual data. This approach autonomously correct for multiple confounding issues in SV genotyping, including the size, type and genomic locations. However, this approach could be computationally expensive. It would be useful to systematically benchmark the computing cost of NPSV against other algorithms, including run time, cpu usage, and estimated computing cost on cloud (if applicable).
2. NPSV showed lower genotyping accuracy for small SVs (<300bp) in tandem repeats (TRs), which is expected as short reads usually struggle in repeat sequences. Have the authors ever checked the genotype accuracy for large CNVs (typically >5Kb) that reside in segmental duplications? Segmental duplications are also of high repetitiveness, thus short-read data would have difficulty in these regions too, and it would be interesting to examine the performance of NPSV in these sequences.
3. The evaluate for offset breakpoints is informative. However, for large CNVs, the breakpoints can be off by several hundred bases to kilobases. It would be interesting to extend the experiment for evaluation of large CNVs and inversions

Minor:

1. I don't think "MEs" is defined in the manuscript?
2. It's surprising that there are 2 true-positive de novo deletions in HG002, as each genome is estimated to carry 0.2 de novo SVs (Werling et al 2018). Has any manual inspection or molecular validation done to confirm the quality of the de novo SVs, and rule out the possibility of mosaic CNVs arose during cell proliferation?

Level of Interest

Please indicate how interesting you found the manuscript: Choose an item.

Quality of Written English

Please indicate the quality of language in the manuscript: Choose an item.

Declaration of Competing Interests

Please complete a declaration of competing interests, considering the following questions:

- Have you in the past five years received reimbursements, fees, funding, or salary from an organisation that may in any way gain or lose financially from the publication of this manuscript, either now or in the future?
- Do you hold any stocks or shares in an organisation that may in any way gain or lose financially from the publication of this manuscript, either now or in the future?
- Do you hold or are you currently applying for any patents relating to the content of the manuscript?
- Have you received reimbursements, fees, funding, or salary from an organization that holds or has applied for patents relating to the content of the manuscript?
- Do you have any other financial competing interests?
- Do you have any non-financial competing interests in relation to this paper?

If you can answer no to all of the above, write 'I declare that I have no competing interests' below. If your reply is yes to any, please give details below.

I declare that I have no competing interests

I agree to the open peer review policy of the journal. I understand that my name will be included on my report to the authors and, if the manuscript is accepted for publication, my named report including any attachments I upload will be posted on the website along with the authors' responses. I agree for my report to be made available under an Open Access Creative Commons CC-BY license (http://creativecommons.org/licenses/by/4.0/). I understand that any comments which I do not wish to be included in my named report can be included as confidential comments to the editors, which will not be published.

Choose an item.

To further support our reviewers, we have joined with Publons, where you can gain additional credit to further highlight your hard work (see: https://publons.com/journal/530/gigascience). On publication of this paper, your review will be automatically added to Publons, you can then choose whether or not to claim your Publons credit. I understand this statement.

Yes Choose an item.
